# Syntheses and Reactivity of Yb and Sm Inverse Sandwich Arene Complexes

**DOI:** 10.1002/chem.202502710

**Published:** 2025-10-08

**Authors:** Sandeep Kumar Thakur, Jens Langer, Sjoerd Harder

**Affiliations:** ^1^ Inorganic and Organometallic Chemistry Friedrich‐Alexander‐Universität Erlangen‐Nürnberg Egerlandstrasse 1 91058 Erlangen Germany

**Keywords:** inverse sandwich complex, lanthanide, reactivity, samarium, ytterbium

## Abstract

Inverse sandwich arene complexes of rare earth metals have gained notable interest due to their unique electronic properties. In these complexes, an arene ligand bridges two metal centres, storing 0, 2, or 4 electrons, which is the basis for unique reactivity. Herein, we report on the reactivity of [(^DIPeP^BDI)Ln]_2_(*η* ^6^,*η* ^6^‐benzene) (**II**‐Ln, Ln = Yb or Sm) and in situ reduced [(^DIPeP^BDI)SmI]_2_ (**V**) (^DIPeP^BDI = HC[C(Me)N‐DIPeP]_2_, DIPeP = 2,6‐CHEt_2_‐phenyl). The Yb^II^‐benzene^2−^‐Yb^II^ complex (**II**‐Yb) reacted smoothly with biphenyl, naphthalene, anthracene, and COT, giving a range of new Yb^II^ complexes with bridging arene or COT ligands. However, due to much stronger Sm‐arene bonding in **II**‐Sm, which should be regarded as a Sm^III^‐benzene^4−^‐Sm^III^ complex, this reagent only reacted with substrates that can be reduced easily (anthracene and COT). Interestingly, reaction with anthracene gave a Sm^II^‐anthracene^2−^‐Sm^II^ complex, showing that the benzene^4−^ is able to reduce Sm^III^ to Sm^II^. In situ reduced [(^DIPeP^BDI)SmI]_2_ (**V**) can be considered a (^DIPeP^BDI)Sm^I^ synthon, which reacted smoothly with toluene, biphenyl, or 1,3,5‐triphenyl‐benzene and affords a series of Sm^III^‐arene^4−^‐Sm^III^ complexes. However, reaction with pyrene gave an Sm^II^‐pyrene^2−^‐Sm^II^ product. All complexes have been fully characterized with X‐ray diffraction.

## Graphical abstract

The reactivity of inverse sandwich lanthanide benzene complexes depends strongly on the metal oxidation states and consequently, the charge on the bridging benzene anion. Whereas the Yb^II^ complex reacted as a 2*e* donor with biphenyl, naphthalene, anthracene and COT, the strongly bound Sm^III^ complex only reacted with anthracene and COT.

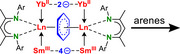

## 1 Introduction

**Scheme 1:**
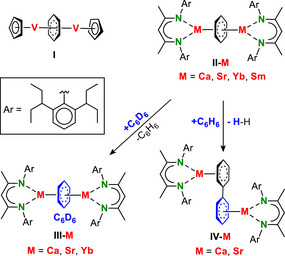
The first inverse sandwich metal benzene complex (I) and examples of group 2 and lanthanide complexes, including their reactivity.

Following the initial synthesis of the first triple‐decker inverse sandwich vanadium benzene complex (**I**, Scheme 1) in 1983,^[^ ^1^ ^]^ there has been sustained progress in the development of complexes with bridging arene ligands. To date, arene complexes of a wide variety of metals across the periodic table have been reported, including transition metals,^[^ ^2^, ^3^ ^]^ lanthanides,^[^ ^4^, ^5^, ^6^, ^7^, ^8^, ^9^, ^10^, ^11^, ^12^, ^13^, ^14^, ^15^, ^16^, ^17^, ^18^, ^19^, ^20^, ^21^, ^22^, ^23^, ^24^, ^25^, ^26^, ^27^ ^]^ actinides,^[^ ^28^, ^29^, ^30^, ^31^, ^32^, ^33^, ^34^, ^35^, ^36^, ^37^, ^38^ ^]^ and main group elements.^[^ ^39^, ^40^, ^41^, ^42^, ^43^, ^44^, ^45^, ^46^, ^47^ ^]^ A fundamental feature of these complexes is the nature and extent of metal‐arene interactions. In the case of the transition metals, the more covalently bound arene ligand generally remains electronically neutral. However, as we move toward more electropositive metals, particularly those in Groups 1 and 2, the degree of arene reduction becomes increasingly pronounced. The high negative charge on the bridging C_6_H_6_^2−^ results in diverse reactivity. It can either react as a reducing agent, a nucleophile, or a Brønsted base.^[^ ^43^, ^44^ ^]^

In contrast to *s*‐block metals, oxidation states (OSs) other than the group number are much more common for the *f*‐block metals. As the assignment of formal OSs is often troublesome, the electronic description of the bridging arene as either (arene)^0^, (arene)^2−^ or (arene)^4−^ is difficult.^[^ ^48^ ^]^ However, a combination of physical, chemical, and modern computations can aid the OS assignment and is useful to describe the electron distribution.^[^ ^49^, ^50^, ^51^ ^]^

We recently reported a set of inverse sandwich benzene complexes of the heavier alkaline‐earth (Ae) metals Ca and Sr^[^ ^42^, ^43^ ^]^ and the two lanthanide (Ln) metals Yb and Sm that can occur in the OS +II (**II**‐M in Scheme 1).^[^ ^18^ ^]^ Due to similarities in the size of the pairs Ca^II^/Yb^II^ and Sr^II^/Sm^II^, there is normally a striking similarity in their molecular structures. Whereas the structures of **II**‐Ca and **II**‐Yb show a remarkable fit, the metal‐arene distances in **II**‐Sm were found to be more than 10% shorter than those in **II**‐Sr. These differences were explained with different OS assignments: Sr^II^‐benzene^2−^‐Sr^II^ vs Sm^III^‐benzene^4−^‐Sm^III^. The high electron density on benzene^4−^ is only enabled for Sm, which has a more negative reduction potential (E^III/II^ = −1.55 V) than Yb (E^III/II^ = −1.15 V).^[^ ^52^ ^]^ This was confirmed by computational studies and recent reports of Sm inverse benzene complexes with very similar ligands and geometries.^[^ ^19^, ^20^ ^]^ Although there have been many claims for benzene^4−^ complexes.^[^ ^9^, ^10^, ^18^, ^19^, ^20^, ^21^, ^22^, ^24^, ^26^, ^27^, ^28^, ^30^, ^32^ ^]^ The experimentally found differences between **II**‐Sr and **II**‐Sm complexes are a strong argument for a 10π‐electron benzene^4−^ ligand.

These differences in electron distribution also led to differences in reactivity. The more ionically bound complexes **II**‐M (Ca, Sr, Yb) show exchange with C_6_D_6_ at room temperature to give **III**‐M. In addition, the dehydrogenative reduction of benzene to give biphenyl complex **IV**‐M is slow and unselective for Ca but fast and clean for Sr.^[^ ^43^ ^]^ However, the much stronger and more covalently bound Sm^III^ complex **II**‐Sm does not show C_6_H_6_/C_6_D_6_ exchange or biphenyl formation even at high temperature.^[^ ^18^ ^]^ The inertness of **II**‐Sm is due to the very strong Sm^III^‐benzene^4−^ bonding compared to Yb^II^‐benzene^2−^ bonding in **II**‐Yb. Although **II**‐Sm could be considered a 4*e* reducing agent and a dinuclear Sm^I^ synthon, due to its chemical inertness, it only offers limited application. Herein, we report investigations toward the reactivity of **II**‐Yb, **II**‐Sm, and in situ reduced [(^DIPeP^BDI)SmI]_2_ (**V**), which can be considered a (^DIPeP^BDI)Sm^I^ synthon, as reducing agents.

## 2 Results and Discussion

### 2.1 Reactivity of the Inverse Sandwich Benzene Ytterbium Complex **II**‐Yb

The inverse sandwich benzene ytterbium complex **II**‐Yb was observed to undergo benzene exchange reactions more rapidly than its calcium analogue **II**‐Ca but considerably slower than **II**‐Sr. Full C_6_H_6_/C_6_D_6_ exchange needs 8 days for **II**‐Ca, 12 hours for **II**‐Yb, and 5–10 minutes for **II**‐Sr.^[^ ^18^, ^43^ ^]^ Notably, unlike **II**‐Ca and **II**‐Sr, the Yb complex **II**‐Yb does not show dehydrogenative reduction of benzene to the biphenyl complex at room temperature (Scheme 1), while at higher temperature it slowly decomposes. ^[^ ^18^, ^23^ ^]^

**Scheme 2:**
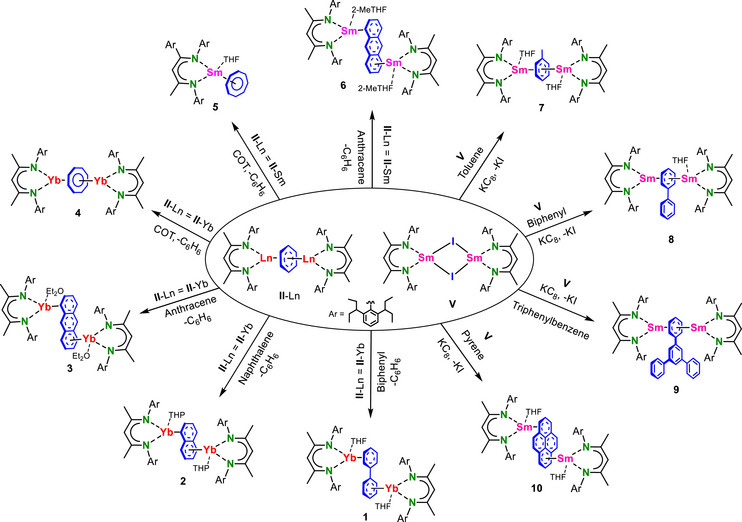
Syntheses of Yb and Sm‐arene complexes. Complexes **1**–**6** are synthesized via benzene‐to‐arene exchange reactions in Yb or Sm‐benzene complexes (**II**‐Ln). Complexes **7**–**10** are synthesized from in situ reduction of [(^DIPeP^BDI)SmI]_2_ (**V**) in the presence of various arenes or polyarenes.

**Figure 1:**
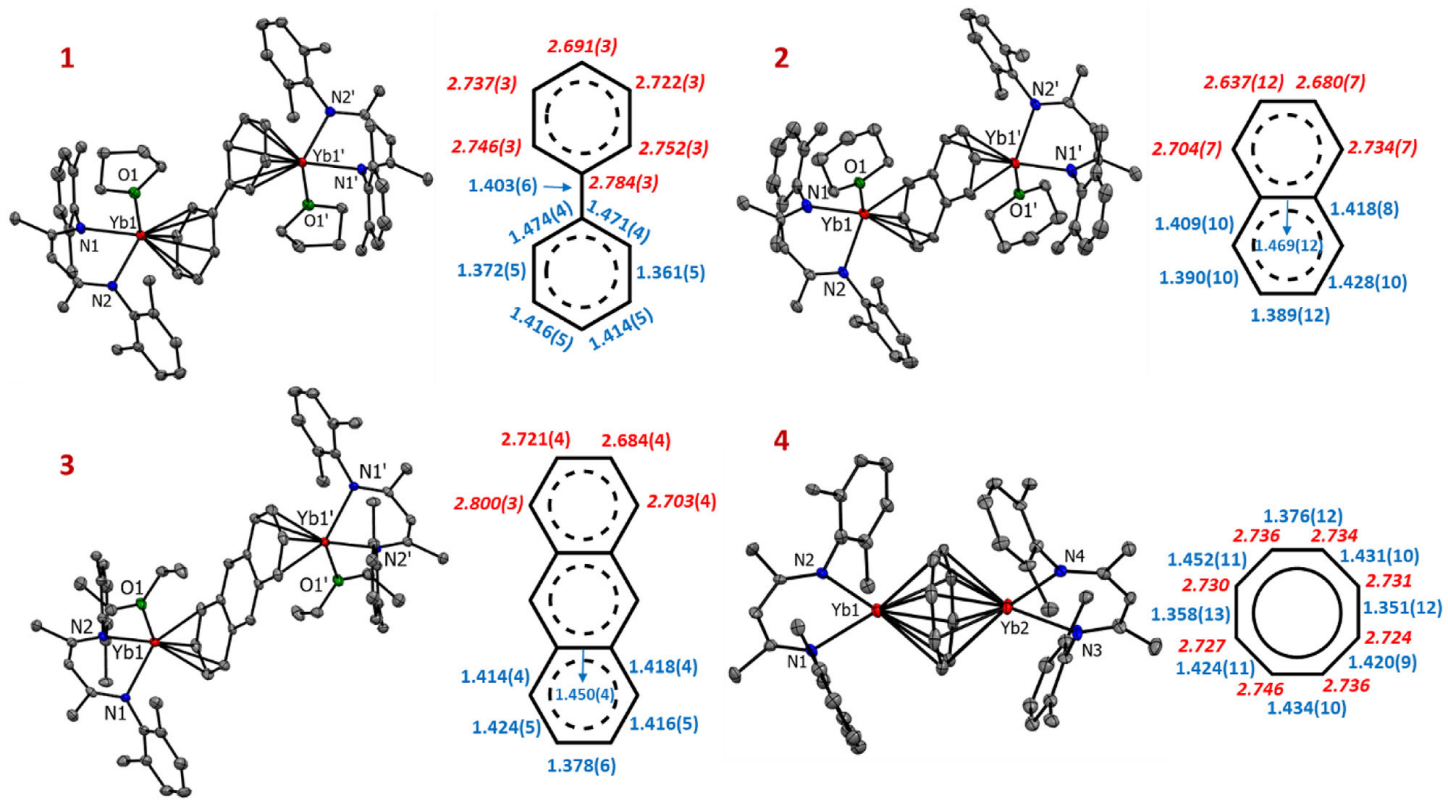
Crystal structures of Yb complexes **1–4**; H‐atoms, Et‐substituents of the ^DIPeP^BDI ligand, and H‐atoms of bridged ligand have been omitted for clarity. The inset shows the bridged ligand's M─C bond lengths in red italic [Å] and C─C bond lengths in blue [Å]. In the case of asymmetric arene bridging, we show the average M─C bonds (**4**).

However, a Yb‐biphenyl complex can be prepared via benzene to biphenyl exchange reactions (Scheme 2). The reaction of Yb‐benzene complex **II**‐Yb with biphenyl at room temperature led to a change of color from black to brown over a period of 24 hours. Full conversion was confirmed by ^1^H NMR monitoring. After the workup, an essentially pure [(^DIPeP^BDI)Yb]_2_(η^6^,η^6^‐biphenyl) was isolated in a quantitative yield. The THF adduct **1** can be crystallized overnight by adding a few drops of THF to the concentrated solution in pentane. X‐ray diffraction revealed a centrosymmetric structure with *η* ^6^‐coordination of Yb with the biphenyl ring (**1**, Figure 1). The coordination sphere of each Yb center is further saturated by a chelating ^DIPeP^BDI ligand and THF solvation. Complex **1** is isostructural to the analogue Ca complex [(^DIPeP^BDI)Ca(THF)]_2_(η^6^,η^6^‐biphenyl)^[^ ^43^ ^]^ and shows close similarities. The average Yb─N bond distance of 2.387 Å is only 0.02 Å longer than the average Ca─N bond distance (2.370 Å), which is in line with the difference in the metal cation ionic radii (Ca^2+^ 1.00 Å, Yb^2+^ 1.02 Å). The Yb‐C distances in **1** range from 2.691(3) to 2.784(3) Å. The Yb‐C_centroid_ distance of 2.344 Å is comparable to that in the corresponding Ca‐biphenyl complex (2.349 Å). The C─C bond lengths for the bridging biphenyl ligand in **1** vary from 1.361(5) to 1.474(4) Å (average: 1.418 Å). Like in the analogue Ca complex, the long‐short‐long C─C bond alteration in the rings is typical for a quinoid structure with central (Ph)C═C(Ph) bond character and formal negative charges at the remote *para*‐C atoms. Indeed, for the Ca analogue of **1**, the highest negative charge was calculated for the *para*‐C atoms, which also show the shortest metal‐C bond distances.^[^ ^43^ ^]^ This is in agreement with a dianionic biphenyl system and assignment of Yb^II^‐biphenyl^2−^‐Yb^II^ OSs.

In these bimetallic M‐biphenyl‐M complexes, the coordinated phenyl ring is almost planar. This contrasts with bonding in a monometallic Y‐biphenyl anion, (Me_3_SiCp)_2_Y(η^6^‐biphenyl)¯, in which the coordinated phenyl ring is in boat form with the shortest Y‐C distance to the *para*‐C (2.480(2) Å) and the *ipso*‐C (2.561(2) Å).^[^ ^13^ ^]^

The ^1^H NMR spectrum of **1** (Figure S1) displays three resonances at 4.91, 3.97, and 3.52 ppm that correspond to the coordinated biphenyl ligand and one signal at 3.04 ppm attributed to the benzylic Et_2_C*H* protons of the ^DIPeP^BDI ligand, indicating on average a highly symmetric structure. Whereas the signals for **1** are well‐behaved and can be easily assigned, those in the corresponding Ca complex are much broader, pointing toward higher dynamics in the case of the Ca‐biphenyl complex.

The reaction of the Yb‐benzene complex **II**‐Yb with naphthalene or anthracene led to an immediate color change from black to dark blue (Scheme 2). The spectroscopic data showed full consumption of starting materials and quantitative formation of the Yb‐naphthalene complex [(^DIPeP^BDI)Yb]_2_(*η* ^4^,*η* ^4^‐C_10_H_8_) or the Yb‐anthracene complex [(^DIPeP^BDI)Yb]_2_(*η* ^4^,*η* ^4^‐C_14_H_10_), respectively. The clean formation of these complexes can be easily followed by monitoring the ^1^H NMR signals for the ^DIPeP^BDI backbone C*H* group, which show indicative singlets at 5.14 ppm (Figure S6) or 5.06 ppm (Figure S12), respectively. The presence of two triplets for CH(CH_2_C*H*_3_)_2_, two multiplets for CH(C*H*_2_CH_3_)_2_, and only one multiplet for C*H*(CH_2_CH_3_)_2_ in both complexes indicates their symmetric nature in solution.

Adding a few drops of tetrahydropyran (THP) to a concentrated pentane solution of the Yb‐naphthalene complex [(^DIPeP^BDI)Yb]_2_(*η* ^4^,*η* ^4^‐C_10_H_8_) led to rapid crystallization of the THP‐adduct **2**. X‐ray diffraction revealed a centrosymmetric inverse sandwich structure featuring an *η* ^4^,*η* ^4^‐bridging naphthalene dianion and a coordinated THP ligand (**2**, Figure 1). The structure compares well with that of a very similar, previously reported Yb‐naphthalene complex with a ^DIPP^BDI ligand instead of the bulkier ^DIPeP^BDI ligand used in our work (DIPP = 2,6‐*i*Pr‐phenyl).^[^ ^53^ ^]^ The bridging naphthalene dianion resides on an inversion centre, with two (^DIPeP^BDI)Yb⁺ fragments symmetrically coordinated to opposite faces of the two rings. The geometry of the bridging naphthalene ligand is also similar to that in a reported Ca‐naphthalene complex, [(^DIPeP^BDI)Ca]_2_(*η* ^4^,*η* ^4^‐C_10_H_8_),^[^ ^47^ ^]^ and exhibits typical structural features of dianionic naphthalene. Both 6‐membered rings are deformed to nonplanar boat structures. The metals coordinate to the outer parts of the naphthalene dianion. Due to disorder in the bridging naphthalene and the ^DIPeP^BDI ligands, large standard deviations in the bond distances do not allow for a detailed discussion of the geometry.

In a similar fashion, the ether adduct of the Yb‐anthracene complex (**3**) was obtained by addition of Et_2_O to a concentrated pentane solution. Complex **3** crystallized as a centrosymmetric inverse sandwich with the *η* ^4^,*η* ^4^‐bridging anthracene dianion over an inversion center (**3**, Figure 1). Two (^DIPeP^BDI)Yb⁺ cations coordinate to the outer rings of the anthracene dianion (C_14_H_10_^2−^) from opposite faces. In contrast to the naphthalene ligand in **2**, the anthracene ligand remains nearly planar. This is likely due to enhanced charge delocalization across anthracene's extended π‐system. The structure compares well with a similar previously reported Yb‐anthracene complex with a ^DIPP^BDI ligand instead of a ^DIPeP^BDI ligand.^[^ ^53^ ^]^ The C─C bond lengths in the anthracene unit vary between 1.378(6) Å and 1.450(4) Å, and the Yb‐C are in the 2.684(4)‐2.800(3) Å range (average: 2.727 Å).

We also investigated the reactivity of the Yb‐benzene complex **II**‐Yb with cyclooctatetraene (COT). The C_6_H_6_^2−^ unit in the Yb‐benzene complex serves as a two‐electron reducing agent and rapidly releases free benzene and two electrons after the addition of COT. Two‐electron reduction of COT leads to an aromatic 10π‐electron COT^2−^ dianion. The ^1^H NMR spectrum of the raw product displays resonances at 4.44 ppm corresponding to the ^DIPeP^BDI ligand backbone C*H* and a signal at 5.34 ppm attributed to the COT^2−^ligand (Figure S17). Crystallization from a concentrated pentane solution afforded single crystals of [(^DIPeP^BDI)Yb]_2_(*η* ^8^,*η* ^8^‐C_8_H_8_) (**4**). The structure of **4** is isostructural to the analogue Ca complex^[^ ^44^ ^]^ and compares well to that of a similar Yb‐COT complex.^[^ ^53^ ^]^ The asymmetric unit contains two similar molecules, both exhibiting an inverse‐sandwich structure, in which a planar C_8_H_8_^2−^dianion is symmetrically bridging two (^DIPeP^BDI)Yb⁺ cations in (*η* ^8^,*η* ^8^)‐fashion. The Yb‐C bond distances are in the range of 2.685(8)‐2.781(7) Å (average: 2.732 Å) and are comparable to those observed in the Ca‐COT analogue (2.738 Å),^[^ ^44^ ^]^ or to those in other Yb^II^ complexes with bridging COT ligands.^[^ ^53^, ^54^, ^55^, ^56^ ^]^ Exposure of **4** to excess COT combined with prolonged heating (60 °C, overnight) led to unselective oxidation to Yb^III^, as evidenced by the emergence of paramagnetic features in the NMR spectrum (Figure S18). However, we were not able to isolate any defined products out of this mixture.

### 2.2 Reactivity of Inverse Sandwich Benzene Samarium Complex **II**‐Sm or an in Situ Prepared Samarium(I) Complex

**Figure 2:**
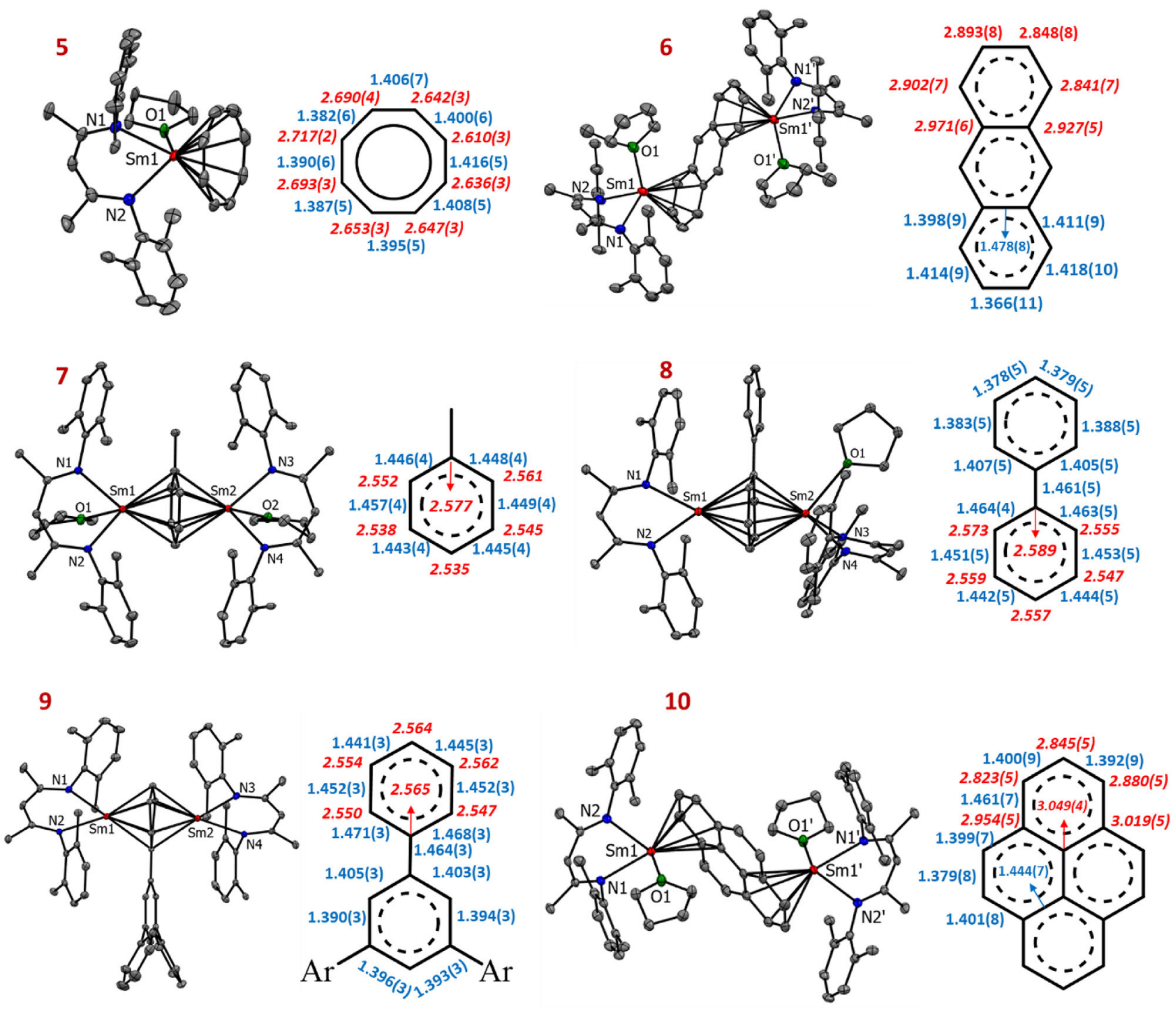
Crystal structure of Sm complexes **5–10**; H‐atoms, Et‐substituents of the ^DIPeP^BDI ligand, and H‐atoms of the bridged ligand have been omitted for clarity. The inset shows the bridged ligand's M─C bond lengths in red italic [Å] and C─C bond lengths in blue [Å]. In the case of asymmetric arene bridging, we show the average M─C bonds (**7**–**9**).

In our previous work,^[^ ^18^ ^]^ we highlighted the distinct electronic characteristics of inverse sandwich benzene complexes of ytterbium and samarium. These differences were further reflected in their preliminary reactivity profiles, which underscored notable divergence and encouraged us to further explore their chemical behaviour. The Yb‐benzene complex **II**‐Yb demonstrated the typical 2*e*‐reducing reactivity that we also observed for **II**‐Ca, which reacts as a Ca^I^ synthon by releasing benzene and two electrons.^[^ ^43^, ^44^ ^]^ Whereas complex **II**‐Yb underwent clean arene exchange reactions with biphenyl, naphthalene, anthracene, and cyclooctatetraene (COT), the Sm‐benzene complex **II**‐Sm remained unreactive toward biphenyl and naphthalene, even upon prolonged heating at 100 °C (24 hours). However, it reacted with two equivalents of COT and formed a half‐sandwich Sm‐COT complex (Scheme 2), which could be crystallized as its THF adduct (^DIPeP^BDI)Sm(*η* ^8^‐COT)·(THF) (**5**) in quantitative yield. Following a different synthetic procedure, a similar complex was obtained by Anker and coworkers.^[^ ^20^ ^]^ Single‐crystal X‐ray diffraction confirmed its mononuclear structure, featuring a (^DIPeP^BDI)Sm^III^ fragment coordinated to a COT^2−^ dianion through *η* ^8^‐interactions (**5**, Figure 2). Additionally, one molecule of THF is coordinated to the samarium center, imparting a geometry reminiscent of typical half‐sandwich complexes. The average Sm‐C bond length of 2.661 Å is only slightly longer than those reported for THF‐free (^DIPeP^BDI)Sm‐COT (2.629 Å) or (BDI*)Sm‐COT (2.636 Å) complexes (BDI* = CH[C(Me)N‐Dicyp]_2_, Dicyp = 2,6‐dicyclohexylphenyl).^[^ ^19^, ^20^ ^]^

Complex **II**‐Sm also reacted selectively with anthracene at room temperature. A ^1^H NMR spectrum of the crude reaction product showed full consumption after 1.5 hours and a mixture of broad and sharp signals over a range of 26.1 to −14.9 ppm (Figures S30–S32). Single crystals suitable for X‐ray diffraction were grown by adding a few drops of 2‐Me‐THF to a concentrated pentane solution, and the product crystallized as the 2‐Me‐THF adduct (**6**, Figure 2). Two (^DIPeP^BDI)Sm units coordinate to the outer rings of the anthracene from opposite faces via *η* ^6^,*η* ^6^‐coordination. Similar as in complex **3**, the bridged anthracene ligand in **6** remains nearly planar. The Sm‐C distances vary from 2.841(7) to 2.971(6) Å with an average of 2.897 Å. The Sm‐C bond distance is very close to the Sr‐C distances in the Sr‐arene complexes **II**‐Sr (2.829 Å) and **IV**‐Sr (2.866 Å). The close similarity of Sm‐C and Sr‐C bond lengths indicates the presence of a Sm^II^‐anthracene^2−^‐Sm^II^ arrangement. To confirm this charge distribution and OS assignment, we measured the magnetic moment by Evans methods.^[^ ^57^, ^58^ ^]^ For a binuclear Sm^II^‐anthracene^2−^‐Sm^II^ complex, the predicted susceptibility at 300 K would be χ_P_T = 3.19 cm^3^ mol^−1 ^K whereas for Sm^III^‐anthracene^4−^‐Sm^III^ complex, a value of χ_P_T = 0.786 cm^3^ mol^−1 ^K would be expected.^[^ ^18^ ^]^ For complex **6**, a susceptibility of χ_P_T = 3.15 cm^3^mol^−1 ^K was observed, which is in close agreement with the formation of Sm^II^‐ anthracene^2−^‐Sm^II^ complex. It is of interest to note that the tetraanionic benzene ligand in the Sm^III^ benzene complex **II**‐Sm not only reduces anthracene to anthracene^2−^ but is also able to reduce Sm^III^ to Sm^II^.

The Sm‐benzene complex (**II**‐Sm) remains unreactive toward both biphenyl and naphthalene even under forced conditions. In contrast, it reacts selectively with COT and anthracene, likely due to their low reduction potentials of −1.62 versus SCE and −2.12 V versus SCE, respectively (Table S1). Attempts to induce benzene to arene substitution in **II**‐Sm using more forcing conditions typically resulted in the formation of multiple unidentified decomposition products. Alternatively, Sm‐arene inverse sandwich complexes can be accessed by in situ reduction of [(^DIPeP^BDI)SmI]_2_ (**V**) in the presence of various arene or polyarene ligands. As described for the synthesis of **II**‐Sm, reduction of **V** using potassium graphite in toluene led to the clean and quantitative formation of the inverse sandwich Sm‐toluene complex [(^DIPeP^BDI)Sm]_2_(*η* ^6^,*η* ^6^‐C_7_H_8_) as a black powder. Although the product is paramagnetic, all the ^1^H NMR signals could be identified (Figure S33). The ^1^H NMR spectrum of the product exhibited three signals between 22.7 and 21.5 ppm, attributed to the aromatic protons of the sandwiched toluene, along with a sharp singlet at −12.3 ppm corresponding to its methyl group (Figure S33). Apart from this, the backbone C*H* group of ^DIPeP^BDI shows a singlet at 10.6 ppm.

Single crystals of the THF‐adduct (**7**) suitable for X‐ray diffraction analysis were obtained by vapor diffusion of THF into a concentrated pentane solution. The crystal structure of **7** revealed *η* ^6^,*η* ^6^‐coordination of a nearly planar C_7_H_8_ ligand and two ^DIPeP^BDI‐Sm fragments (Figure 2). Notably, the toluene ligand exhibits minimal variation in C─C bond lengths, ranging from 1.443(4) to 1.457(4) Å, indicative of a delocalized electronic structure. The average Sm‐C bond distance was determined to be 2.551 Å. Given the comparable ionic radii of Sr^2+^ and Sm^2+^, the Sm‐C bond length is significantly shorter than that observed in the Sr^II^‐benzene complex (**II**‐Sr) (2.829 Å), however, it closely aligns with the bond distance reported for the Sm^III^ complexes [(^DIPeP^BDI)Sm]_2_(*η* ^6^,*η* ^6^‐C_6_H_6_) (2.532 Å) and [(BDI*)Sm]_2_(*η* ^6^,*η* ^6^‐C_7_H_8_) (2.543 Å).^[^ ^19^, ^20^ ^]^ These structural features indicate oxidation of Sm^II^ to Sm^III^, accompanied by the formation of a bridging tetra‐anionic toluene ligand (C_7_H_8_^4−^).

Furthermore, reduction of [(^DIPeP^BDI)SmI]_2_ (**V**) in the presence of biphenyl in hexane resulted in the quantitative formation of the inverse sandwich biphenyl complex (^DIPeP^BDI)Sm(*μ*‐biphenyl)Sm(^DIPeP^BDI) (Scheme 2). Crystals of a mono‐THF adduct (**8**), which were suitable for X‐ray diffraction, were obtained by slow diffusion of THF into a concentrated pentane solution of the raw product. This led to crystallization of an asymmetric dinuclear complex in which only one of the Sm metals shows THF coordination. The solid‐state structure revealed *η* ^6^,*η* ^6^‐coordination of one of the two aromatic rings of the biphenyl to both Sm metals. The geometry around the two Sm centers is very much similar to an earlier reported Sm^III^‐biphenyl complex in which both Sm metals sandwich one of the two Ph rings: [(LSm)_2_(*η* ^6^,*η* ^6^‐biphenyl)^2^¯](K^+^)_2_ in which L = [Me_2_ *t*BuSiN)Cp]_2_Fe.^[^ ^11^ ^]^ The Sm‐coordinated ring displays elongated C─C bond lengths averaging 1.453 Å, while the noncoordinated ring retained normal C─C bond lengths (average: 1.390 Å). This is consistent with charge localization and reduction of one of the rings. The geometry of the coordinated ring lies between chair and half‐chair conformations, whereas the unbound ring remains planar. The average Sm‐C bond length was found to be 2.563 Å, which is significantly shorter than the analogous Sr‐C bond length (2.866 Å) in a similar Sr‐biphenyl complex.^[^ ^43^ ^]^ This hints to a Sm^III^‐biphenyl^4−^‐Sm^III^ arrangement, which is supported by similar Sm‐C bond distances in Sm^III^‐benzene^4−^‐Sm^III^ complexes, ranging from 2.462(7) to 2.602(2).^[^ ^18^, ^19^, ^20^ ^]^ It also compares well to a heterobimetallic Sm/K complex containing tetra‐anionic biphenyl^4−^ (average Sm‐C: 2.596 Å).^[^ ^11^ ^]^ The 4‐character of the bridging biphenyl ligand is also evident from the fact that both Sm atoms coordinate to the same Ph ring, leaving the other ring untouched. This results in two aromatic rings, one 10π‐electron ring with a 4‐ charge and one neutral 6π‐electron system. In contrast, the biphenyl dianion in **1** avoids such bridging and shows coordination of the Yb metals to different rings. In this case, the biphenyl^2−^ dianion has a quinoid‐type structure with alternating long and short C─C bond distances. The shortest Yb‐C distances are observed to the *para*‐C atoms, which carry most of the negative charge.

The symmetric crude reaction product [(^DIPeP^BDI)Sm]_2_(η^6^,η^6^‐biphenyl) was found to be essentially pure and was studied in solution by NMR. Its ^1^H NMR spectrum in C_6_D_6_ exhibited relatively sharp resonances in the range of 11 to ‐5 ppm (Figure S39), in contrast to the broader signals typically associated with Sm^II^ complexes. Notably, signals for the biphenyl ligand were not observed. This may be due to dynamic Sm‐biphenyl coordination. Indeed, cooling a toluene‐*d*_8_ solution of the product to −30 °C led to slow exchange and appearance of distinct signals at 28.4, 22.5, and 13.3 ppm, which can be assigned to the biphenyl moiety. (Figure S40).

The in situ reduction strategy also proved to be effective for bulkier arenes, such as 1,3,5‐triphenylbenzene and pyrene, yielding the corresponding inverse sandwich complexes [(^DIPeP^BDI)Sm]_2_(η^6^,η^6^–1,3,5‐triphenylbenzene) and [(^DIPeP^BDI)Sm]_2_(η^6^,η^6^‐pyrene). Reduction of the [(^DIPeP^BDI)SmI]_2_ (**V**) with 0.5 equivalents of 1,3,5‐triphenylbenzene in hexane resulted in complete conversion to the reduced species within 5 hours. The ^1^H NMR spectrum of the crude product displayed well‐resolved signals in the range of 28 to ‐5 ppm, consistent with the selective formation of one product (Figure S46). The number of signals excludes bridging of the central arene ring and is consistent with asymmetric bridging of the triphenylbenzene ligand. Crystallization from pentane at −30 °C under an inert atmosphere afforded X‐ray quality crystals of the product (**9**) which indeed revealed *η* ^6^,*η* ^6^‐coordination of one of the peripheral phenyl rings of the 1,3,5‐triphenylbenzene ligand by two (^DIPeP^BDI)Sm fragments (**9**, Figure 2). As anticipated, the Sm‐bound ring displayed elongated C─C bond lengths averaging 1.455 Å, indicative of an arene^4−^ system with charge delocalization, while the uncoordinated phenyl rings retained typical aromatic bond lengths of 1.381–1.406 Å. The average Sm‐C bond length of 2.557 Å is consistent with values observed in Sm‐biphenyl (**7**: 2.563 Å) and Sm‐benzene (**II**‐Sm: 2.532 Å) complexes, suggesting a Sm^III^‐arene^4−^‐Sm^III^ arrangement.

Similarly, Sm‐pyrene was synthesized by reduction of [(^DIPeP^BDI)SmI]_2_ (**V**) in the presence of pyrene (Scheme 2). Crystallization of the product in the form of a THF‐adduct (**10**) was achieved by the addition of a few drops of THF to a concentrated pentane solution. The complex was conclusively characterized by single‐crystal X‐ray diffraction and elemental analysis. The solid‐state structure of complex **10** revealed *η* ^6^,*η* ^6^‐coordination of the least‐substituted, nonadjacent rings of the pyrene ligand by two (^DIPeP^BDI)Sm units, forming a symmetric inverse sandwich motif. Lanthanide complexes with reduced pyrene anions can have a wide range of coordination modes and show different charges varying from ‐1, ‐2 to ‐3.^[^ ^59^ ^]^ The reduction of pyrene to a tetra‐anion is known to be challenging.^[^ ^60^ ^]^ It is therefore doubtful whether we have Sm^II^‐pyrene^2−^‐Sm^II^ or a Sm^III^‐pyrene^4−^‐Sm^III^ arrangement. The average Sm‐C bond distance in **10** (2.916 Å) is significantly longer than those observed in typical Sm^III^‐arene^4−^‐Sm^III^ complexes **7**–**9** (2.510 (2)‐2.619 (4) Å) but very close to Sm^II^‐anthracene complex **6** (2.897 Å). Also, the average Sm‐N distance in **10** (2.520 Å) is significantly longer than the Sm^III^‐N distance in **II**‐Sm (2.454 Å). Both arguments are in favor of an Sm^II^‐pyrene^2−^‐Sm^II^ arrangement.

The ^1^H NMR spectrum of the isolated product exhibited broad resonances across a wide range (29 to −6 ppm) (Figure S53). As variable temperature ^1^H NMR from −80 °C to +60 °C did not lead to decoalescence or significant sharpening of the signals (Figure S54), the poorly resolved NMR spectrum is not due to dynamic processes but is consistent with strong paramagnetism of Sm^II^ metal centres, hampering a more detailed characterization by NMR spectroscopy. To confirm this charge and OS assignment, we measured the magnetic moment by the Evans method.^[^ ^57^, ^58^ ^]^ For a binuclear Sm^II^‐pyrene^2−^‐Sm^II^ complex, the predicted susceptibility at 300 K would be χ_P_T = 3.19 cm^3^ mol^−1 ^K, whereas for Sm^III^‐pyrene^4−^‐Sm^III^ complex, a value of χ_P_T = 0.786 cm^3^ mol^−1 ^K would be expected.^[^ ^18^ ^]^ For complex **10**, at 300 K a susceptibility of χ_P_T = 2.88 cm^3^mol^−1 ^K was observed. All data are in agreement with the formation of a Sm^II^‐pyrene^2−^‐Sm^II^ complex.

## 3 Conclusions

We explored the reactivity of the inverse sandwich benzene complexes (^DIPeP^BDI)Ln(*η* ^6^,*η* ^6^‐ benzene)Ln(^DIPeP^BDI) (**II**‐Ln) with Ln = Yb or Sm and in situ reduced [(^DIPeP^BDI)SmI]_2_ (**V**), which can be considered a (^DIPeP^BDI)Sm^I^ synthon. Exploiting the similarities in the chemistry of Ca^II^ and Yb^II^ or Sr^II^ and Sm^II^ gave insight into the electronic distribution and metal OSs.

Whereas the Yb‐benzene complex **II**‐Yb showed two‐electron reducing reactivity and was able to doubly reduce biphenyl, naphthalene, anthracene, and COT, resulting in a range of inverse arene sandwich complexes (**1**–**4**), the analogue Sm complex **II**‐Sm only showed reactivity with anthracene and COT, the two substrates that can be reduced most easily. This difference in reactivity is explained by the difference in bonding and OS's of the metals in **II**‐Yb and **II**‐Sm. Complex **II**‐Yb is best represented with a Yb^II^‐benzene^2−^‐Yb^II^ arrangement, whereas **II**‐Sm should be regarded as a Sm^III^‐benzene^4−^‐Sm^III^ complex. Very strong and mainly electrostatic bonding in **II**‐Sm makes this complex relatively inert toward arene exchange reactions. However, when reacted with COT, an organic ring that is easily reduced to the aromatic 10π‐electron COT^2−^ anion, **II**‐Sm reacted like a Sm^I^ synthon and donated four electrons to give two equivalents of the Sm^III^ complex (^DIPeP^BDI)Sm(COT) (**5**). In contrast, **II**‐Yb was found to be only a 2*e* donor and gave the binuclear complex [(^DIPeP^BDI)Yb]_2_(η^8^,η^8^‐COT) (**4**). Complex **II**‐Sm also reacted with anthracene, but in this case, a Sm^II^ species was formed: [(^DIPeP^BDI)Sm(2‐Me‐THF)]_2_(η^6^,η^6^‐anthracene) (**6**). This means that the bridging benzene^4−^ ligand in **II**‐Sm can act as a reducing agent for the Sm^III^ → Sm^II^ transition, a reduction that is generally performed with potent reducing agents like K^0^.

Alternatively, the inverse arene Sm complexes can be synthesised by the reduction of [(^DIPeP^BDI)SmI]_2_ (**V**) with KC_8_, resulting in precipitation of KI. *In‐situ* prepared “(^DIPeP^BDI)Sm” may be formulated as a Sm^I^ intermediate, but caution is advised. In view of very recent work on lanthanide centers in low OSs,^[^ ^25^, ^61^ ^]^ the ^DIPeP^BDI ligand may have been (partially) reduced. However, the product of the reduction of **V** could be considered an Sm^I^ synthon. Reduction of **V** in the presence of toluene, biphenyl, 1,3,5‐triphenylbenzene, or pyrene led to the formation of complexes **7**–**10**. Whereas complexes with bridging toluene, biphenyl, or 1,3,5‐triphenylbenzene ligands should be regarded to have a Sm^III^‐arene^4−^‐Sm^III^ arrangement, like the anthracene ligand in **6** the bridging pyrene in **10** is only doubly reduced, and both Sm centers are in OS +II. The formation of Sm^II^‐ or Sm^III^‐arene complexes depends on the stability of dianionic or tetra‐anionic arenes in combination with bonding to the corresponding Ln^II^ or Ln^III^ centers.

## Supporting Information

Selected NMR spectra, IR‐ and UV‐spectra, crystallographic details including ORTEP representations for all crystal structures. Deposition Numbers “2454435 (for **1**), 2454436 (for **2**), 2454437 (for **3**) 2454438 (for **4**), 2454439 (for **5**) 2481810 (for **6**) 2454440 (for **7**) 2454441 (for **8**) 2454442 (for **9**) and 2454443” (for **10**)</url>contain the supplementary crystallographic data for this paper. These data are provided free of charge by the joint Cambridge Crystallographic Data Centre and Fachinformationszentrum Karlsruhe “Access Structures service”.

## Conflict of Interest

The authors declare no competing financial interest.

## Supporting information



Supporting Information

Supporting Information

## Data Availability

The data that support the findings of this study are available in the supplementary material of this article.
