# Supplementary material for: Syntheses and Reactivity of Yb and Sm Inverse Sandwich Arene Complexes
Source: Chemistry. 2025 Oct 8;31(62):e02710. doi: 10.1002/chem.202502710 (PMC12598387; doi:10.1002/chem.202502710)
Supplement: Supplementary file 1 — Supporting Information [file CHEM-31-e02710-s002.pdf]

Supporting Information

Table of Contents

1. Materials and methods S2

2. Complex syntheses and reactivity S3

3. Spectroscopic data S13

   3a. NMR data S13

   3b. IR data S40

   3c. UV-vis data S45

4. Crystal structure determination S50

5. References S58

1. Materials and methods

All experiments were conducted in dry glassware under an inert nitrogen atmosphere by applying standard Schlenk techniques or gloveboxes (MBraun) using freshly dried and degassed solvents. All solvents were degassed with nitrogen, dried over activated aluminum oxide (Innovative Technology, Pure Solv 400-4-MD, Solvent Purification System), and then stored under inert atmosphere over molecular sieves (3 Å) unless noted otherwise. Deuterated benzene (C_6D_6) was purchased from Sigma Aldrich or Deutero GmbH, degassed and dried over molecular sieves (3 Å). 2,6-CH(Et_2)-aniline was synthesized according to a slightly modified literature procedure.[1] The following compounds were synthesized according to literature procedures: [(^{Dipep}BDI)M]$_2$(C_6H_6), M = Yb, Sm] and (^{DIPeP}BDI)SmI.[2] Biphenyl, naphthalene, anthracene were purchased from Sigma Aldrich and sublimed under reduced pressure before the use. Cyclooctatetraene (COT) was purchased from Alpha Asear and was stirred over calcium hydride, distilled under N_2 atmosphere and stored over molecular sieves 3 Å. KN($SiMe_3$)$_2$, Pyrene, 1,3,5-triphenyl benzene were purchased from Sigma-Aldrich and was used without further purification. It was found that ether-free products crystallized extremely slow or often not at all. The addition of small quantities of ethers led in nearly all cases to rapid crystallization which helped purification and enabled characterization by XRD.

NMR spectra were measured on Bruker Avance III HD 400 MHz and Bruker Avance III HD 600 MHz spectrometers. Chemical shifts (δ) are denoted in ppm (parts per million), coupling constants in Hz (Hertz). For describing signal multiplicities common abbreviations are used: s (singlet), d (doublet), t (triplet), q (quartet), p (quintet), sept (septet), m (multiplet) and br (broad). Spectra were referenced to the solvent residual signal. Assignments of resonance signals in the ^{1}H and ^{13}C{^{1}H} NMR spectra were made based on two-dimensional NMR correlation (HSQC, HMBC, COSY) experiments. Elemental analysis was performed with a Hekatech Eurovector EA3000 analyzer.

Infrared spectra were acquired on a Bruker Alpha II FT-IR spectrometer equipped with a Platinum ATR diamond from the neat compounds under inert conditions inside a glovebox. All spectra were recorded at room temperature in the range of 400–4000 cm^{-1} with a resolution of 4 cm^{-1} and baseline corrected. Solution state UV-vis spectra of **1-10** complexes were measured on an Agilent Technologies Cary 60 UV-vis spectrophotometer. The crystal structure data has been deposited with the Cambridge Crystallographic Data Centre. This data can be obtained free of charge from The Cambridge Crystallographic Data Centre via www.ccdc.cam.ac.uk/data_request/cif. Crystallographic and refinement data and CCDC numbers of **1**-**10** compounds are summarized in Table S3-6.

2. Complex syntheses and reactivity

General remark: The reactivity of the arenes is determined by their reduction potentials. Table S1 gives an overview of the reduction potential of the reagents used in this study.

Table S1: Reduction potential of hydrocarbons *vs* Saturated calomel electrode (SCE)

**Hydrocarbon**	**Reduction potential *vs* SCE (1st, 2nd)**
COT[3]	-1.62, -1.86
Anthracene[4]	-2.12, -2.72
Pyrene[4]	-2.21, -2.94
Triphenyl benzene[5]	-2.50
Naphthalene[4]	-2.61
Biphenyl[4]	-2.76, -3.26
Benzene[4]	-3.43

Synthesis of $[(^{DIPeP}BDI)Yb(THF)]_2(\eta^6,\eta^6$-biphenyl) (1)

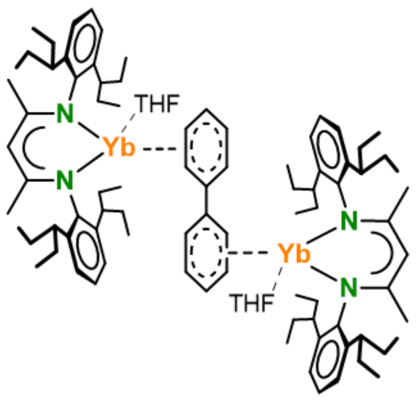

$[(^{Dipep}BDI)Yb]_2(C_6H_6)]$ (0.100 g, 0.067 mmol) and biphenyl (0.01g, 0.067 mmol) were suspended in cyclohexane (5.0 mL) and stirred at room temperature. After stirring the reaction mixture for 24 h at room temperature, the colour changed from black to brown. Quantitative conversion to $[(^{DIPeP}BDI)Yb]_2(\eta^6,\eta^6$-biphenyl) was observed by NMR spectroscopy. After the reaction the solvent was removed in *vacuo*, yielding essentially pure $[(^{DIPeP}BDI)Yb]_2(\eta^6,\eta^6$-biphenyl) (0.099 g, 0.063 mmol, 94%) as dark brown powder. Crystals suitable for single crystal X-ray diffraction, were obtained by adding few drops of THF to a concentrated pentane solution at -35 °C affording THF adduct **1**.

^{1}H NMR (C_7D_8, 600.13 MHz, 298 K): δ = 6.99-6.95 (m, 12H, C**H**-arom), 4.92-4.89 (m, 4H, C**H**-biphenyl), 4.74 (s, br, 2H, C**H**-backbone), 4.19 (s, br, 8H, C**H$_2$**-THF), 3.98-3.96 (m, 4H, C**H**-biphenyl), 3.52 (s, br, 2H, C**H**-biphenyl) 3.04 (s, br, 8H, C**H**), 1.70-1.60 (m, 40H, C**H**$_2$ and 8H, C**H$_2$**-THF), 1.59 (s, 12H, C**H**$_3$-backbone), 0.91-0.87 (m, 24H, C**H**$_3$), 0.84-0.80 (m, 24H, C**H**$_3$) ppm.

^{13}C NMR (C_6D_6, 151 MHz, 298 K): δ = 165.6 (**C**N-backbone), 149.6 (**C**-arom), 139.9 (**C**-arom), 138.0 (**C**-biphenyl) 126.1 (**C**-arom), 122.5 (**C**-arom), 105.3 (**C**H-biphenyl), 93.4 (**C**H-backbone), 83.5 (**C**H- biphenyl),

70.6 (C**H**$_2$-THF), 40.1 (**C**H), 26.6, 26.3, 26.1, 25.6 (**C**H$_2$), 25.3 (**C**H$_3$-backbone), 14.3, 13.6, 12.1, 10.8 (**C**H$_3$) ppm.

FT-IR (ATR, pure): $\tilde{\nu}$ = 2955, 2927, 2869, 1553, 1524, 1506, 1456, 1395, 1375, 1338, 1325, 1270, 1242, 1217, 1161, 1098, 1067, 1019, 998, 957, 919, 897, 868, 789, 768, 730, 654, 603, 524.

Elemental analysis calculated for $C_{94}H_{140}N_4O_2Yb_2$ (M = 1704.29 g/mol): C 66.25, H 8.28, N 3.29 % with two molecules of coordinated THF. Found: C 64.57, H 7.89, N 3.42 %. Although the C value is likely too low due to metal carbide formation, it is provided to illustrate the best values obtained to date.

Synthesis of [(^{DIPeP}BDI)Yb(THP)]$_2$(η^4,η^4-naphthalene) (2)

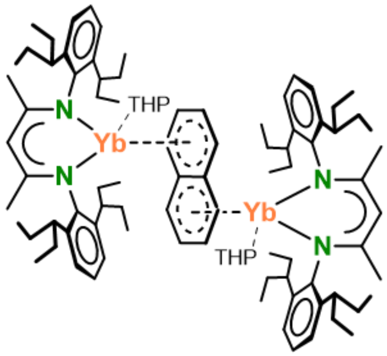

In a J-Young NMR tube, [(^{Dipep}BDI)Yb]$_2$(C_6H_6)] (20 mg, 13.5 µmol, 1.0 eq.) and naphthalene (1.73 mg, 13.5 µmol, 1.0 eq.) were dissolved in C_6D_6 (550 µL). After stirring the reaction mixture for 15 minutes at room temperature, the colour changed from black to dark blue. Quantitative conversion was observed by NMR spectroscopy. After the reaction the solvent was removed in *vacuo*, yielding essentially pure [(^{DIPeP}BDI)Yb]$_2$(η^4,η^4-naphthalene) (19.2 mg, 12.5 µmol, 93%) as a dark blue powder. Crystals suitable for single crystal X-ray diffraction, can be obtained by adding a few drops of THP to the concentrated solution of pentane at -35 °C leading to the formation of THP adduct **2**.

^{1}H NMR (C_6D_6, 600.13 MHz, 298 K): δ = 6.95-6.92 (m, 12H, C**H**-arom), 5.14 (s, 2H, C**H**-backbone), 3.03-3.00 (m, 8H, C**H**), 2.99-2.97 (m, 4H, C**H**-naphthalene), 1.91 (s, 12H, C**H**$_3$-backbone), 1.85-1.81 (m, 16H, C**H**$_2$), 1.66-1.61 (m, 16H, C**H**$_2$), 1.53-1.51 (m, 4H, C**H**-naphthalene), 1.11 (t, $^3J_{HH}$ = 6 Hz, 24H, C**H**$_3$), 0.90 (t, $^3J_{HH}$ = 6 Hz, 24H, C**H**$_3$) ppm.

^{13}C NMR (C_6D_6, 151 MHz, 298 K): δ = 165.1 (**C**N-backbone), 147.4 (**C**-arom), 139.1 (**C**-arom), 125.6 (**C**-arom), 123.2 (**C**-arom), 118.4 (**C**H-naphthalene), 95.2 (**C**H-backbone), 93.2 (**C**H-naphthalene) 42.0 (**C**H), 28.0 (**C**H$_2$), 25.2 (**C**H$_2$), 24.8 (**C**H$_3$-backbone), 12.5 (**C**H$_3$), 11.4 (**C**H$_3$) ppm.

FT-IR (ATR, pure): $\tilde{\nu}$ = 2956, 2927, 2868, 1516, 1450, 1424, 1394, 1340, 1273, 1216, 1185, 1165, 1138, 1095, 1049, 1015, 971, 921, 780, 760, 712, 656, 592, 462, 421.

Elemental analysis calculated for $C_{92}H_{142}N_4O_2Yb_2$ (M = 1682.28 g/mol): C 65.69, H 8.51, N 3.33 % with two molecules of coordinated diethyl ether. Found: C 65.09, H 8.44, N 3.17 %. Although the C value is likely too low due to metal carbide formation, it is provided to illustrate the best values obtained to date.

Synthesis of [(^{DIPeP}BDI)Yb(Et_2O)]$_2$(η^4,η^4-anthracene) (3)

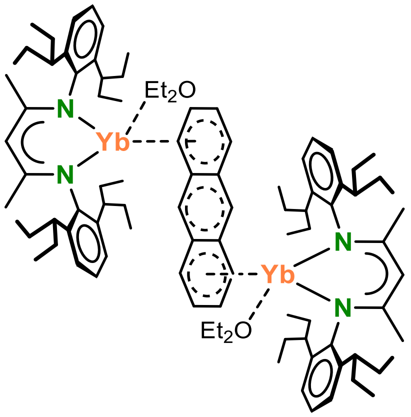

In a J-Young NMR tube, [(^{Dipep}BDI)Yb]$_2$(C_6H_6)] (20 mg, 13.5 µmol, 1.0 eq.) and anthracene (2.4 mg, 13.5 µmol, 1.0 eq.) were dissolved in C_6D_6 (550 µL). After stirring the reaction mixture for 15 minutes at room temperature, the colour changed from black to dark blue. Quantitative conversion was observed by NMR spectroscopy. After the reaction the solvent was removed in *vacuo*, yielding essentially pure [(^{DIPeP}BDI)Yb]$_2$(η^4,η^4-anthracene) (19.2 mg, 12.1 µmol, 90%) as dark blue powder. Crystals suitable for single crystal X-ray diffraction, were obtained by adding a few drops of diethyl ether to a concentrated pentane solution at -35 °C leading to the crystallization of Et_2O adduct **3**.

^{1}H NMR (C_6D_6, 600.13 MHz, 298 K): δ = 6.95 (s, 12H, C**H**-arom), 5.06 (s, 2H, C**H**-backbone), 4.24 (s, 4H, C**H**-anthracene), 3.29 (q, $^3J_{HH}$ = 6 Hz, 8H, C**H**$_2$-Et_2O), 3.01 (s, br, 4H, C**H**-anthracene), 2.93 (s, br, 8H, C**H**), 2.05 (s, br, 2H, C**H**- anthracene), 1.86 (s, 12H, C**H**$_3$-backbone), 1.83-1.77 (m, 16H, C**H**$_2$), 1.71-1.61 (m, 16H, C**H**$_2$), 1.12 (t, $^3J_{HH}$ = 6 Hz, 12H, C**H**$_3$-Et_2O), 1.05 (t, $^3J_{HH}$ = 6 Hz, 24H, C**H**$_3$), 0.88 (t, $^3J_{HH}$ = 6 Hz, 24H, C**H**$_3$) ppm.

^{13}C NMR (C_6D_6, 151 MHz, 298 K): δ = 165.4 (**C**N-backbone), 139.0 (**C**-arom), 125.8 (**C**-arom), 123.4 (**C**-arom), 117.7 (**C**H-anthracene), 102.4 (**C**H-anthracene), 95.1 (**C**H-backbone), 65.9 (**C**H$_2$-Et_2O) 42.0 (**C**H), 27.7 (**C**H$_2$), 25.0 (**C**H$_3$-backbone), 24.8 (**C**H$_2$), 15.6 (**C**H$_2$-Et_2O), 12.6 (**C**H$_3$), 11.1 (**C**H$_3$) ppm.

FT-IR (ATR, pure): $\tilde{\nu}$ = 2956, 2924, 2868, 1507, 1461, 1429, 1398, 1331, 1299, 1272, 1217, 1184, 1164, 1137, 1086, 1046, 1014, 921, 895, 817, 790, 761, 711, 677, 656, 590, 522, 493, 465, 422, 407.

Elemental analysis calculated for $C_{88}H_{124}N_4Yb_2$ (M = 1584.10 g/mol): C 66.72, H 7.89, N 3.54 %. Found: C 64.81, H 7.89, N 3.05 %. Although the C value is likely too low due to metal carbide formation, it is provided to illustrate the best values obtained to date.

Synthesis of $[(^{DIPeP}BDI)Yb]_2(\eta^8,\eta^8\text{-COT})$ (4)

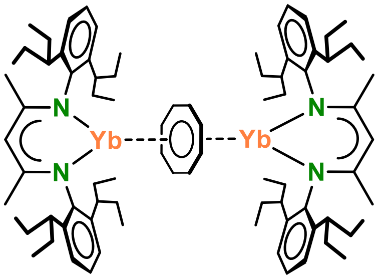

In a J-Young NMR tube, $[(^{Dipep}BDI)Yb]_2(C_6H_6)]$ (20 mg, 13.5 µmol, 1.0 eq.) and cyclooctatetraene (1.41 mg, 13.5 µmol, 1.0 eq.) were dissolved in C_6D_6 (550 µL). After stirring the reaction mixture for 15 minutes at room temperature, the colour changed from black to dark red. Quantitative conversion was observed by NMR spectroscopy. After the reaction the solvent was removed in *vacuo*, yielding essentially pure **4** (19.6 mg, 12.9 µmol, 96%) as a brown powder. Crystals suitable for single crystal X-ray diffraction, were obtained by storing a concentrated solution of **4** in pentane at -35 °C.

^{1}H NMR (C_6D_6, 600.13 MHz, 298 K): δ = 7.24-7.21 (m, 4H, C**H**-arom), 7.15 (s, br, 8H, C**H**-arom), 5.34 (s, 8H, C**H**-COT), 4.44 (s, 2H, C**H**-backbone), 2.42 (s, br, 8H, C**H**), 1.92-1.85 (m, 8H, C**H₂**), 1.81-1.75 (m, 8H, C**H₂**), 1.59-1.51 (m, 16H, C**H₂**), 1.45 (s, 12H, C**H**₃-backbone), 1.25 (t, $^3J_{HH}$ = 6 Hz, 24H, C**H**₃), 0.73 (t, $^3J_{HH}$ = 6 Hz, 24H, C**H**₃) ppm.

^{13}C NMR (C_6D_6, 151 MHz, 298 K): δ = 163.9 (**C**N-backbone), 147.7 (**C**-arom), 139.5 (**C**-arom), 125.6 (**C**-arom), 123.0 (**C**-arom), 94.2 (**C**H-backbone), 90.4 (**C**H-COT), 93.2 (**C**H-naphthalene) 41.4 (**C**H), 27.1 (**C**H_2), 24.4 (**C**H_3-backbone), 23.6 (**C**H_2), 12.3 (**C**H_3), 10.4 (**C**H_3) ppm.

FT-IR (ATR, pure): $\tilde{\nu}$ = 2955, 2929, 2868, 1542, 1520, 1449, 1397, 1976, 1342, 1304, 1268, 1212, 1165, 1137, 1107, 1091, 1070, 1020, 922, 891, 821, 785, 768, 751, 721, 654, 628, 418.

Elemental analysis calculated for $C_{82}H_{122}N_4Yb_2$ (M = 1510.01 g/mol): C 65.22, H 8.14, N 3.71 %. Found: C C 65.31, H 8.36, N 3.60 %.

Synthesis of (^{DIPeP}BDI)Sm(η^8-COT)(THF) (5)

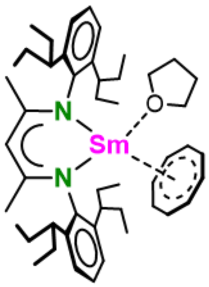

In a J-Young NMR tube, [(^{Dipep}BDI)Sm]$_2$(C_6H_6) (25 mg, 17.4 µmol, 1.0 eq.) and cyclooctatetraene (3.62 mg, 34.8 µmol, 2.0 eq.) were dissolved in C_6D_6 (550 µL). After stirring the reaction mixture for 15 minutes at room temperature the colour changed from black to dark red. The progress of the reaction was monitored by NMR spectroscopy. After the reaction the solvent was removed in *vacuo* and a red brown solid was obtained. Crystals suitable for single crystal X-ray diffraction were grown at −35 °C by adding a few drops of THF to a concentrated pentane solution (15.5 mg, 26.4 µmol, 76%) leading to the formation of THF adduct **5**.

^{1}H NMR (C_6D_6, 600.13 MHz, 298 K): δ = 8.26 (s, 8H, C**H**-COT), 7.99 (t, $^3J_{HH}$ = 6 Hz, 2H, C**H**-arom), 7.64 (s, 1H, C**H**-backbone), 6.51 (d, $^3J_{HH}$ = 12 Hz, 4H, C**H**-arom), 3.54-3.52 (m, 10H, C**H**$_3$-backbone and α-C**H**$_2$, THF), 1.71-1.62 m, 4H, C**H**$_2$), 1.41-1.39 (m, 3H, β-C**H**$_2$, THF), 0.70 (t, $^3J_{HH}$ = 6 Hz, 12H, C**H**$_3$), -1.38 (t, $^3J_{HH}$ = 6 Hz, 12H, C**H**$_3$), -1.67 – (-1.75) (m, 4H, C**H**$_2$), -1.90 – (-1.96) (m, 4H, C**H**$_2$) -2.03 – (-2.05) (m, 4H, C**H**$_2$)ppm.

^{13}C NMR (C_6D_6, 151 MHz, 298 K): δ = 171.6 (**C**N-backbone), 155.0 (**C**-arom), 142.1 (**C**-arom), 128.4 (**C**-arom), 125.3 (**C**-arom), 99.1 (**C**H-backbone), 84.9 (CH, COT), 67.9 (α-C**H**$_2$, THF), 43.4 (**C**H), 26.1 (**C**H$_2$), 25.8 (β-C**H**$_2$, THF) 23.5 (**C**H$_2$), 20.8 (**C**H$_3$-backbone), 10.3 (**C**H$_3$), 9.9 (**C**H$_3$), ppm.

FT-IR (ATR, pure): $\tilde{\nu}$ = 2955, 2926, 2868, 1515, 1459, 1424, 1393, 1357, 1338, 1264, 1216, 1162, 1138, 1106, 1096, 1011, 918, 896, 857, 832, 791, 770, 751, 700, 667, 632, 522, 500, 429.

Elemental analysis calculated for $C_{49}H_{73}N_2OSm$ (M = 856.50 g/mol): C, 68.71; H, 8.59; N, 3.27 % with one molecule of coordinated THF. Found: C 67.25, H 8.36, N 3.27 %. Although the C value is likely too low due to metal carbide formation, it is provided to illustrate the best values obtained to date.

Synthesis of [(^{DIPeP}BDI)Sm(2-Me-THF)]$_2$(η^6,η^6-anthracene) (6)

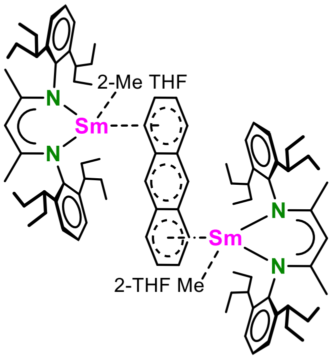

In a J-Young NMR tube, [(^{Dipep}BDI)Sm]$_2$(C_6H_6) (120 mg, 0.083 mmol, 1.0 eq.) and anthracene (14.9 mg, 0.083 mmol, 1.0 eq.) were dissolved in C_6D_6 (550 µL). After stirring the reaction mixture for 1.5 h at room temperature the colour changed from black to dark blue. The progress of the reaction was monitored by NMR spectroscopy. After the reaction starting material consumed the solvent was removed in *vacuo* and a dark solid was obtained. Crystals suitable for single crystal X-ray diffraction, were grown at -35 °C by adding a few drops of 2-Me-THF to a concentrated pentane solution leading to the formation of 2-Me THF adduct **6** (84 mg, 0.049 mmol, 59%).

^{1}H NMR (C_6D_6, 600.13 MHz, 298 K): δ = 26.09, 22.20, 21.75, 13.43, 6.74, 4.31, 4.20, 3.79, 3.63, 3.45, 3.16, 2.11, 1.90, 1.69, 1.49, 1.46, 1.41, 1.31, 1.26, 1.20, 1.00, 0.88, 0.75, 0.49, -0.85, -11.60, -14.89 ppm. Due to paramagnetic nature of the complex, the assignment of NMR signals was not possible.

FT-IR (ATR, pure): $\tilde{\nu}$ =2955, 2928, 2867, 1549, 1508, 1430, 1398, 1375, 1340, 1327, 1310, 1278, 1246, 1215, 1183, 1162, 1135, 1089, 1014, 921, 895, 821, 813, 789, 757, 727, 707, 471, 424, 420.

Elemental analysis calculated for $C_{88}H_{124}N_4Sm_2$ (M = 1538.71 g/mol): C 68.69, H 8.12, N 3.64 %. Found: C 67.63, H 8.65, N 3.10 %. Although the C value is likely too low due to metal carbide formation, it is provided to illustrate the best values obtained to date.

Synthesis of [(^{DIPeP}BDI)Sm(THF)]$_2$(η^6,η^6-toluene) (7)

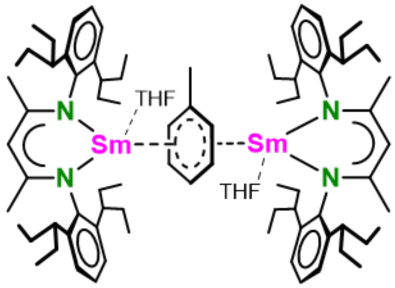

[(^{DIPeP}BDI)SmI]$_2$ (0.100 g, 0.062 mmol) and KC_8 (0.084 g, 0.62 mmol) were suspended in toluene (6 mL) and vigorously stirred at room temperature for 1.5 hours. The resulting black coloured suspension was filtered and the solvent was removed *in vacuo.* The solid residue was stripped with pentane (0.5 mL) and dried completely to afford an essentially pure black powder (0.084 g, 0.058 mmol, 94%). Crystals suitable for single crystal X-ray diffraction, were grown at -35 °C by adding a few drops of THF to the concentrated solution of pentane leading to the formation of THF adduct **7**.

^{1}H NMR (C_6D_6, 600.13 MHz, 298 K): δ = 22.73 (s, 2H, C_7**H_8**-aromatic), 21.57 (s, 1H, C_7**H_8**-aromatic), 21.47 (s, 2H, C_7**H_8**-aromatic), 10.55 (s, 2H, C**H**-backbone), 6.64 (t, $^3J_{HH}$ = 6 Hz, 4H, C**H**-arom), 6.32(d, $^3J_{HH}$ = 6 Hz, 8H, C**H**-arom), 5.21 (s, 12H, C**H**$_3$-backbone), 2.17-2.12 (m, 8H, C**H**$_2$), 1.32-1.24 (m, 8H, C**H**$_2$), 1.01 (t, $^3J_{HH}$ = 6 Hz, 24H, C**H**$_3$), -0.21 (s, br, 8H, C**H**), -1.58 (t, $^3J_{HH}$ = 6 Hz, 24H, C**H**$_3$), -3.21-(-3.26) (m, 8H, C**H**$_2$), -3.44-(-3.49) (m, 8H, C**H**$_2$), -12.34 (s, 3H, CH_3-toluene) ppm.

^{13}C NMR (C_6D_6, 151 MHz, 298 K): δ = 176.2 (**C**N-backbone), 152.8 (**C**-arom), 139.3 (**C**-arom), 124.8 (**C**-arom), 122.9 (**C**-arom), 99.5 (**C**H-backbone), 41.5 (**C**H), 24.4 (**C**H$_2$), 24.0 (**C**H$_2$), 18.1 (**C**H$_3$-backbone), 10.9 (**C**H$_3$), 9.1 (**C**H$_3$), ppm. ^{13}C signals corresponding to bridged toluene are not observed.

FT-IR (ATR, pure): $\tilde{\nu}$ = 2956, 2926, 2868, 1546, 1521, 1451, 1396, 1376, 1342, 1259, 1212, 1163, 1139, 1095, 1017, 924, 897, 785, 692, 661, 628, 422.

Elemental analysis calculated for $C_{81}H_{122}N_4Sm_2$ (M = 1452.62 g/mol): C 66.98, H 8.47, N 3.86 %. Found: C 66.59, H 8.41, N 3.96 %.

Synthesis of (^{DIPeP}BDI)Sm(η^6,η^6-biphenyl) Sm(^{DIPeP}BDI)(THF) (8)

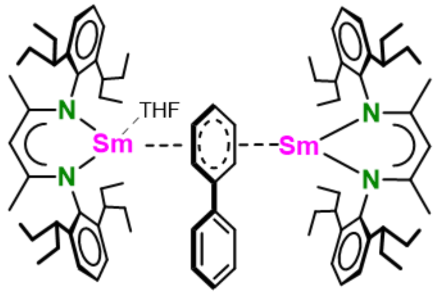

[(^{DIPeP}BDI)SmI]$_2$ (0.100 g, 0.062 mmol) and biphenyl (9.5 mg, 0.062 mmol) were suspended in hexane (6 mL) and KC_8 (0.084 g, 0.62 mmol) was added to the reaction mixture. The mixture was vigorously stirred at room temperature for 6 hours. The resulting black coloured suspension was filtered and the solvent was removed *in vacuo.* The solid residue was stripped with pentane (0.5 mL) and dried completely to afford an essentially pure fine black powder [(^{DIPeP}BDI)Sm]$_2$(η^6,η^6-biphenyl) (0.085 g, 0.056 mmol, 91%). Crystals suitable for single crystal X-ray diffraction, were grown at -35 °C by adding a few drops of THF to the concentrated solution of pentane.

^{1}H NMR (C_6D_6, 600.13 MHz, 298 K): δ = 10.91 (s, 2H, C**H**-backbone), 6.63 (t, $^3J_{HH}$ = 6 Hz, 4H, C**H**-arom), 6.28 (d, $^3J_{HH}$ = 6 Hz, 8H, C**H**-arom), 5.43 (s, 12H, C**H**$_3$-backbone), 2.13 (s, br, 8H, C**H**$_2$), 0.98-0.95 (m, 24H, C**H**$_3$), 0.75-0.71 (m, 8H, C**H**$_2$), -0.59 (s, br, 8H, C**H**), -1.82 (s, br, 24H, C**H**$_3$), -3.16 (s, br, 8H, C**H**$_2$), -4.91 (s, br, 8H, C**H**$_2$) ppm.

^{13}C NMR (C_6D_6, 151 MHz, 298 K): δ = 176.9 (**C**N-backbone), 153.5 (**C**-arom), 139.2 (**C**-arom), 125.5 (**C**-arom), 122.9 (**C**-arom), 100.1 (**C**H-backbone), 42.6 (**C**H), 24.8 (**C**H$_2$), 23.7 (**C**H$_2$), 17.6 (**C**H$_3$-backbone), 10.8 (**C**H$_3$), 9.6 (**C**H$_3$), ppm. ^{13}C signals corresponding to bridged biphenyl are not observed.

FT-IR (ATR, pure): $\tilde{\nu}$ = 2956, 2929, 2868, 1583, 1544, 1523, 1494, 1450, 1424, 1392, 1343, 1266, 1253, 1214, 1188, 1164, 1138, 1094, 1020, 990, 923, 897, 786, 771, 743, 686, 653, 629, 418.

Elemental analysis calculated for $C_{94}H_{140}N_4O_2Sm_2$ (M = 1658.90 g/mol): C 68.06, H 8.51, N 3.38 %. Found: C 68.00, H 8.26, N 3.93 %.

Synthesis of [(^{DIPeP}BDI)Sm]$_2$(η^6,η^6-1,3,5-triphenyl-benzene) (9)

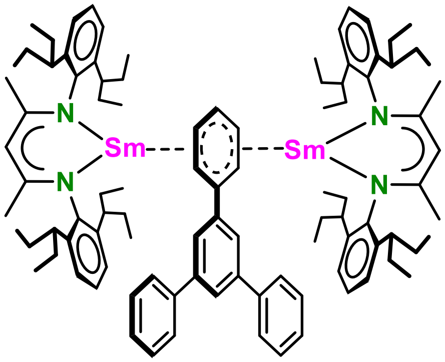

[(^{DIPeP}BDI)SmI]$_2$ (0.050 g, 0.031 mmol) and 1,3,5-triphenyl-benzene (9.5 mg, 0.031 mmol) were suspended in hexane (6.0 mL) and KC_8 (0.042 g, 0.309 mmol) was added to the reaction mixture. The mixture was vigorously stirred at room temperature for 5 hours. The resulting black coloured suspension was filtered and the solvent was removed *in vacuo.* The solid residue was stripped with pentane (0.5 mL) and dried completely to afford an essentially pure fine black powder **9** (0.049 g, 29.4 µmol, 95%). Subsequently, the solid was dissolved in pentane (0.5 mL), filtered and slowly cooled to -35 °C. Dark brown black crystals suitable for X-ray diffraction analysis were grown overnight.

^{1}H NMR (C_6D_6, 600.13 MHz, 298 K): δ = 28.36 (s, 2H, 1,3,5-triphenyl benzene), 21.88 (s, 2H, 1,3,5-triphenyl benzene), 14.15 (s, 1H, C**H**-1,3,5-triphenyl benzene), 11.05 (s, 2H, C**H**-backbone), 9.23 (s, 1H, C**H**-1,3,5-triphenyl benzene), 8.05 (s, 2H, C**H**-1,3,5-triphenyl benzene), 6.86 (t, $^3J_{HH}$ = 6 Hz, 4H, C**H**-arom), 6.73 (t, $^3J_{HH}$ = 6 Hz, 2H, C**H**-arom), 6.40 (d, $^3J_{HH}$ = 6 Hz, 8H, C**H**-arom), 6.17 (t, $^3J_{HH}$ = 6 Hz, 4H, C**H**-arom), 6.00 (d, $^3J_{HH}$ = 6 Hz, 8H, C**H**-arom), 5.46 (s, 12H, C**H**$_3$-backbone), 2.18-2.11 (m, 8H, C**H**$_2$), 0.97-0.94 (m, 24H, C**H**$_3$), 0.84-0.78 (m, 8H, C**H**$_2$), -0.58 (s, br, 8H, C**H**), -1.68 (t, $^3J_{HH}$ = 6 Hz, 24H, C**H**$_3$), -3.18 (s, br, 8H, C**H**$_2$), -4.98 (s, br, 8H, C**H**$_2$) ppm.

^{13}C NMR (C_6D_6, 151 MHz, 298 K): δ = 177.1 (**C**N-backbone), 154.4 (**C**H-1,3,5-triphenyl benzene), 152.6 (**C**-arom), 141.4 (**C**H-1,3,5-triphenyl benzene), 138.8 (**C**-arom), 127.7 (**C**H-1,3,5-triphenyl benzene), 127.4 (**C**H-1,3,5-triphenyl benzene), 125.3 (**C**-arom), 122.6 (**C**-arom), 100.3 (**C**H-backbone), 98.6 (**C**H-1,3,5-triphenyl benzene), 91.5 (**C**H-1,3,5-triphenyl benzene) 42.7 (**C**H), 25.1 (**C**H$_2$), 23.8 (**C**H$_2$), 17.4 (**C**H$_3$-backbone), 10.8 (**C**H$_3$), 9.8 (**C**H$_3$), ppm.

FT-IR (ATR, pure): $\tilde{\nu}$ = 2956, 2927, 2868, 1545, 1523, 1492, 1456, 1423, 1393, 1376, 1343, 1266, 1225, 1166, 1095, 1020, 925, 907, 788, 775, 750, 686, 682, 653, 612, 417.

Elemental analysis calculated for $C_{98}H_{132}N_4Sm_2$ (M = 1666.88 g/mol): C 70.62, H 7.98, N 3.36 %. Found: C 69.72, H 7.95, N 3.40 %. Although the C value is likely too low due to metal carbide formation, it is provided to illustrate the best values obtained to date.

Synthesis of [(^{DIPeP}BDI)Sm(THF)]$_2$(η^6,η^6-pyrene) (10)

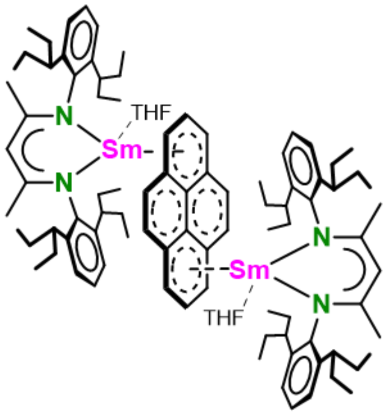

[(^{DIPeP}BDI)SmI]$_2$ (0.100 g, 0.062 mmol) and pyrene (12.5 mg, , 0.062) were suspended in hexane (6 mL) and KC_8 (0.084 g, 619 µmol) was added to the reaction mixture. The mixture was vigorously stirred at room temperature for 2 hours. The resulting blue coloured suspension was filtered and the solvent was removed *in vacuo.* The solid residue was stripped with pentane (0.5 mL) and dried completely to afford a fine black powder. Crystals suitable for single crystal X-ray diffraction, were grown at −35 °C by adding a few drops of THF to a concentrated pentane solution (0.065 g, 0.0384 mmol, 62%).

^{1}H NMR (C_6D_6, 600.13 MHz, 298 K): δ = 27.51, 24.11, 23.46, 15.45, 12.72, 4.23, 3.24, 3.14, 1.77, 1.41, 0.89, 0.71, -0.13, -6.48, -9.85 ppm. Due to paramagnetic nature of the complex, the assignment of NMR signals was not possible.

FT-IR (ATR, pure): $\tilde{\nu}$ = 2955, 2926, 2868, 1528, 1444, 1423, 1396, 1341, 1275, 1232, 1162, 1138, 1094, 1019, 959, 920, 863, 787, 746, 698, 665, 472, 425.

Elemental analysis calculated for $C_{98}H_{140}N_4O_2Sm_2$ (M = 1706.94 g/mol): C 68.96, H 8.27, N 3.28. Found: C 68.14, H 7.92, N 3.83 %. Two THF coordinated to Sm have been included for calculation. Although the C value is likely too low due to metal carbide formation, it is provided to illustrate the best values obtained to date.

3. Spectroscopic data

3a. NMR data

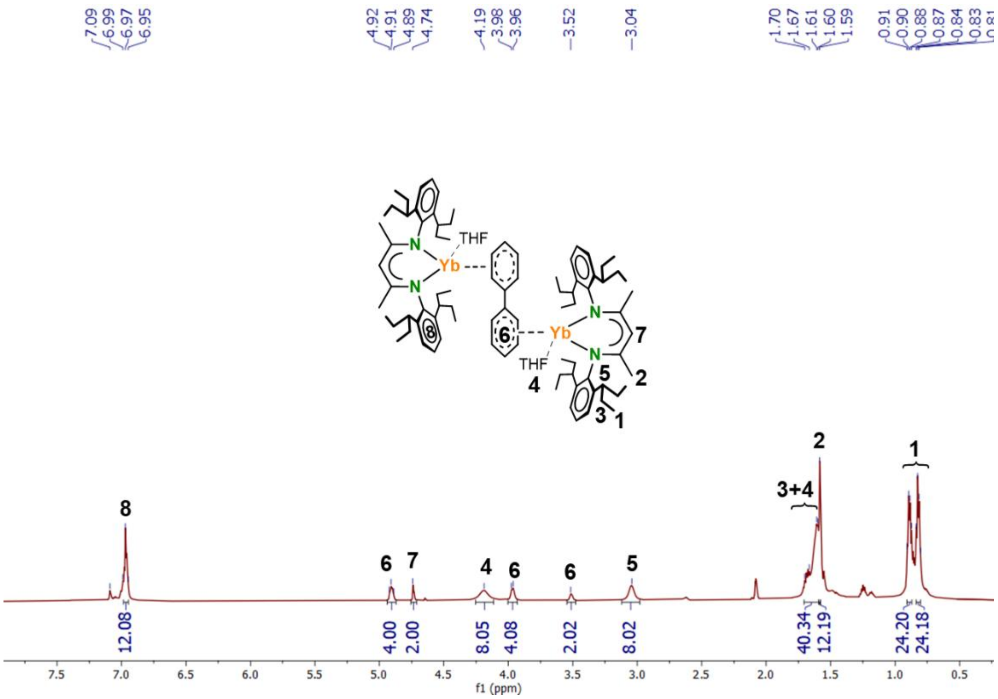

Figure S1. ^{1}H NMR (600.13 MHz, 298 K, C_7D_8) of [(^{DIPeP}BDI)Yb(THF)]$_2$(η^6,η^6-biphenyl) (**1**).

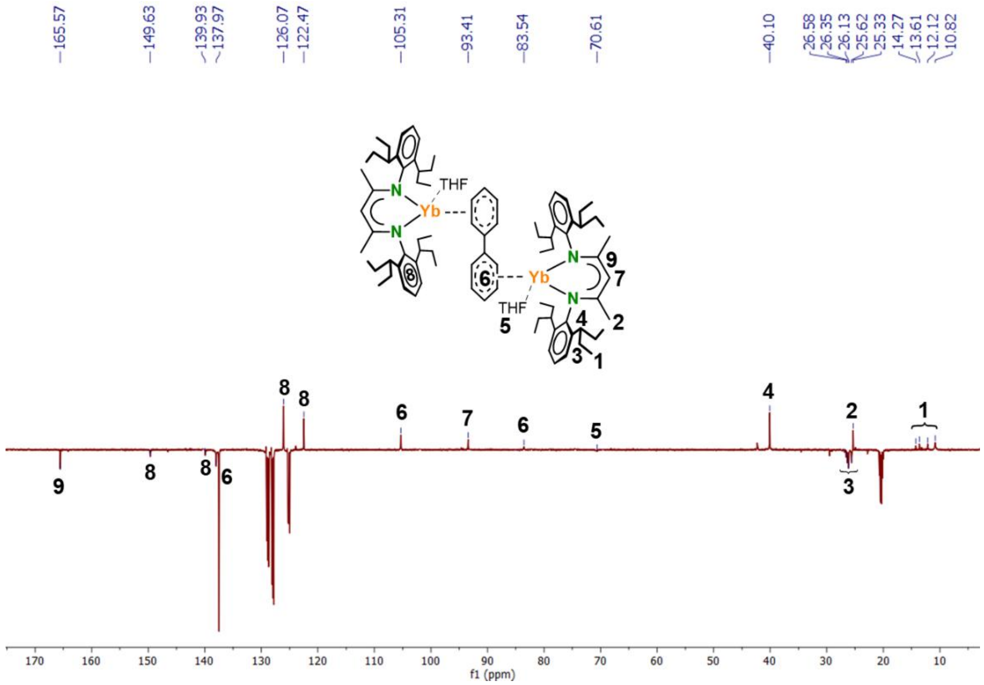

Figure S2. ^{13}C-APT NMR (150.92 MHz, 298 K, C_7D_8) of [(^{DIPeP}BDI)Yb(THF)]$_2$(η^6,η^6-biphenyl) (**1**).

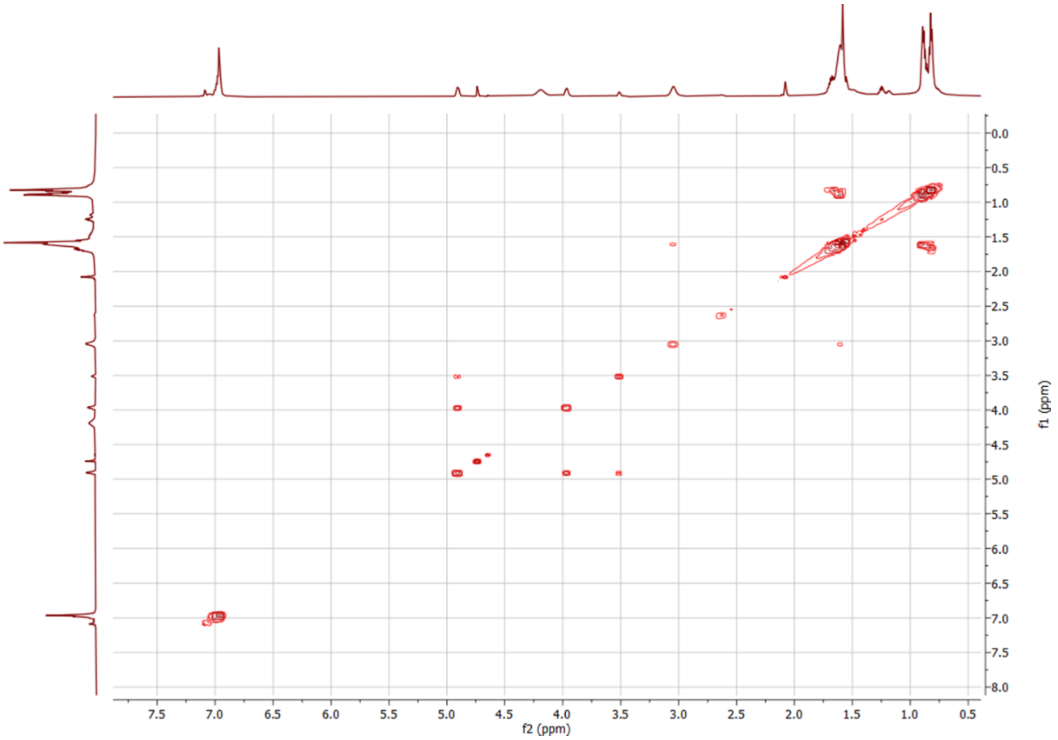

Figure S3. ^{1}H-^{1}H COSY NMR (600.13 MHz, 298 K, C_7D_8) of [(^{DIPeP}BDI)Yb(THF)]$_2$(η^6,η^6-biphenyl) (**1**).

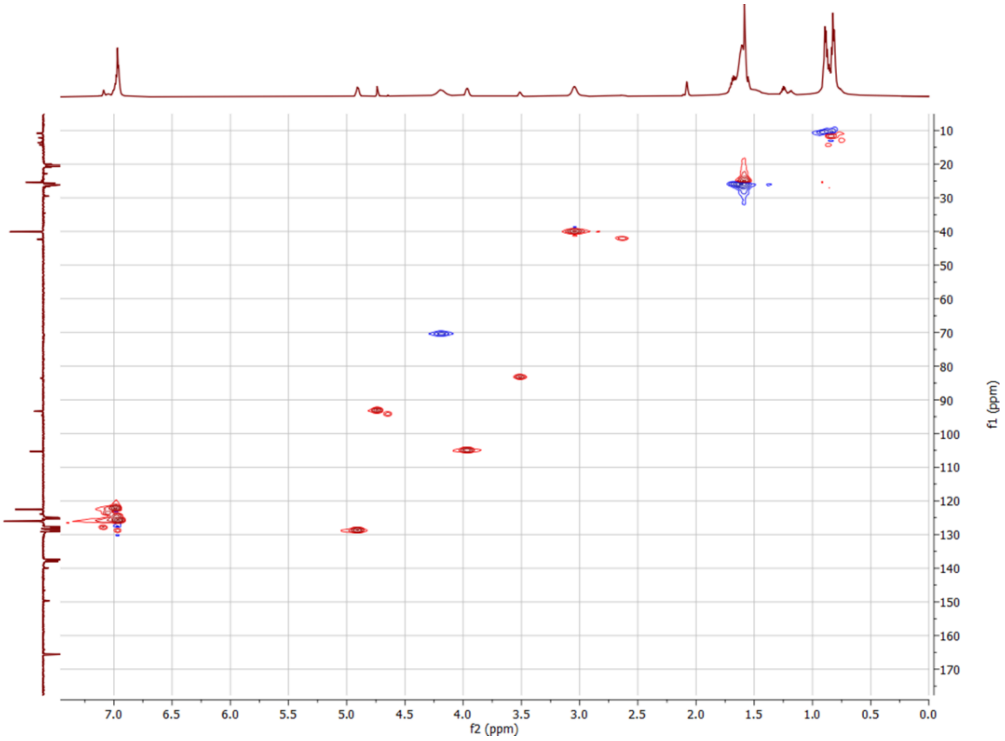

Figure S4. ^{1}H-^{13}C HSQC NMR (600.13/150.92 MHz, 298 K, C_7D_8) [(^{DIPeP}BDI)Yb(THF)]$_2$(η^6,η^6-biphenyl) (**1**).

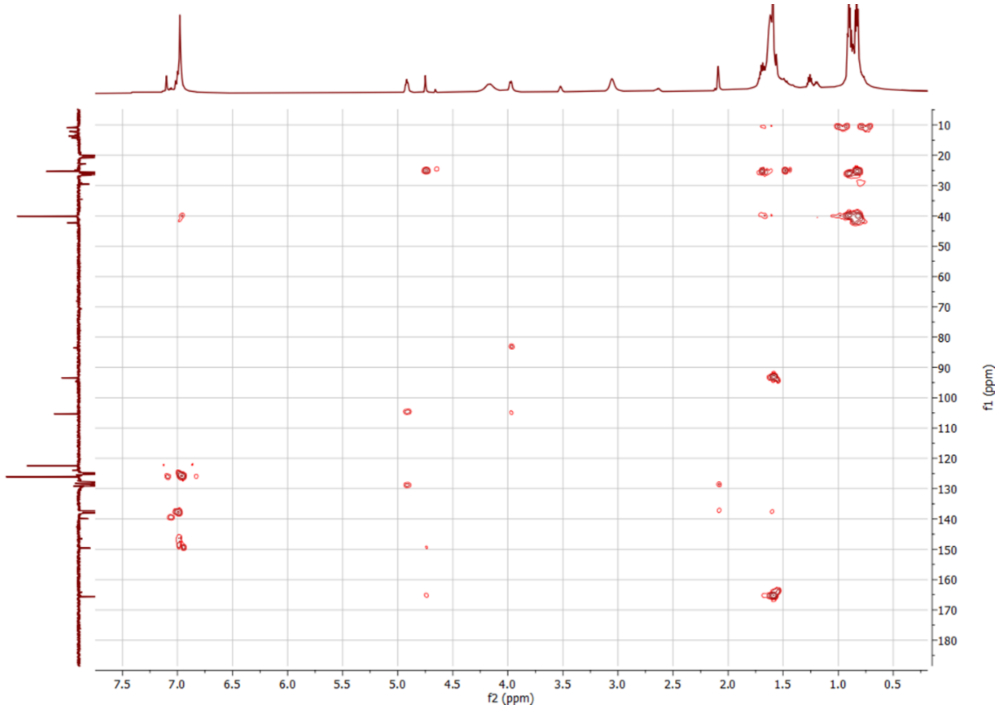

Figure S5. ^{1}H-^{13}C HMBC NMR (600.13/150.92 MHz, 298 K, C_7D_8) of [(^{DIPeP}BDI)Yb(THF)]$_2$(η^6,η^6-biphenyl)(**1**).

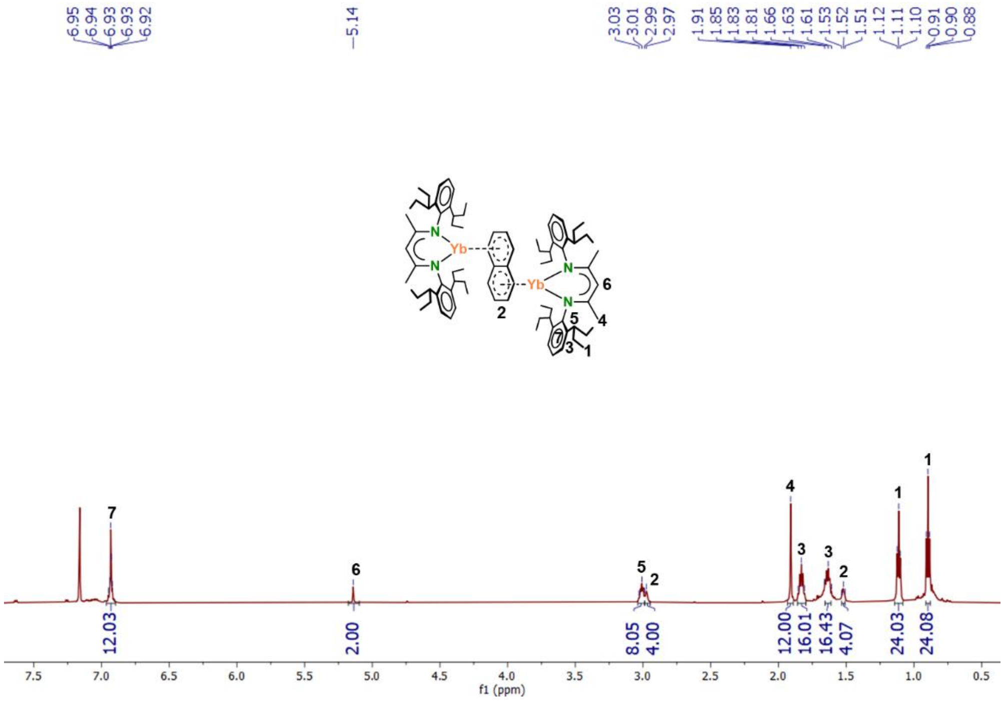

Figure S6. ^{1}H NMR (600.13 MHz, 298 K, C_6D_6) of solvent free [(^{DIPeP}BDI)Yb]$_2$(η^4,η^4-naphthalene) (**2**).

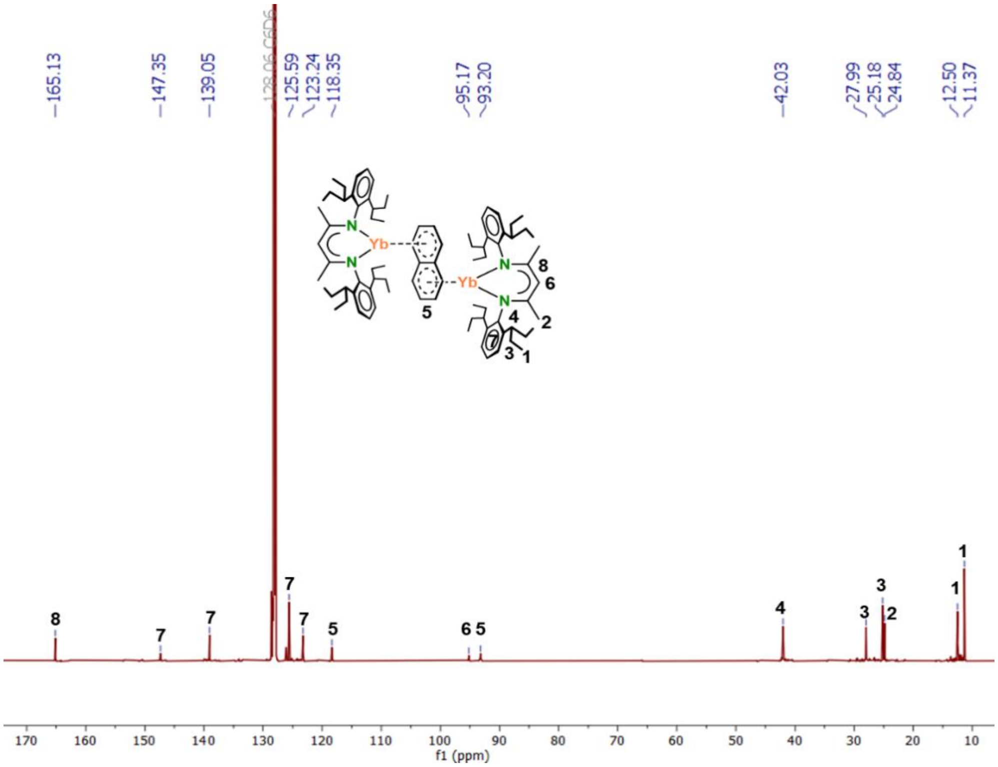

Figure S7. $^{13}C\{^{1}H\}$ NMR (150.92 MHz, 298 K, C_6D_6) of solvent free $[(^{DIPeP}BDI)Yb]_2(\eta^4,\eta^4$-naphthalene)(**2**).

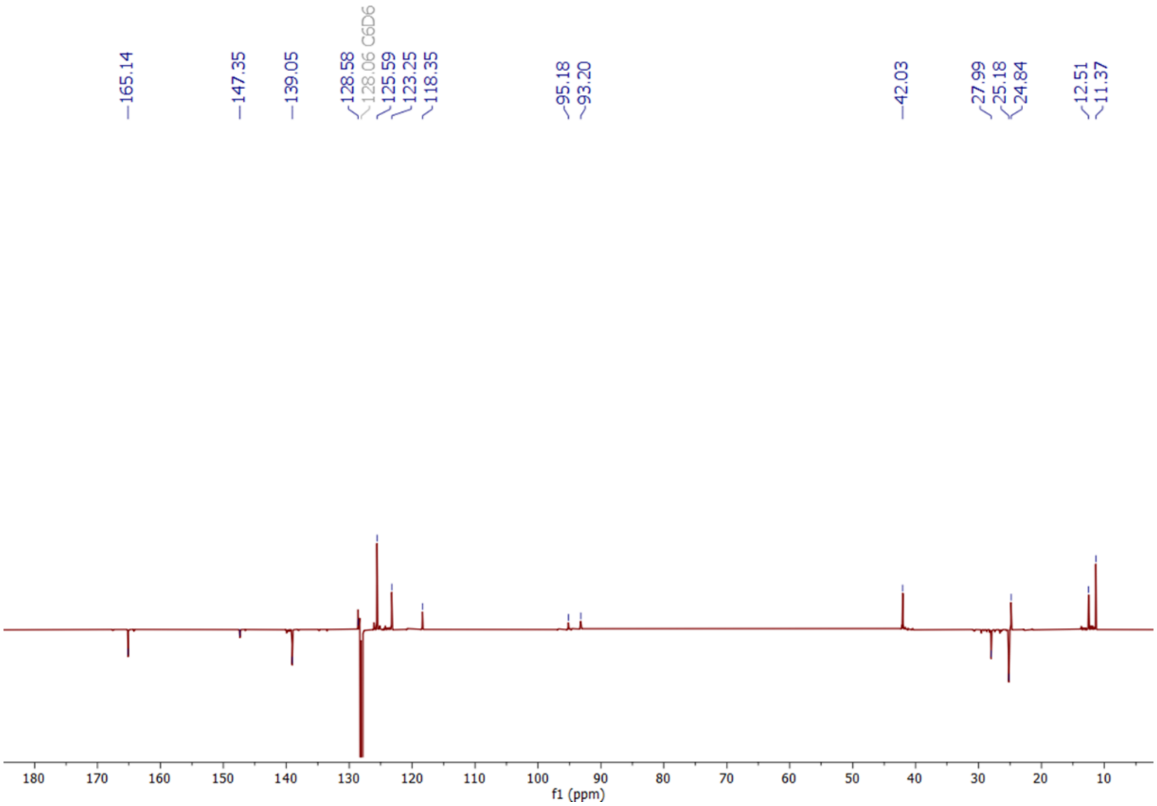

Figure S8. ^{13}C-APT NMR (150.92 MHz, 298 K, C_6D_6) of solvent free $[(^{DIPeP}BDI)Yb]_2(\eta^4,\eta^4$-naphthalene)(**2**).

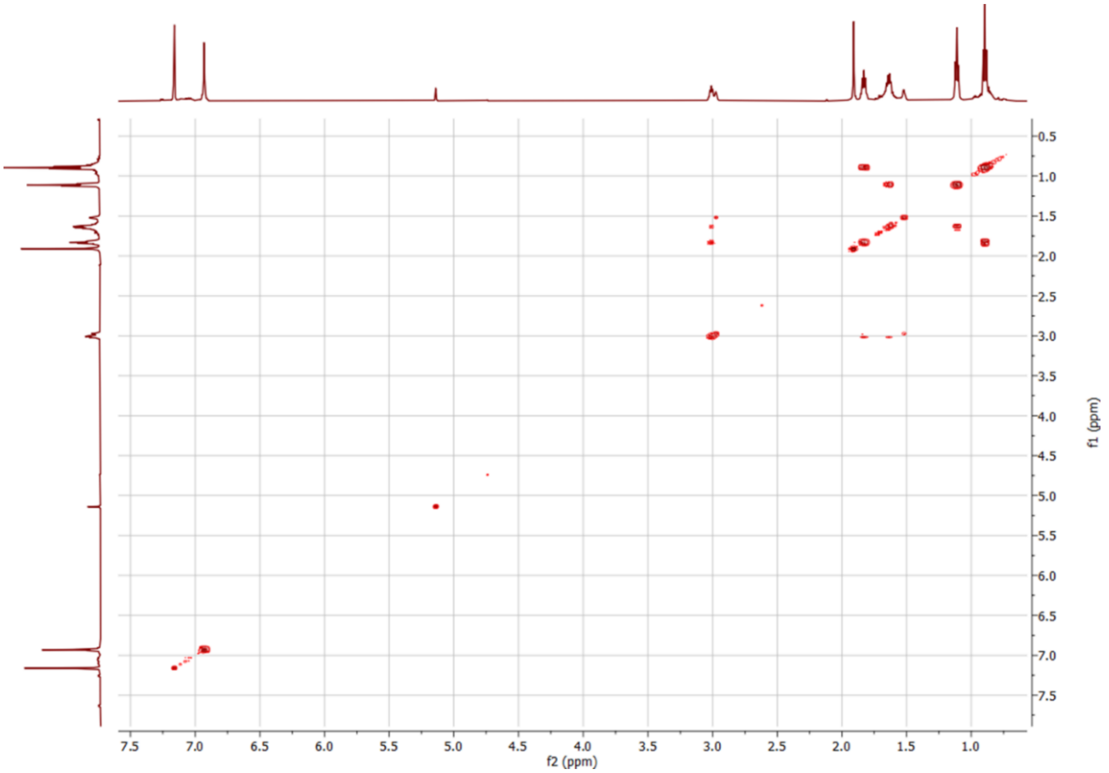

Figure S9. ^{1}H-^{1}H COSY NMR (600.13 MHz, 298 K, C_6D_6) of solvent free [(^{DIPeP}BDI)Yb]$_2$(η^4,η^4-naphthalene) (**2**).

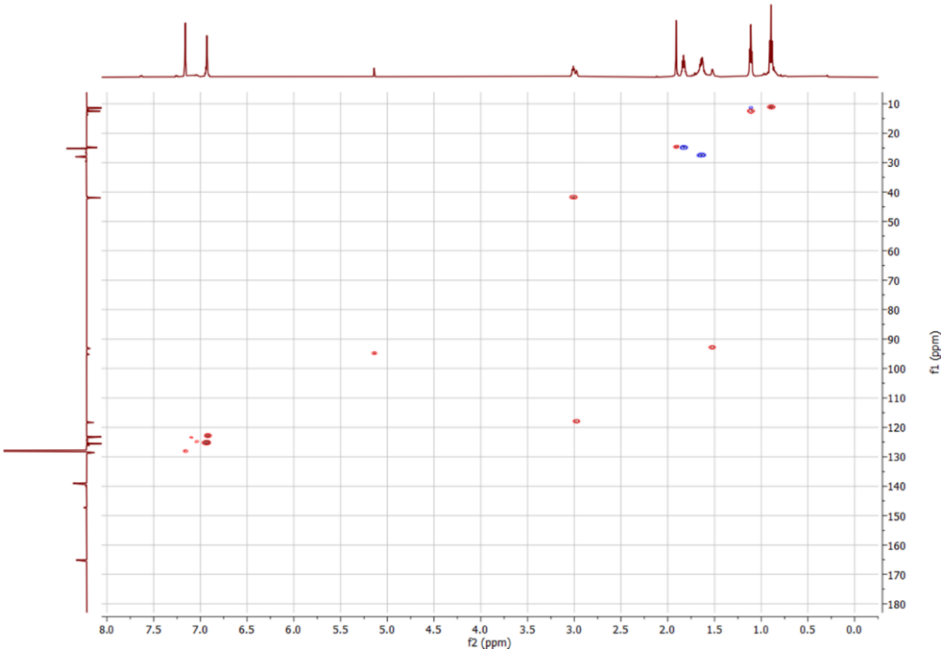

Figure S10. ^{1}H-^{13}C HSQC NMR (600.13/150.92 MHz, 298 K, C_6D_6) of solvent free [(^{DIPeP}BDI)Yb]$_2$(η^4,η^4-naphthalene) (**2**).

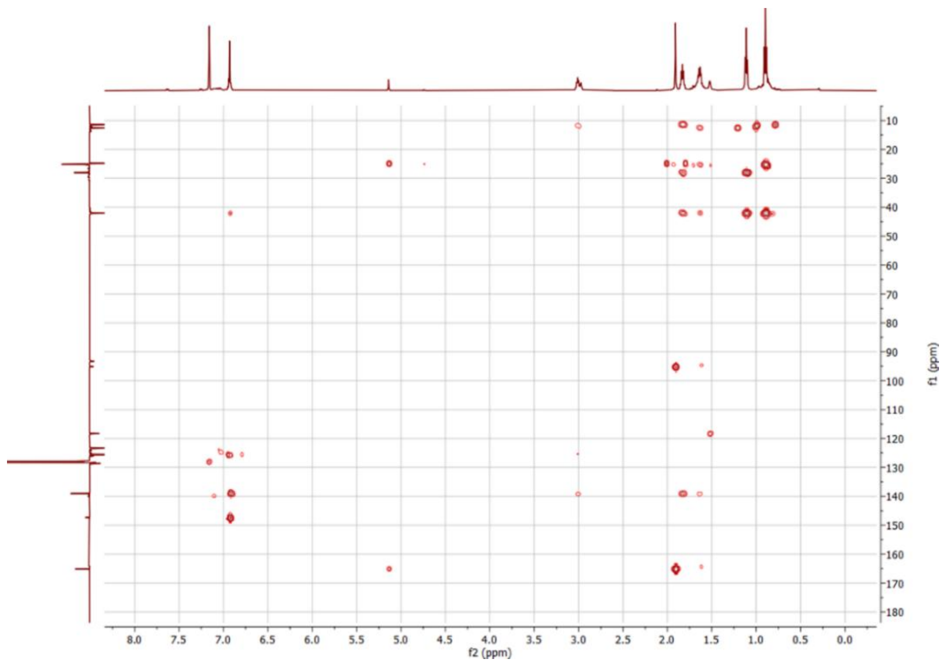

Figure S11. ^{1}H-^{13}C HMBC NMR (600.13/150.92 MHz, 298 K, C_6D_6) of solvent free [(^{DIPeP}BDI)Yb]$_2$(η^4,η^4-naphthalene) (**2**).

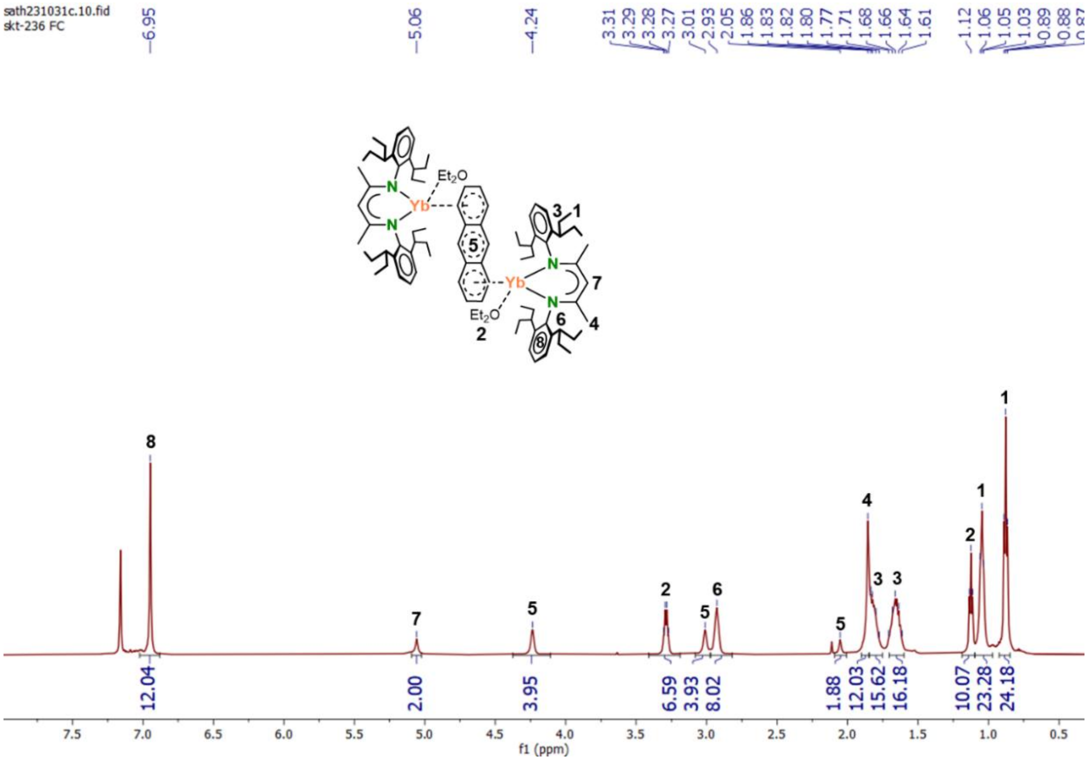

Figure S12. ^{1}H NMR (600.13 MHz, 298 K, C_6D_6) of [(^{DIPeP}BDI)Yb(Et_2O)]$_2$(η^4,η^4-anthracene) (**3**).

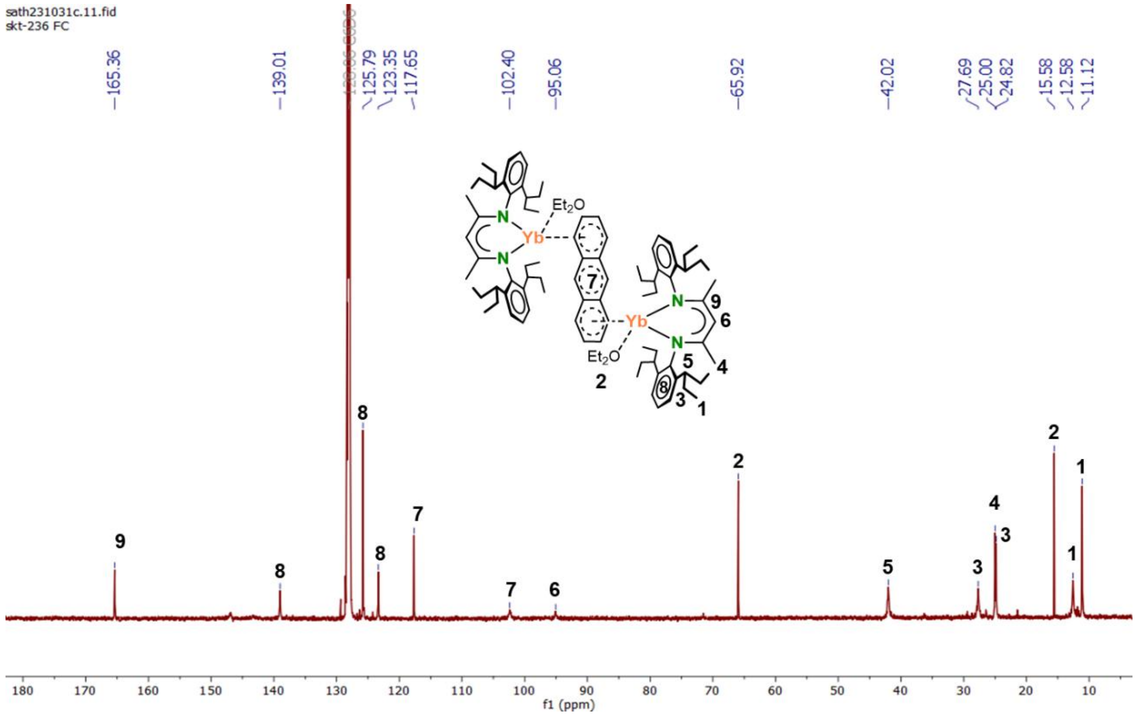

Figure S13. $^{13}C\{^{1}H\}$ NMR (150.92 MHz, 298 K, C_6D_6) of $[(^{DIPeP}BDI)Yb(Et_2O)]_2(\eta^4,\eta^4$-anthracene) (**3**).

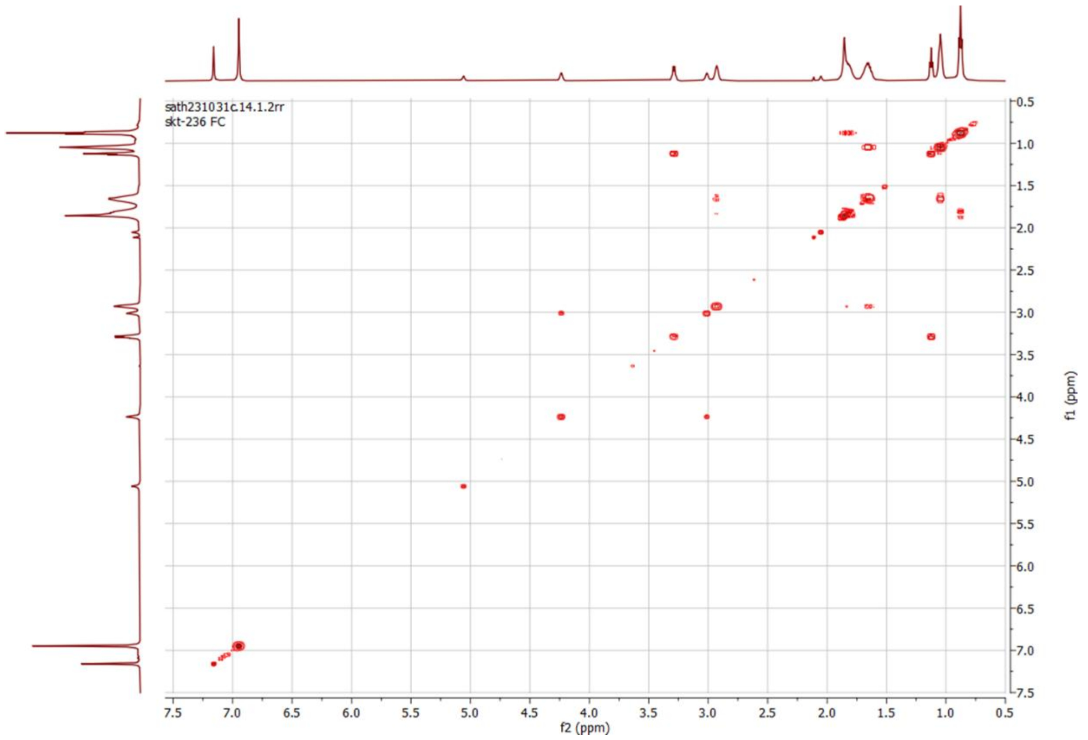

Figure S14. ^{1}H-^{1}H COSY NMR (600.13 MHz, 298 K, C_6D_6) of $[(^{DIPeP}BDI)Yb(Et_2O)]_2(\eta^4,\eta^4$-anthracene) (**3**).

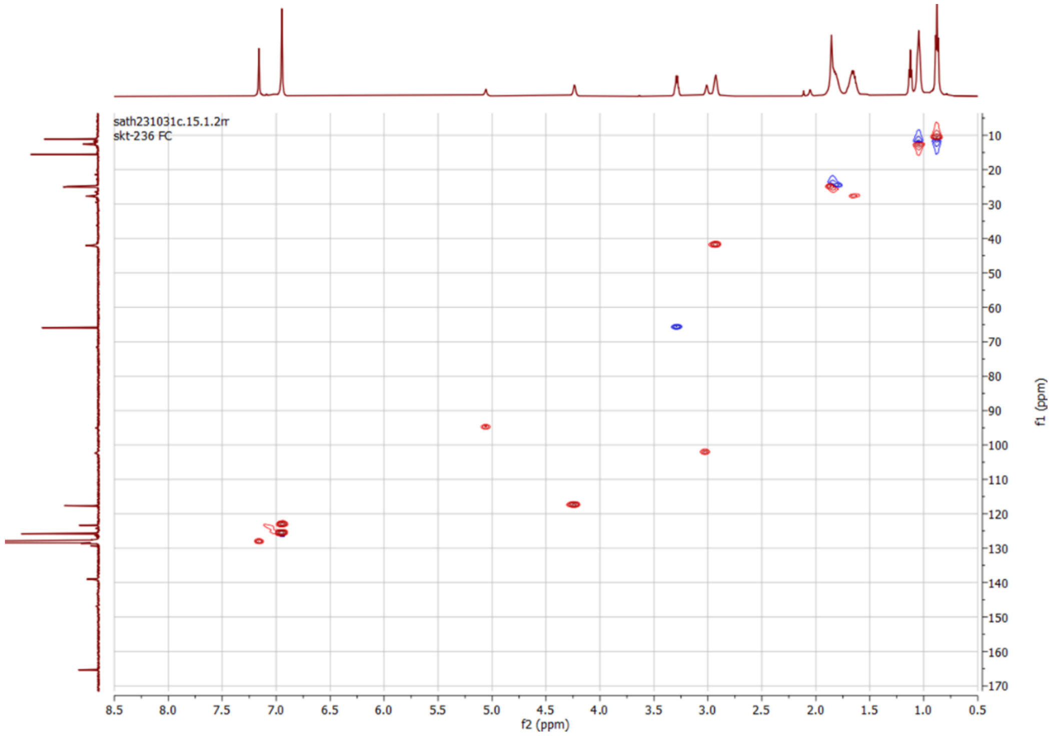

Figure S15. ^{1}H-^{13}C HSQC NMR (600.13/150.92 MHz, 298 K, C_6D_6) of $[(^{DIPeP}BDI)Yb(Et_2O)]_2(\eta^4,\eta^4$-anthracene) (**3**).

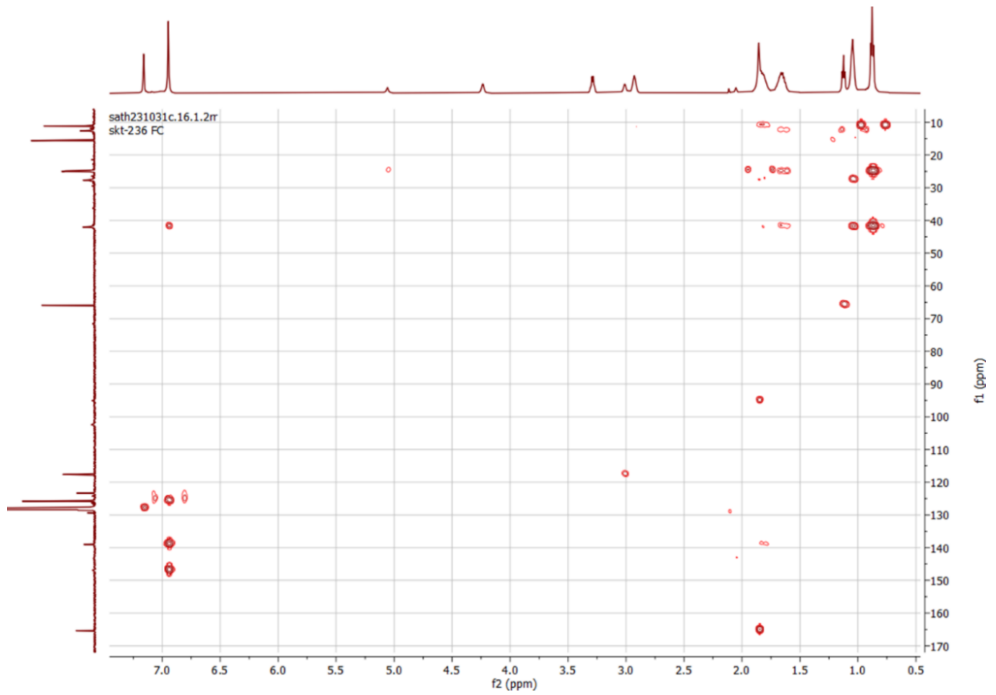

Figure S16. ^{1}H-^{13}C HMBC NMR (600.13/150.92 MHz, 298 K, C_6D_6) of $[(^{DIPeP}BDI)Yb(Et_2O)]_2(\eta^4,\eta^4$-anthracene) (**3**).

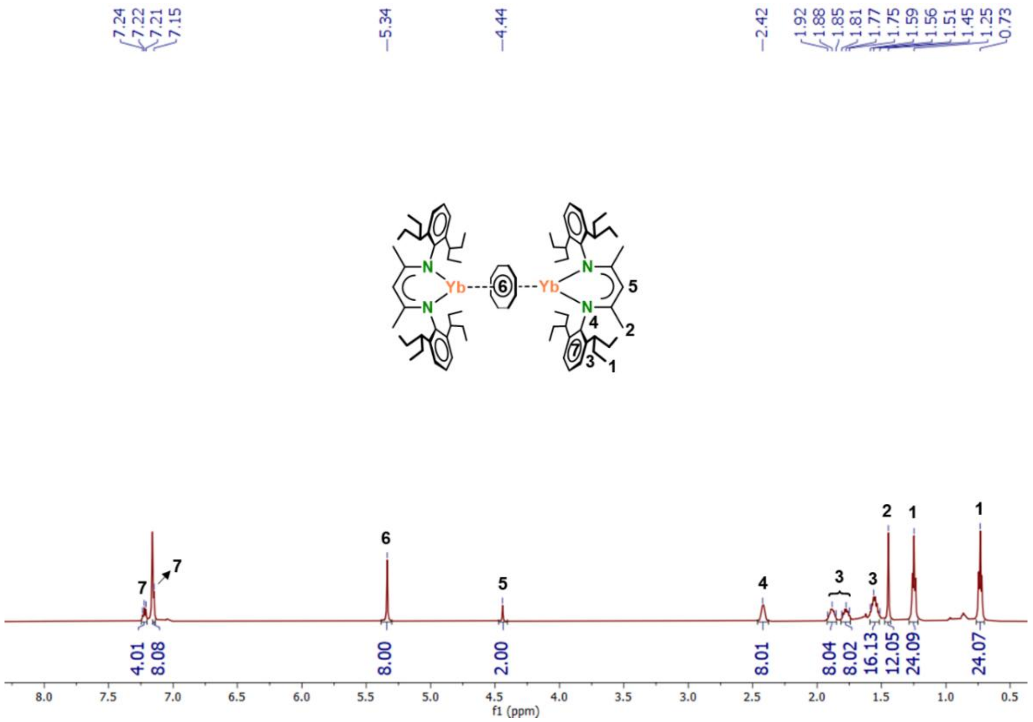

Figure S17. ^{1}H NMR (600.13 MHz, 298 K, C_6D_6) of [(^{DIPeP}BDI)Yb]$_2$(η^8,η^8-COT) (**4**).

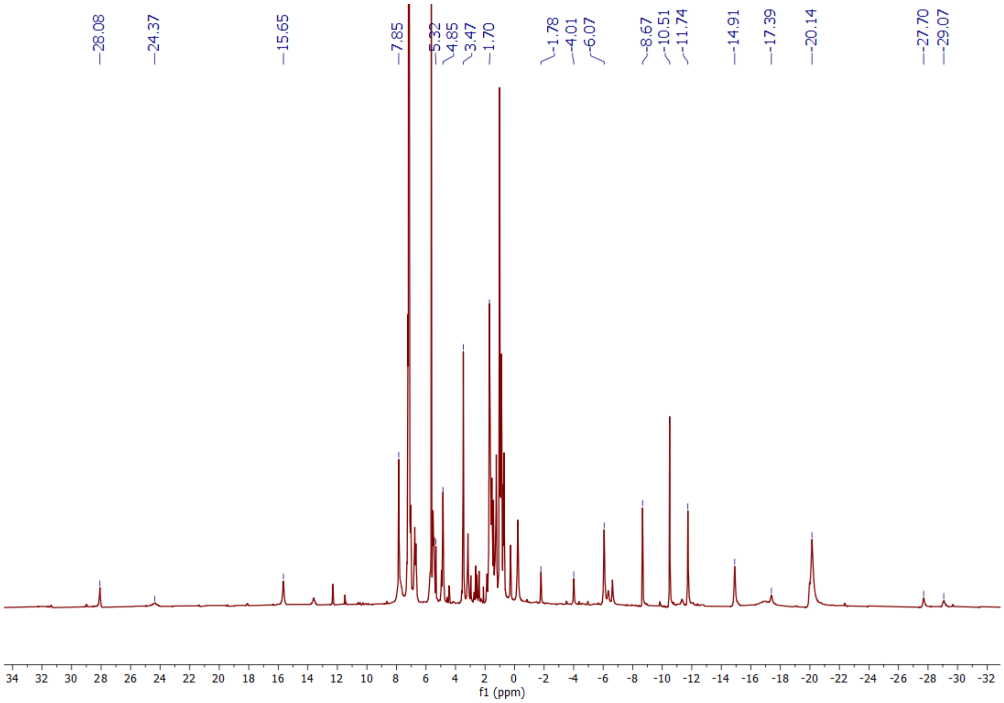

Figure S18. ^{1}H NMR (600.13 MHz, 298 K, C_6D_6) spectrum of reaction of [(^{DIPeP}BDI)Yb]$_2$(η^8,η^8-COT) (**4**) with COT at 60 °C after 16h.

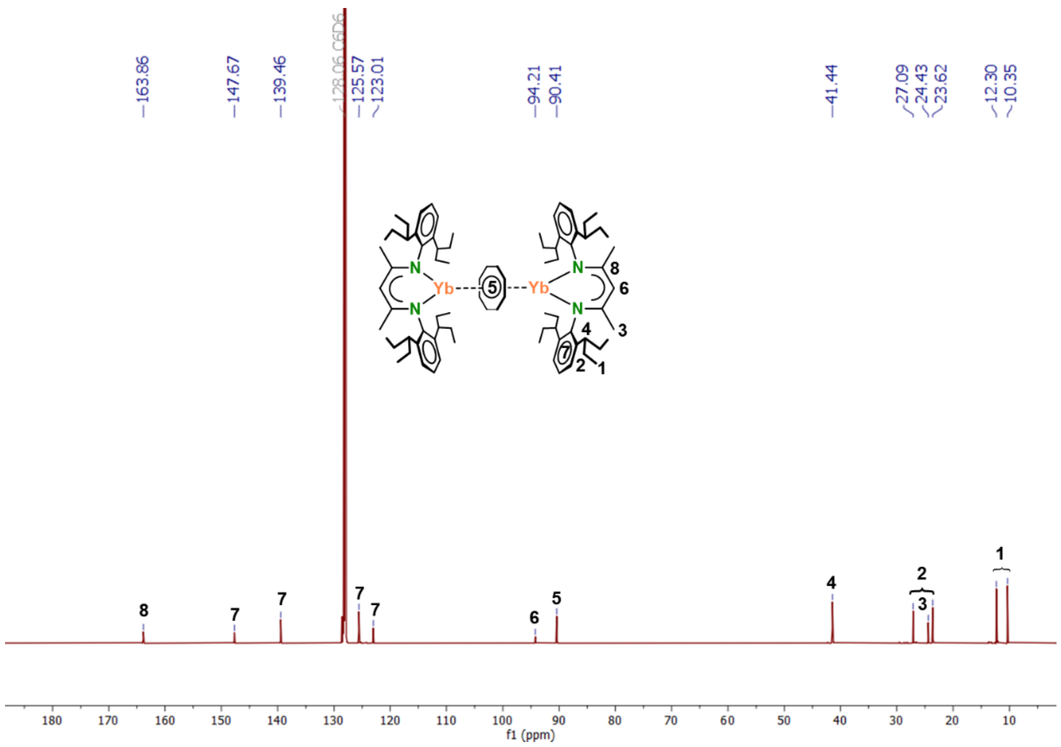

Figure S19. $^{13}C\{^{1}H\}$ NMR (150.92 MHz, 298 K, C_6D_6) of [(^{DIPeP}BDI)Yb]$_2$(η^8,η^8-COT) (**4**).

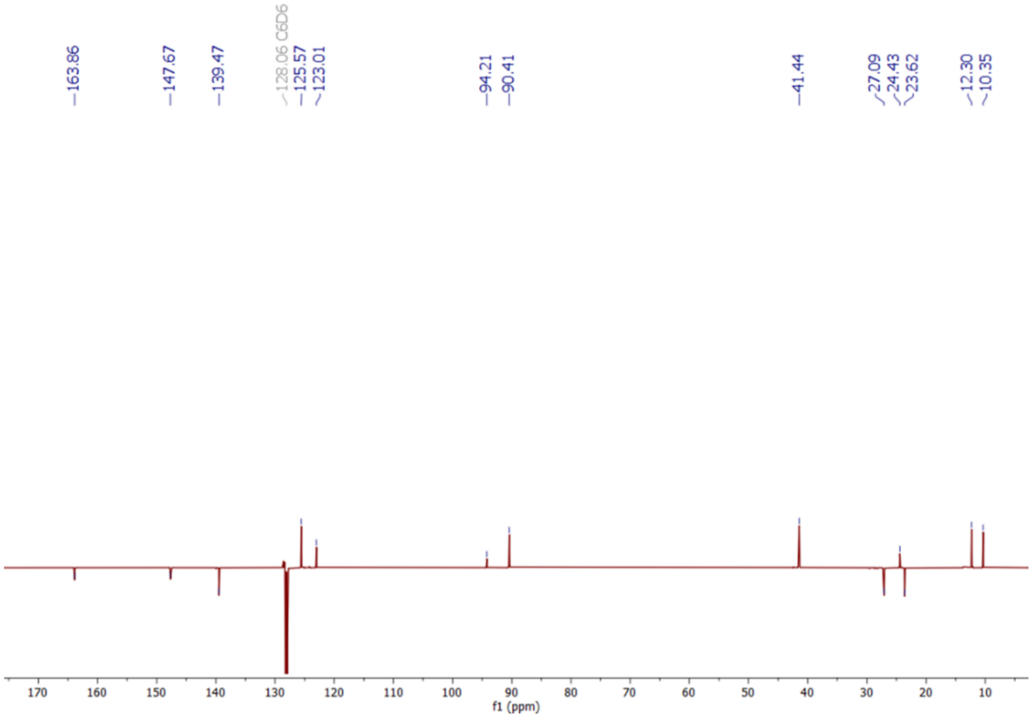

Figure S20. ^{13}C-APT NMR (150.92 MHz, 298 K, C_6D_6) of [(^{DIPeP}BDI)Yb]$_2$(η^8,η^8-COT) (**4**).

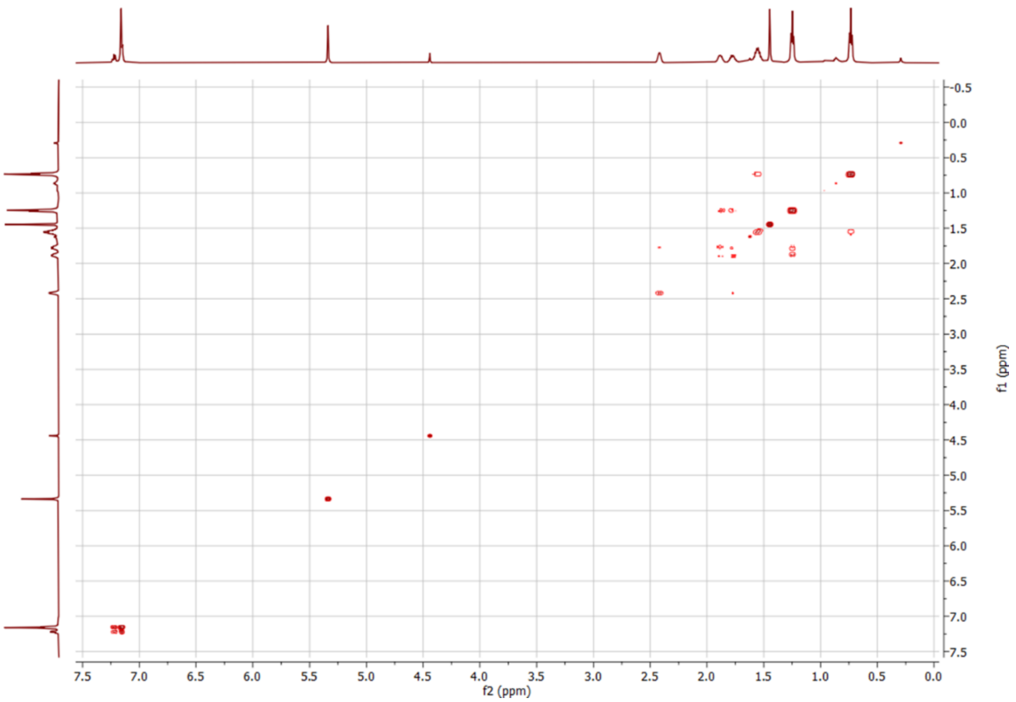

Figure S21. ^{1}H-^{1}H COSY NMR (600.13 MHz, 298 K, C_6D_6) of [(^{DIPeP}BDI)Yb]$_2$(η^8,η^8-COT) (**4**).

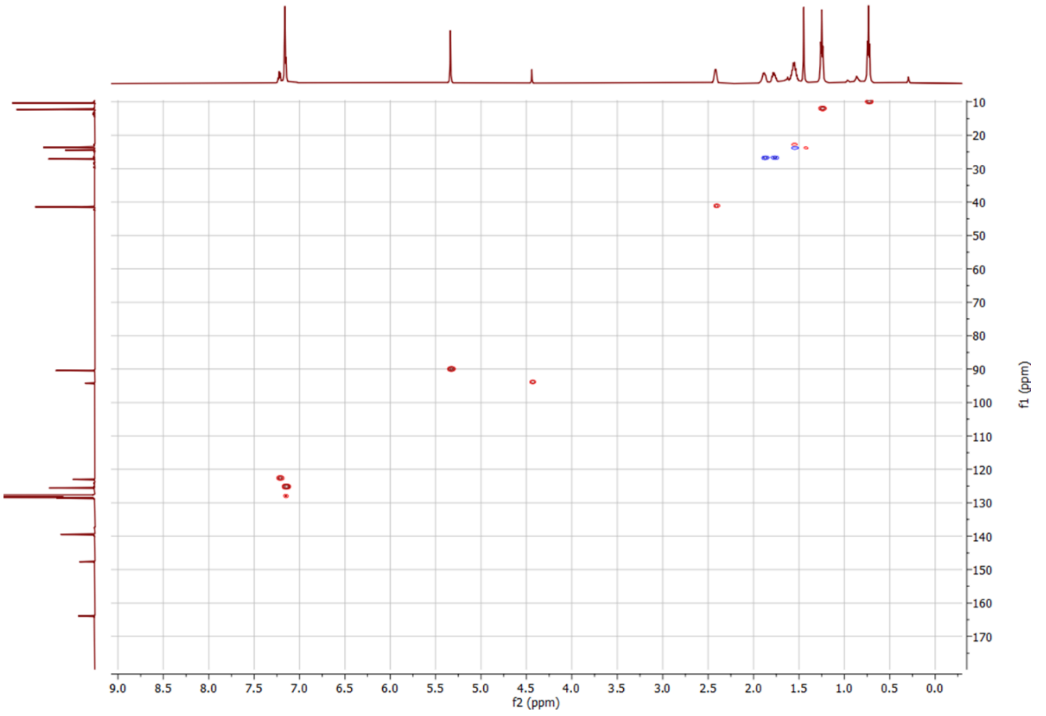

Figure S22. ^{1}H-^{13}C HSQC NMR (600.13/150.92 MHz, 298 K, C_6D_6) of [(^{DIPeP}BDI)Yb]$_2$(η^8,η^8-COT) (**4**).

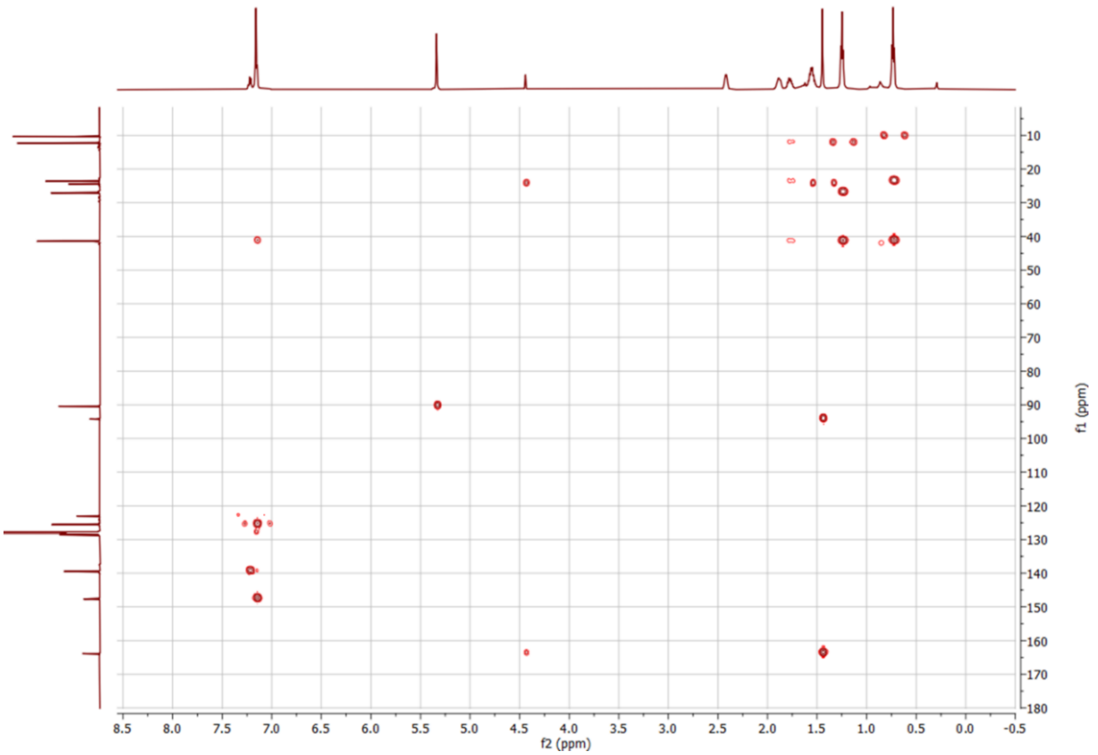

Figure S23. ^{1}H-^{13}C HMBC NMR (600.13/150.92 MHz, 298 K, C_6D_6) of [(^{DIPeP}BDI)Yb]$_2$(η^8,η^8-COT) (**4**).

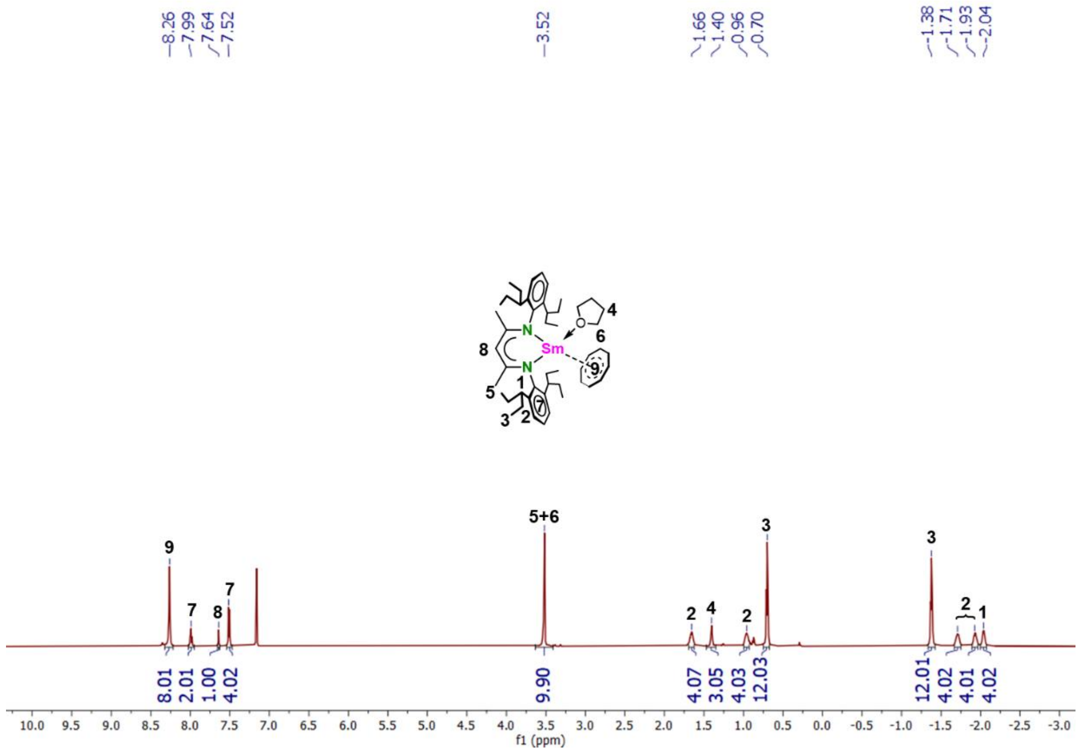

Figure S24. ^{1}H NMR (600.13 MHz, 298 K, C_6D_6) of (^{DIPeP}BDI)Sm(η^8-COT)(THF) (**5**).

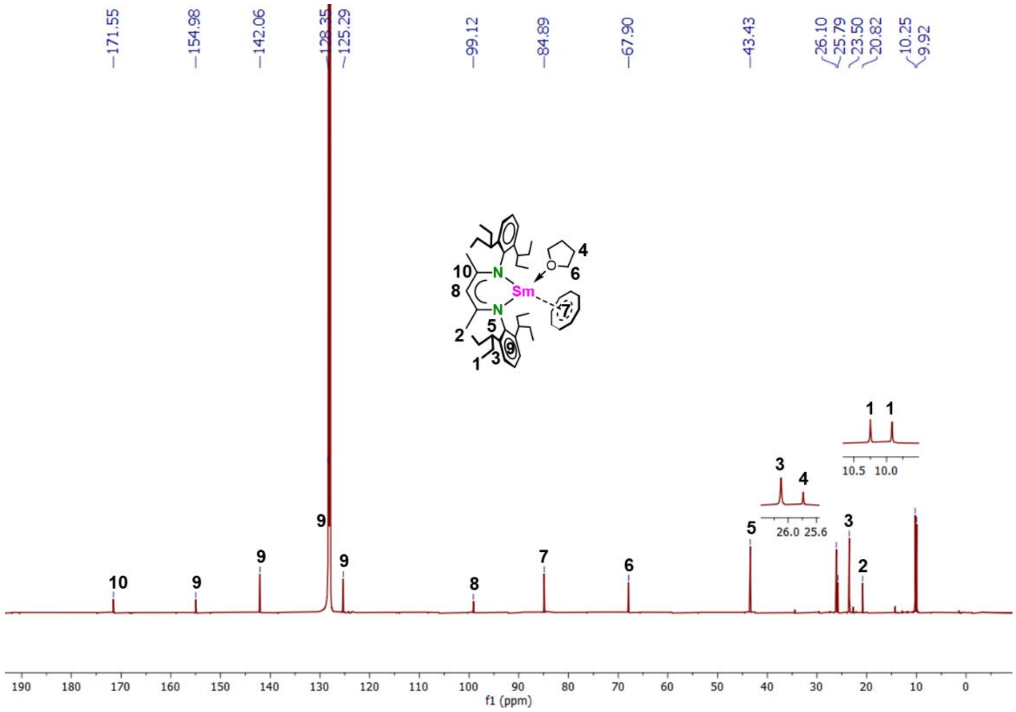

Figure S25. $^{13}C\{^1H\}$ NMR (150.92 MHz, 298 K, C_6D_6) of (^{DIPeP}BDI)Sm(η^8-COT)(THF) (**5**).

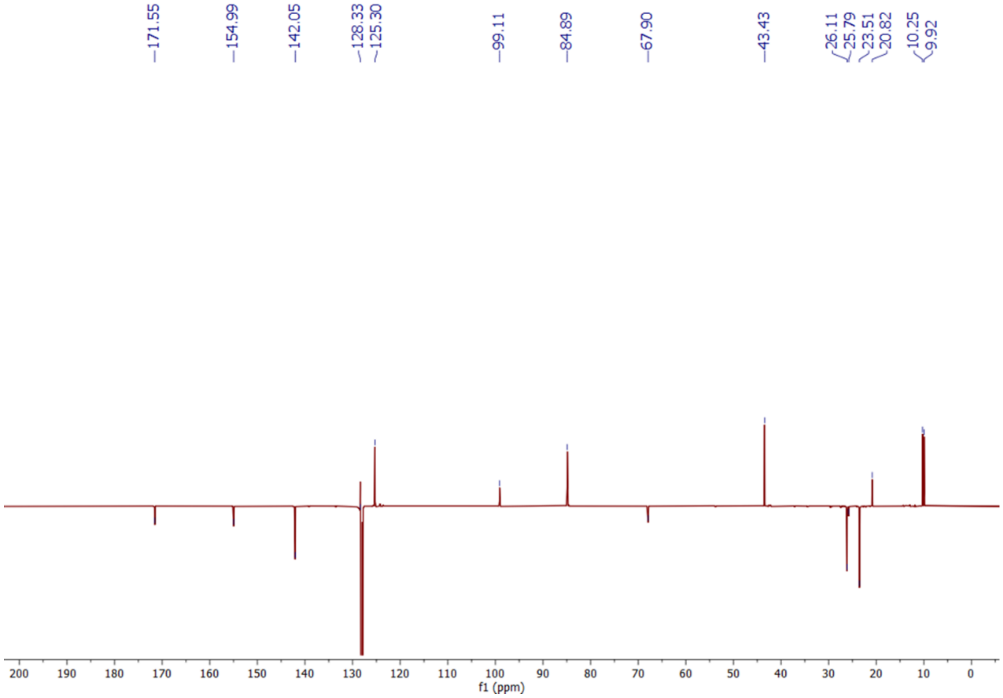

Figure S26. ^{13}C-APT NMR (150.92 MHz, 298 K, C_6D_6) of (^{DIPeP}BDI)Sm(η^8-COT)(THF) (**5**).

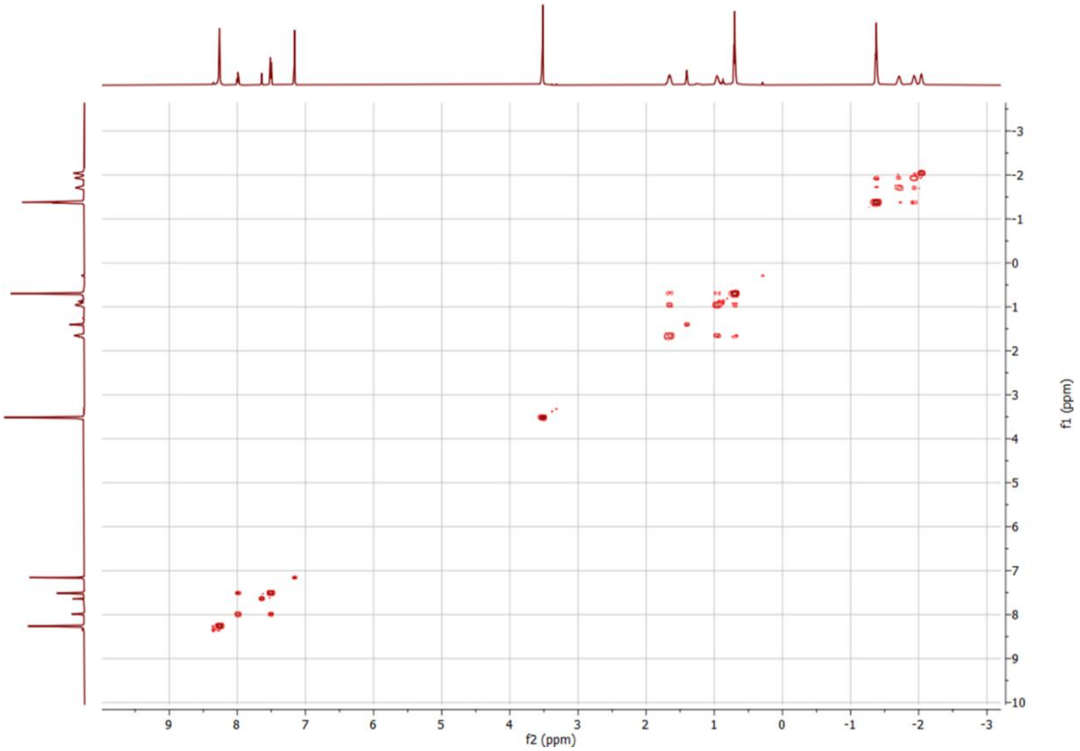

Figure S27. ^{1}H-^{1}H COSY NMR (600.13 MHz, 298 K, C_6D_6) of (^{DIPeP}BDI)Sm(η^8-COT)(THF) (**5**).

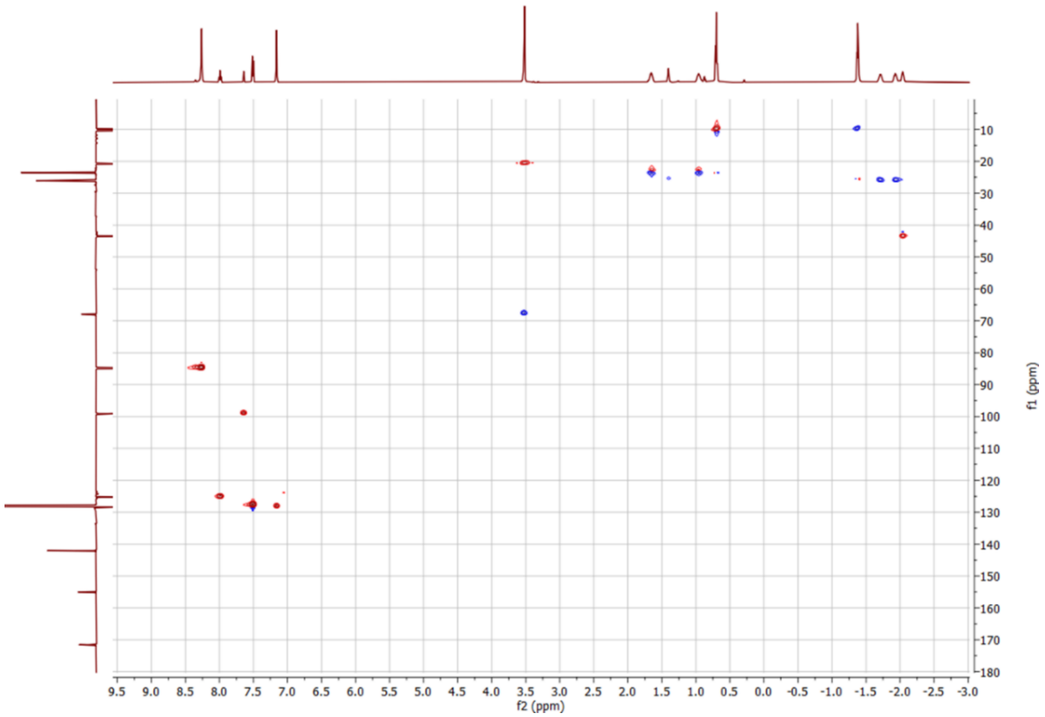

Figure S28. ^{1}H-^{13}C HSQC NMR (600.13/150.92 MHz, 298 K, C_6D_6) of (^{DIPeP}BDI)Sm(η^8-COT)(THF) (**5**).

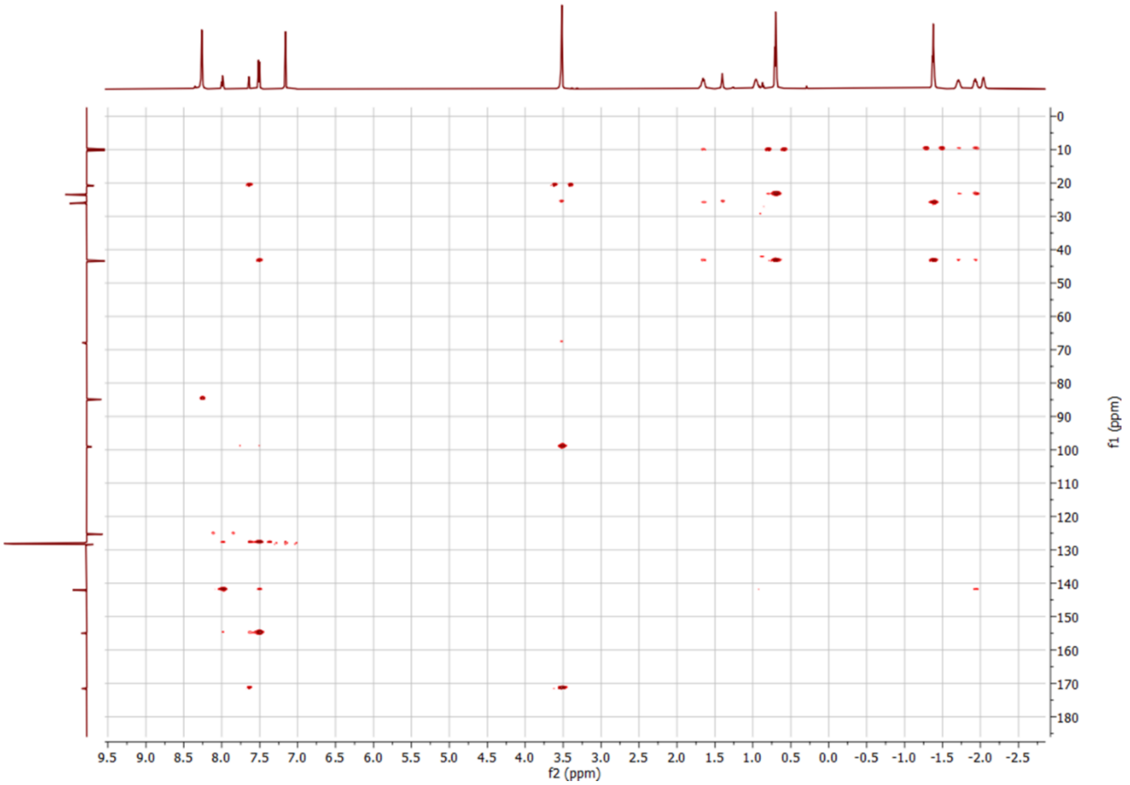

Figure S29. ^{1}H-^{13}C HMBC NMR (600.13/150.92 MHz, 298 K, C_6D_6) of (^{DIPeP}BDI)Sm(η^8-COT)(THF) (**5**).

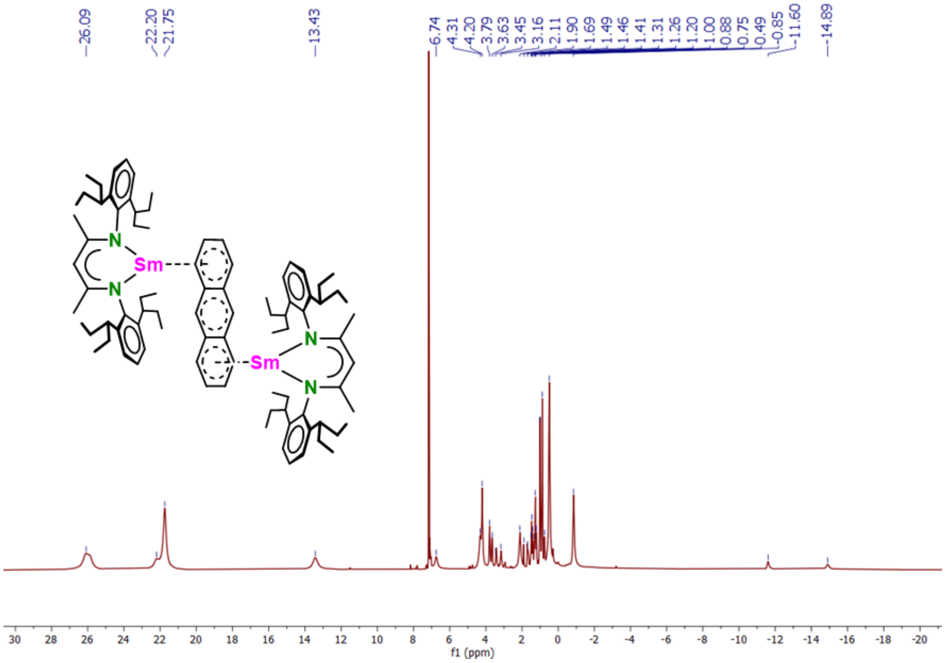

Figure S30. ^{1}H NMR (600.13 MHz, 298 K, C_6D_6) of [(^{DIPeP}BDI)Sm]$_2$(η^6,η^6-anthracene) (**6**).

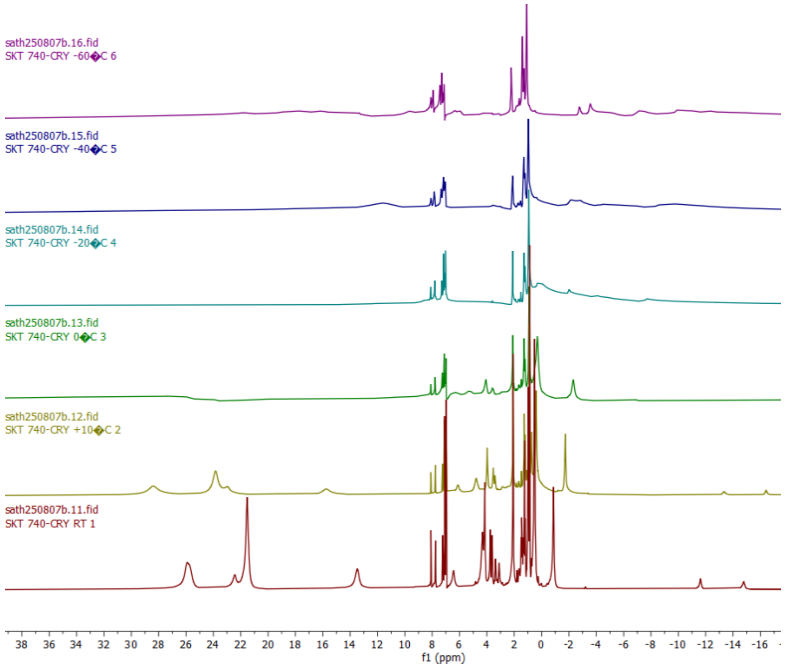

Figure S31. Variable temperature ^{1}H NMR (600.13 MHz, toluene-d_8) of [(^{DIPeP}BDI)Sm]$_2$(η^6,η^6-anthracene) (**6**) from RT to -60 ^{o}C.

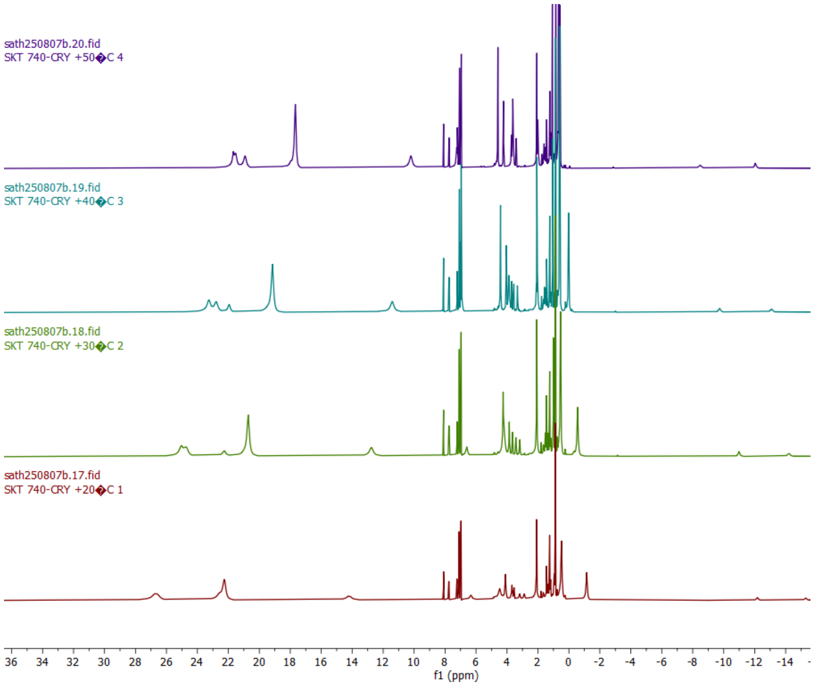

Figure S32. Variable temperature ^{1}H NMR (600.13 MHz, toluene-d_8) of [(^{DIPeP}BDI)Sm]$_2$(η^6,η^6-anthracene) (**6**) from RT to +50 ^{o}C.

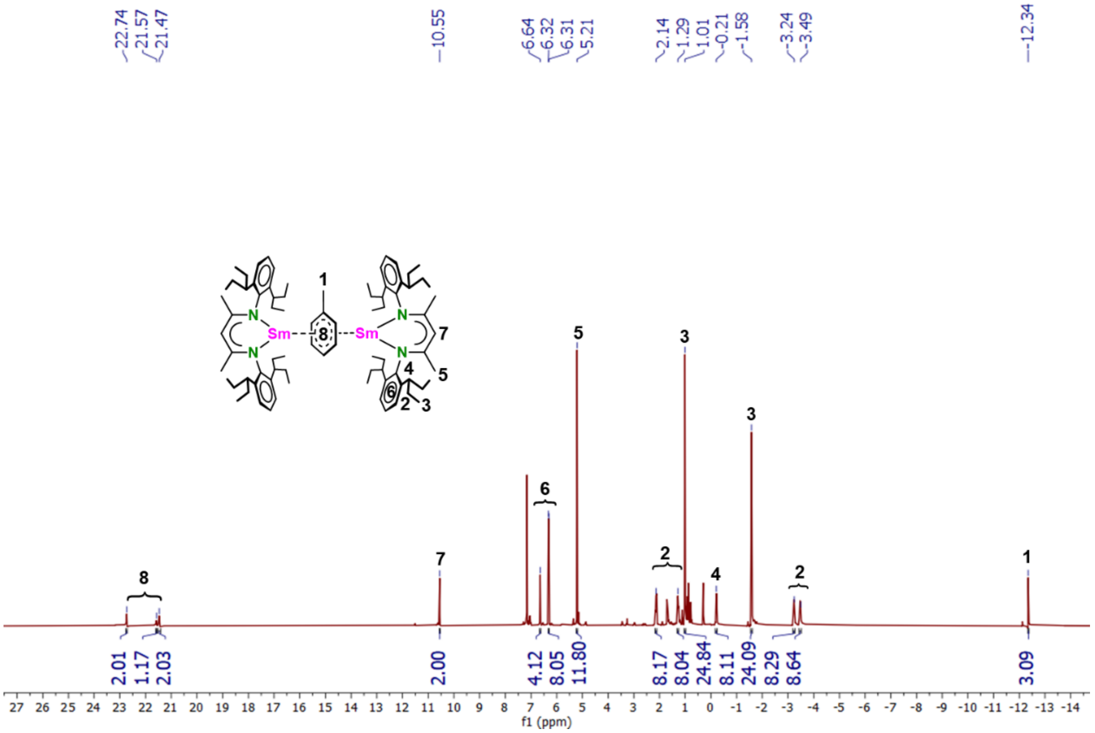

Figure S33. ^{1}H NMR (600.13 MHz, 298 K, C_6D_6) of solvent free [(^{DIPeP}BDI)Sm]$_2$(η^6,η^6-toluene) (**7**).

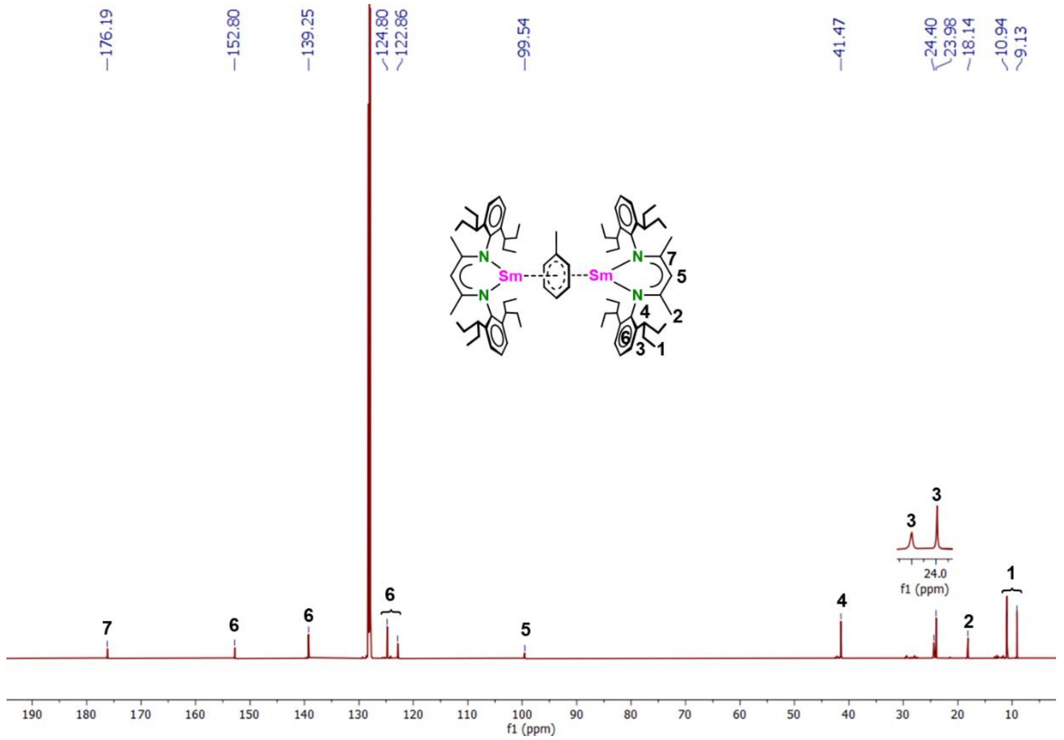

Figure S34. ^{13}C{^{1}H} NMR (150.92 MHz, 298 K, C_6D_6) of solvent free [(^{DIPeP}BDI)Sm]$_2$(η^6,η^6-toluene) (**7**).

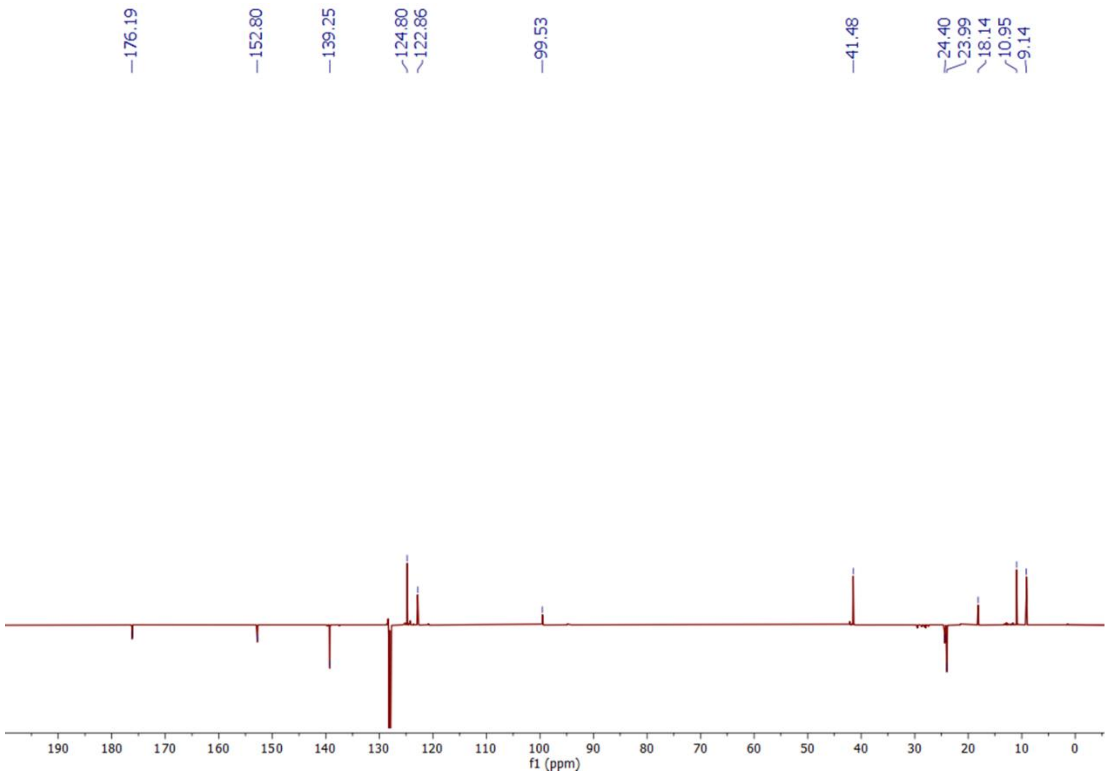

Figure S35. ^{13}C-APT NMR (150.92 MHz, 298 K, C_6D_6) of solvent free [(^{DIPeP}BDI)Sm]$_2$(η^6,η^6-toluene)(**7**).

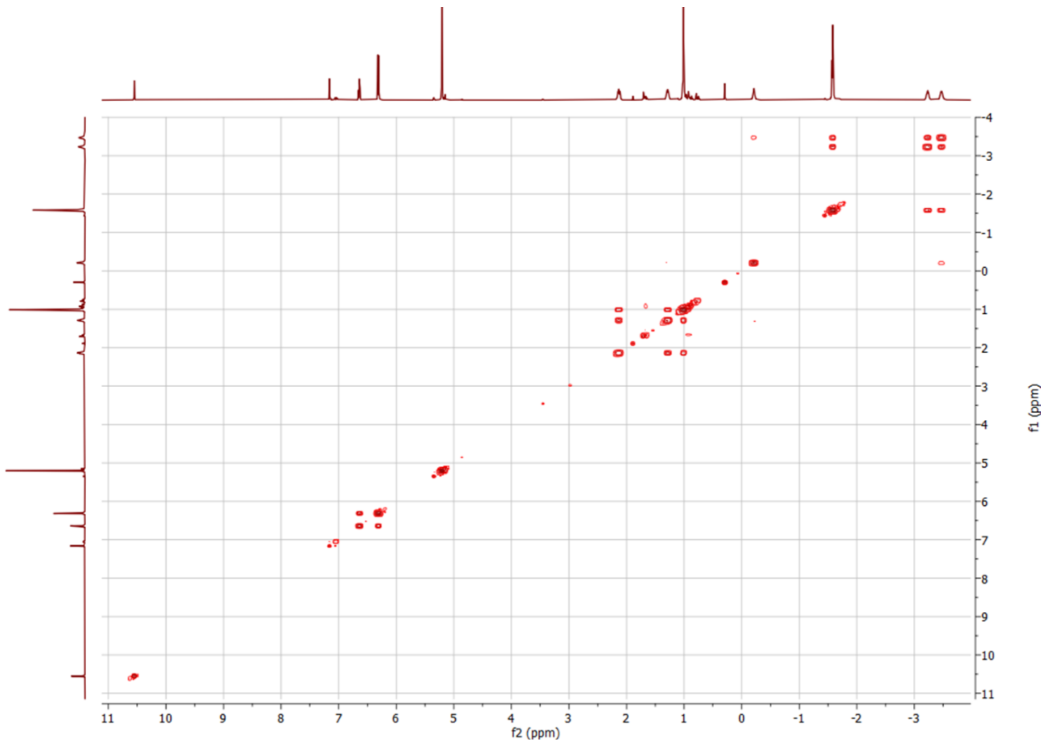

Figure S36. ^{1}H-^{1}H COSY NMR (600.13 MHz, 298 K, C_6D_6) of solvent free [(^{DIPeP}BDI)Sm]$_2$(η^6,η^6-toluene) (**7**).

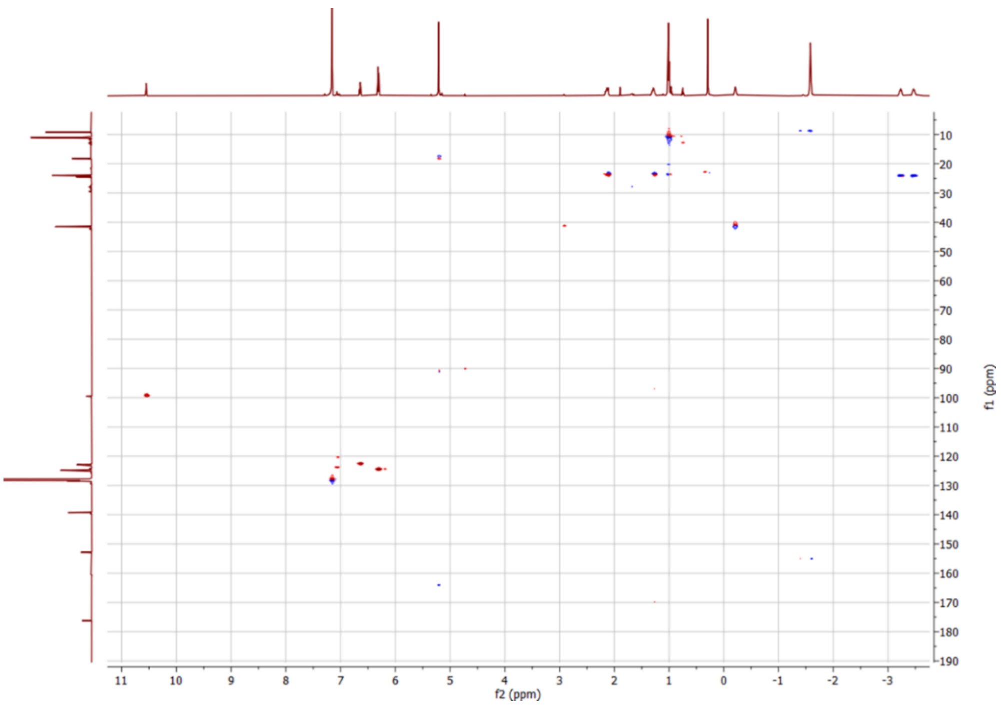

Figure S37. ^{1}H-^{13}C HSQC NMR (600.13/150.92 MHz, 298 K, C_6D_6) of solvent free ([(^{DIPeP}BDI)Sm]$_2$(η^6,η^6-toluene) (**7**).

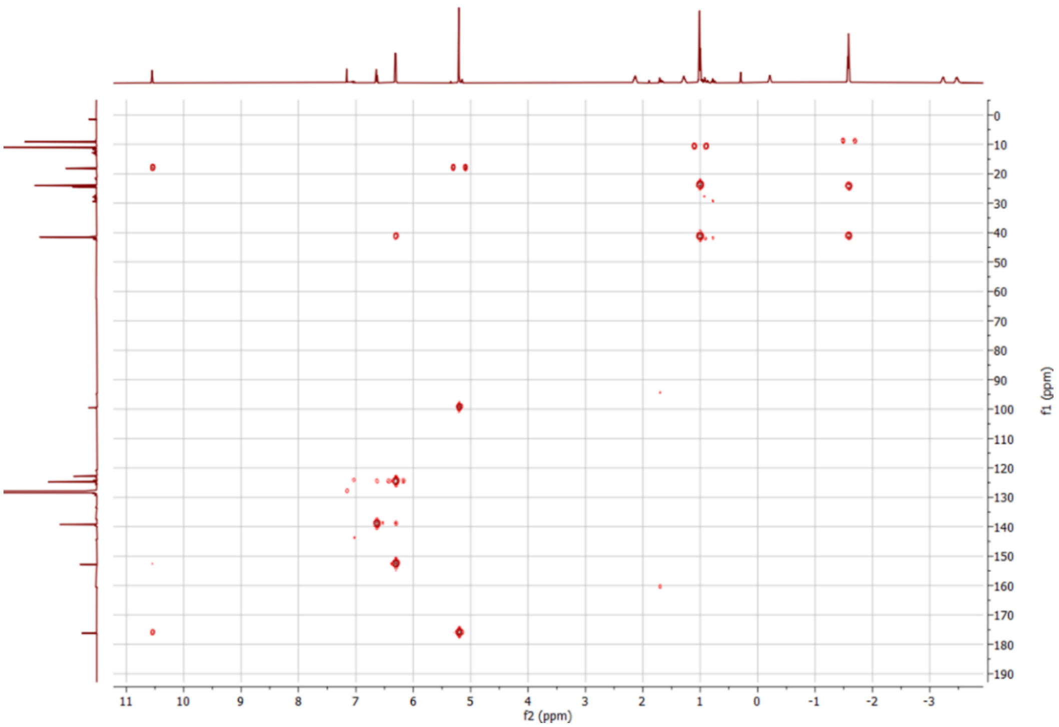

Figure S38. ^{1}H-^{13}C HMBC NMR (600.13/150.92 MHz, 298 K, C_6D_6) of solvent free [(^{DIPeP}BDI)Sm]$_2$(η^6,η^6-toluene) (**7**).

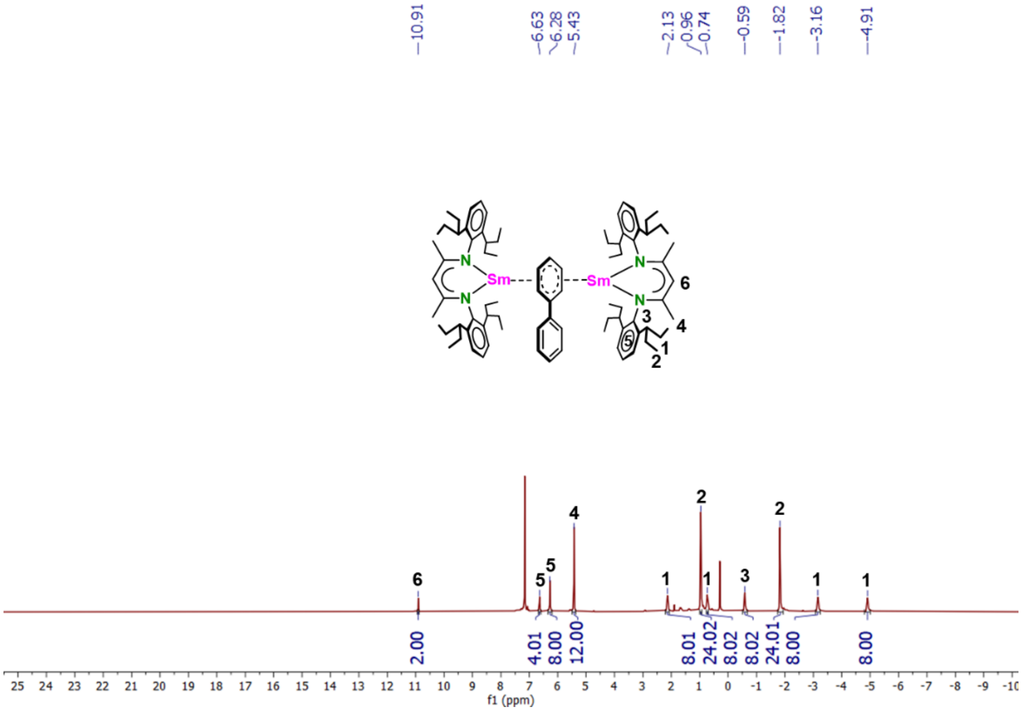

Figure S39. ^{1}H NMR (600.13 MHz, 298 K, C_6D_6) of solvent free $[(^{DIPeP}BDI)Sm]_2(\eta^6,\eta^6$-biphenyl) (**8**).

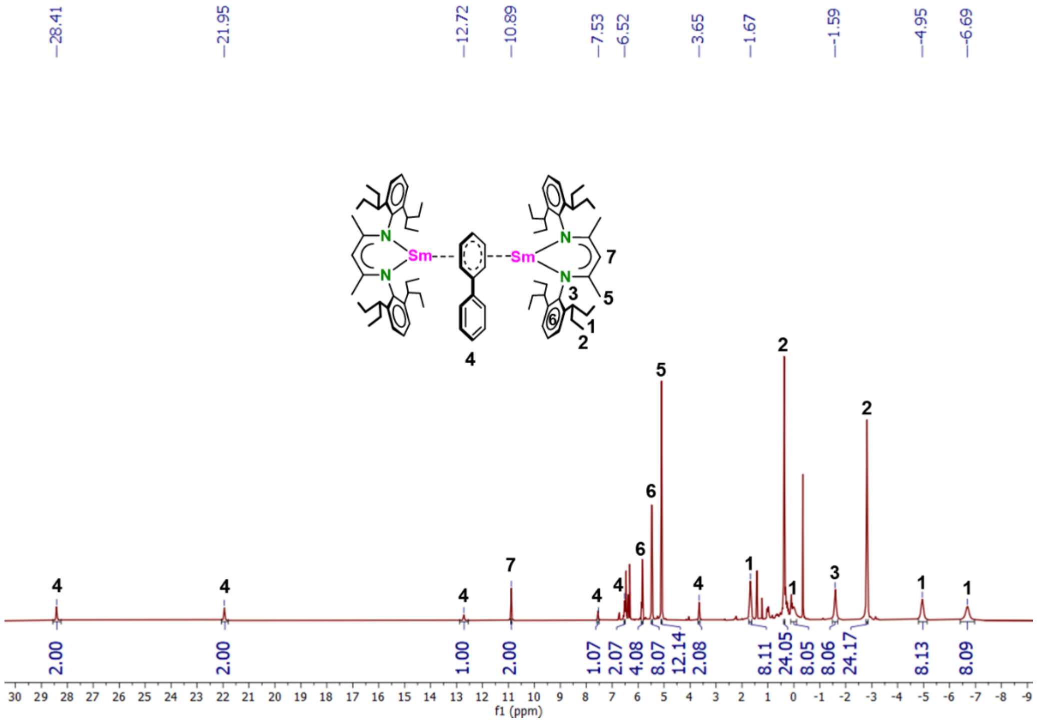

Figure S40. ^{1}H NMR (600.13 MHz, 243 K, C_7D_8) of solvent free $[(^{DIPeP}BDI)Sm]_2(\eta^6,\eta^6$-biphenyl)(**8**).

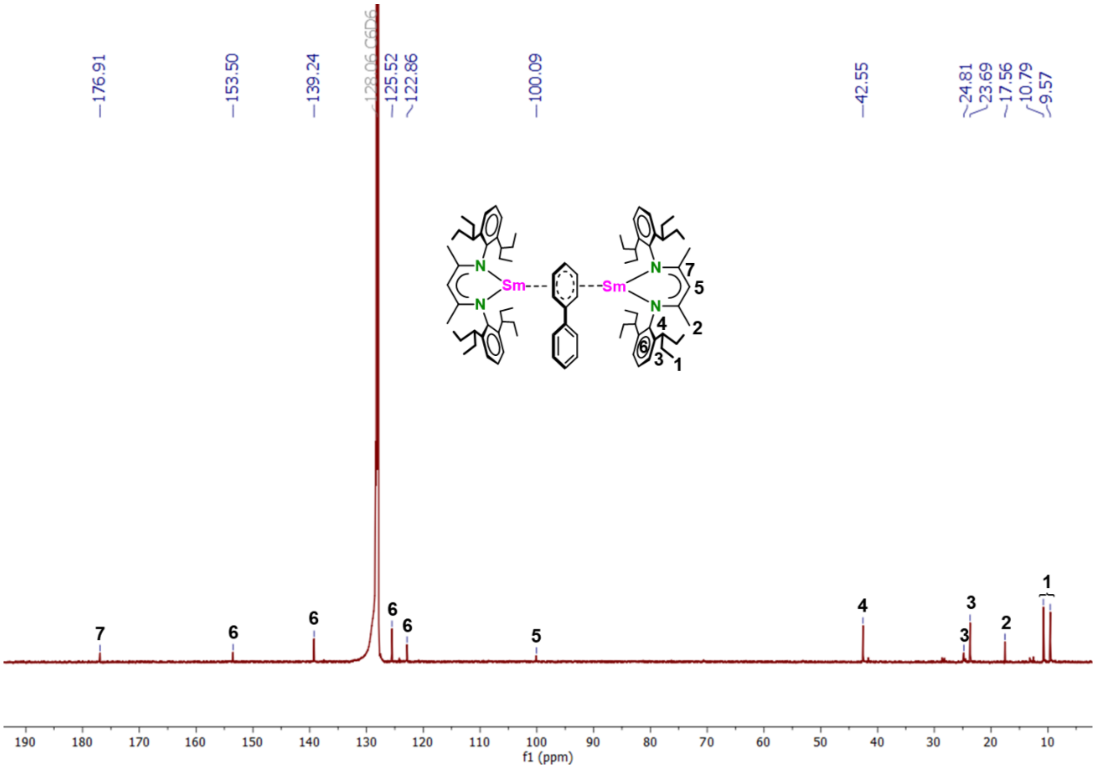

Figure S41. $^{13}C\{^{1}H\}$ NMR (150.92 MHz, 298 K, C_6D_6) of solvent free $[(^{DIPeP}BDI)Sm]_2(\eta^6,\eta^6$-biphenyl) (**8**).

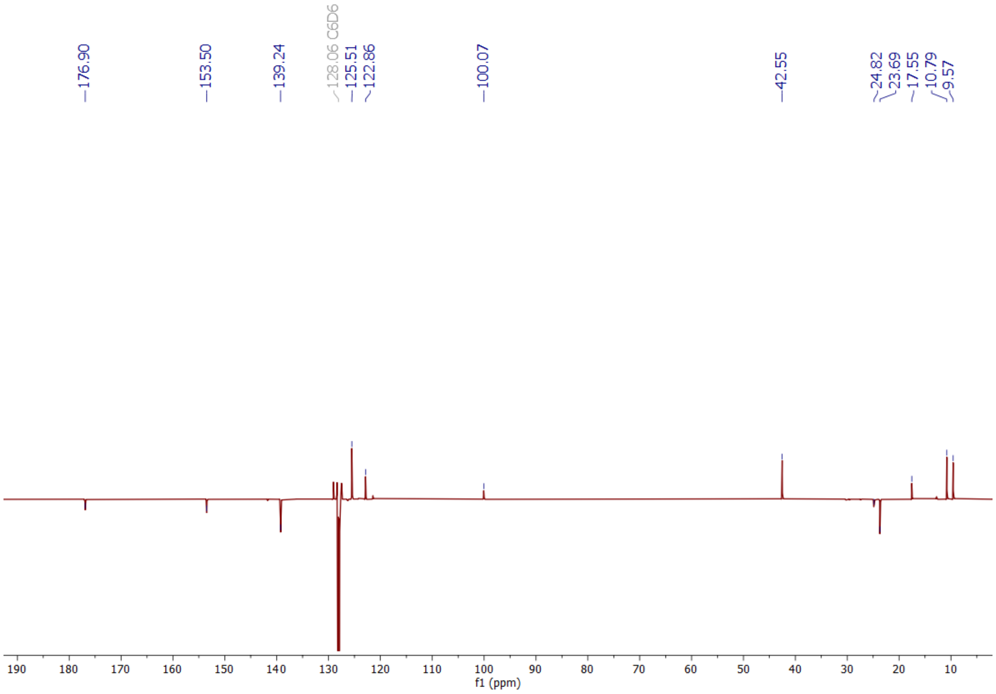

Figure S42. ^{13}C-APT NMR (150.92 MHz, 298 K, C_6D_6) of solvent free $[(^{DIPeP}BDI)Sm]_2(\eta^6,\eta^6$-biphenyl) (**8**).

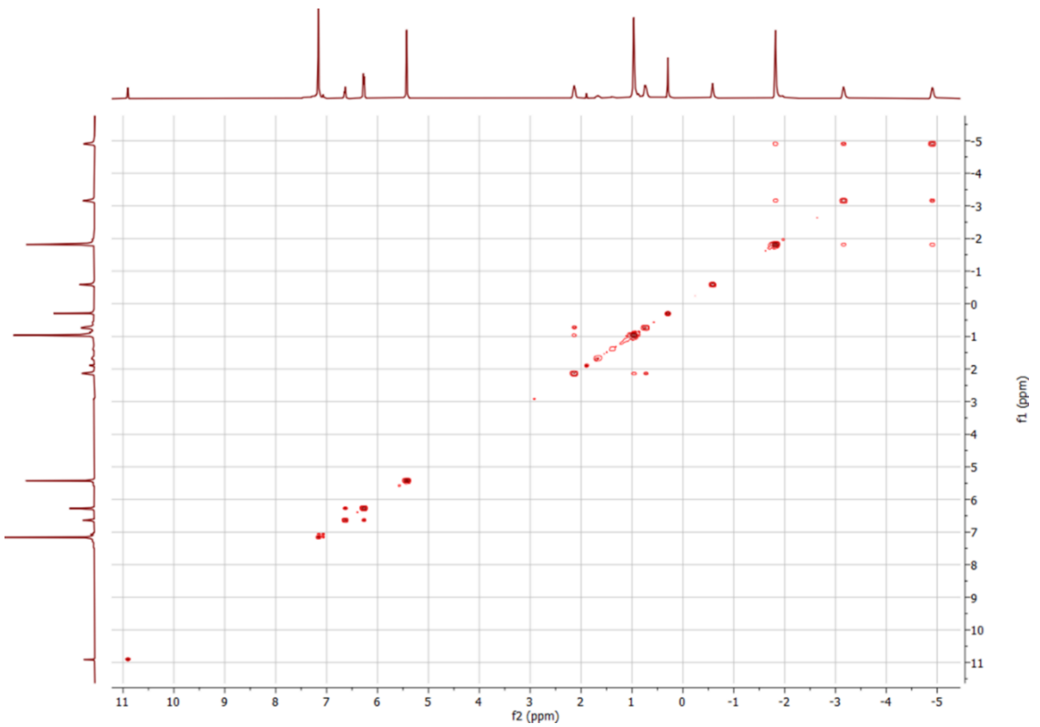

Figure S43. ^{1}H-^{1}H COSY NMR (600.13 MHz, 298 K, C_6D_6) of solvent free [(^{DIPeP}BDI)Sm]$_2$(η^6,η^6-biphenyl)(**8**).

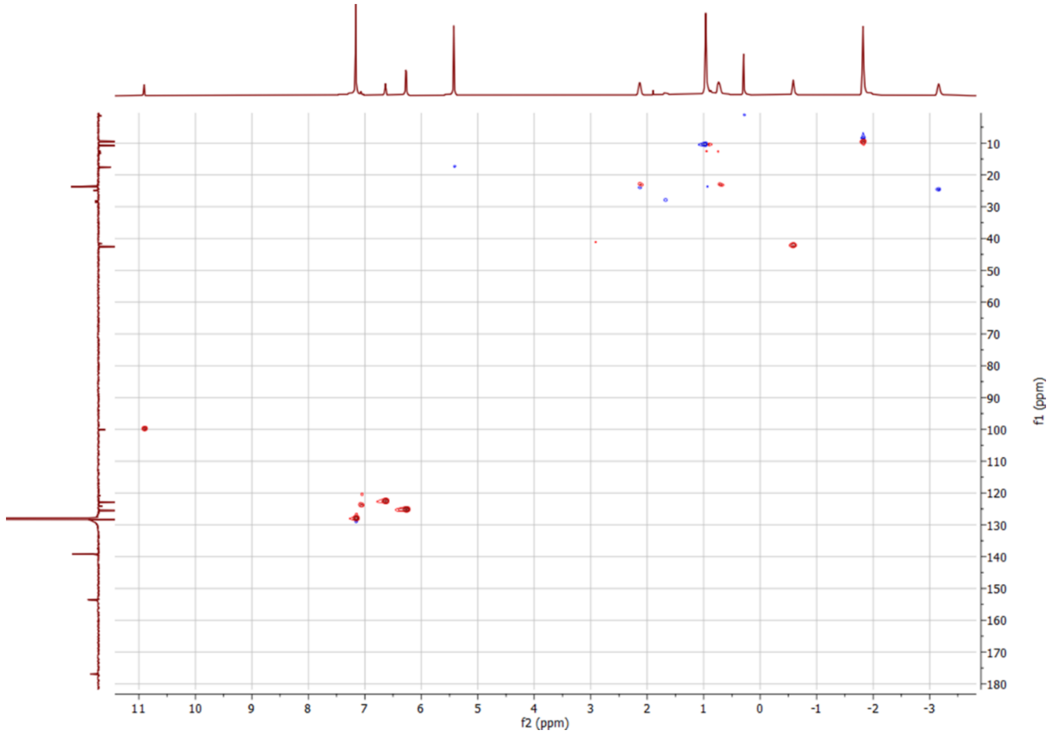

Figure S44. ^{1}H-^{13}C HSQC NMR (600.13/150.92 MHz, 298 K, C_6D_6) of solvent free [(^{DIPeP}BDI)Sm]$_2$(η^6,η^6-biphenyl)(**8**).

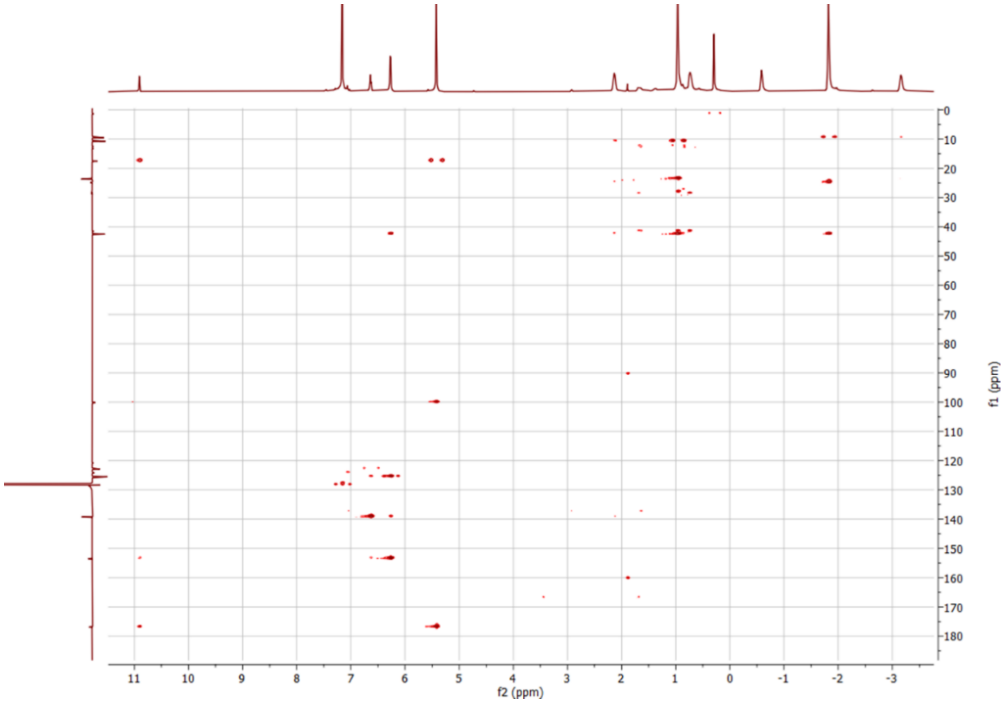

Figure S45. ^1H-^{13}C HMBC NMR (600.13/150.92 MHz, 298 K, C_6D_6) of solvent free [(^{DIPeP}BDI)Sm]$_2$(η^6,η^6-biphenyl)(**8**).

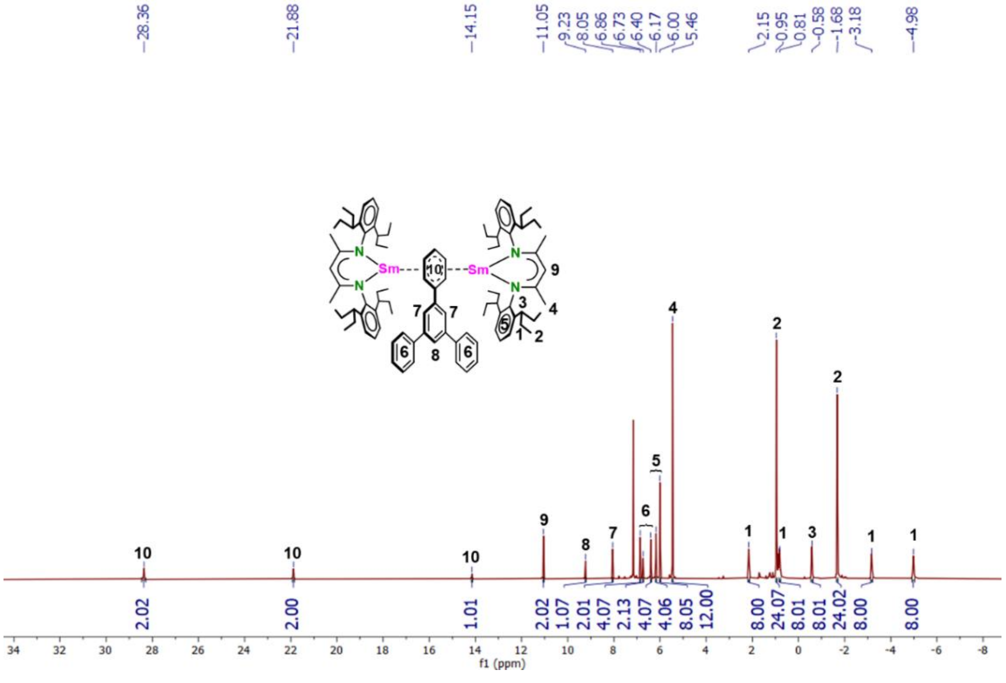

Figure S46. ^1H NMR (600.13 MHz, 298 K, C_6D_6) of [(^{DIPeP}BDI)Sm]$_2$(η^6,η^6-1,3,5-triphenylbenzene)(**9**).

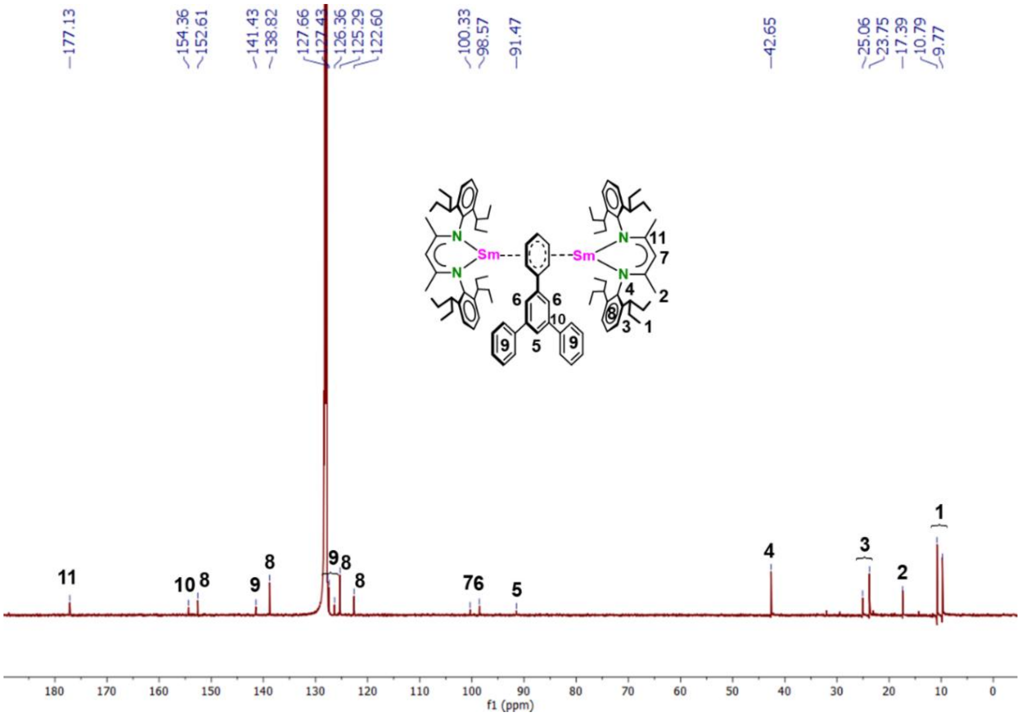

Figure S47. $^{13}C\{^{1}H\}$ NMR (150.92 MHz, 298 K, C_6D_6) of [(^{DIPeP}BDI)Sm]$_2$(η^6,η^6-1,3,5-triphenylbenzene) (**9**).

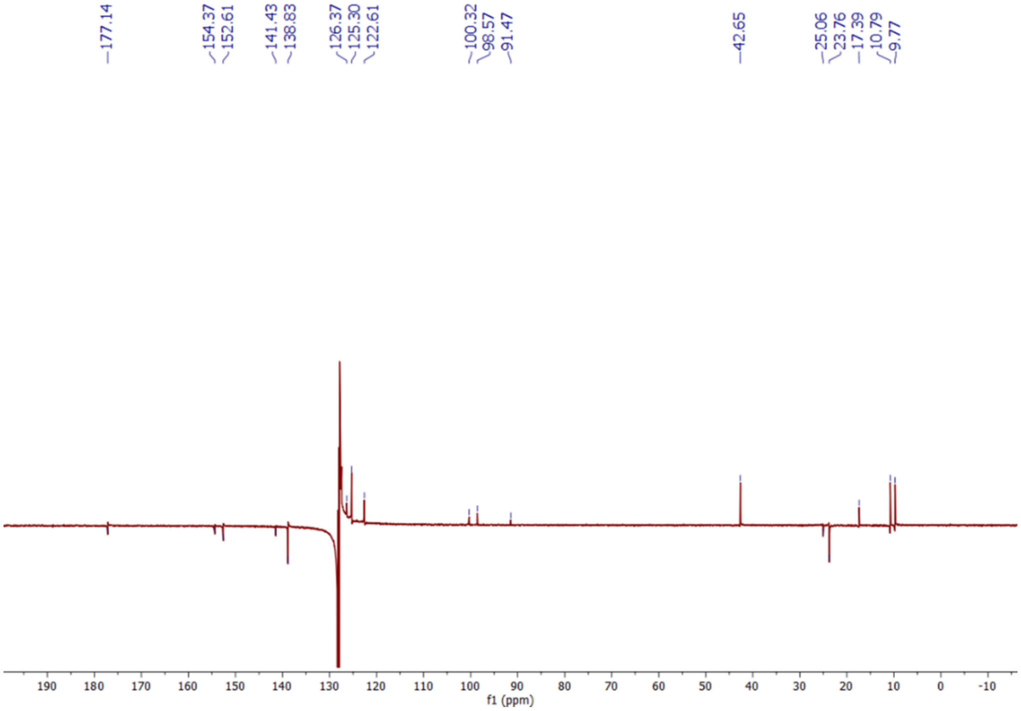

Figure S48. ^{13}C-APT NMR (150.92 MHz, 298 K, C_6D_6) of [(^{DIPeP}BDI)Sm]$_2$(η^6,η^6-1,3,5-triphenylbenzene) (**9**).

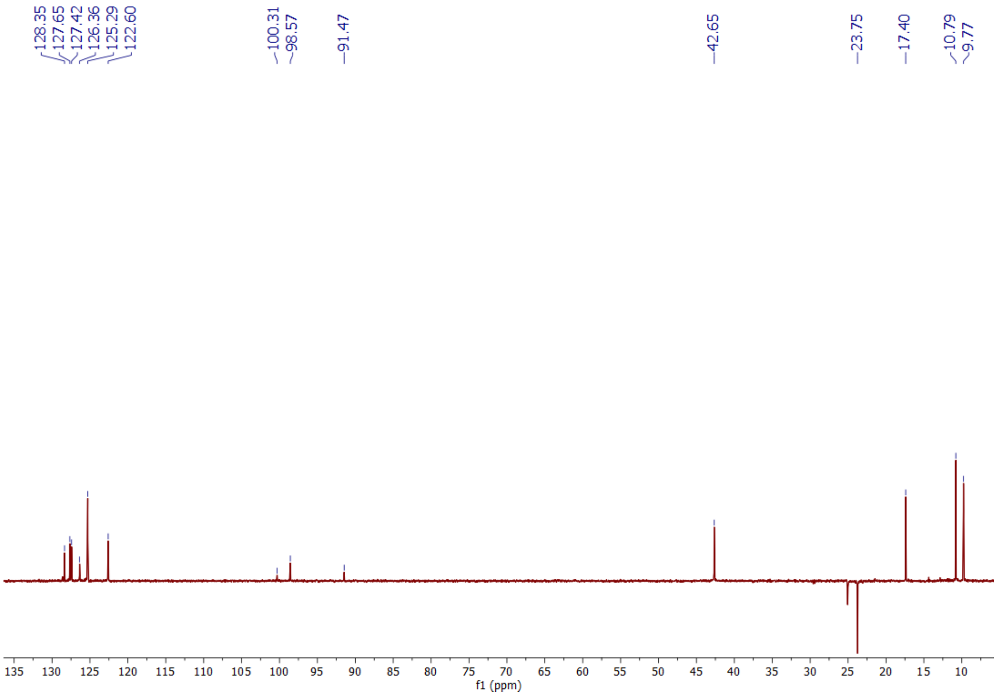

Figure S49. ^{13}C-DEPT NMR (150.92 MHz, 298 K, C_6D_6) spectrum of [(^{DIPeP}BDI)Sm]$_2$(η^6,η^6-1,3,5-triphenylbenzene) (**9**).

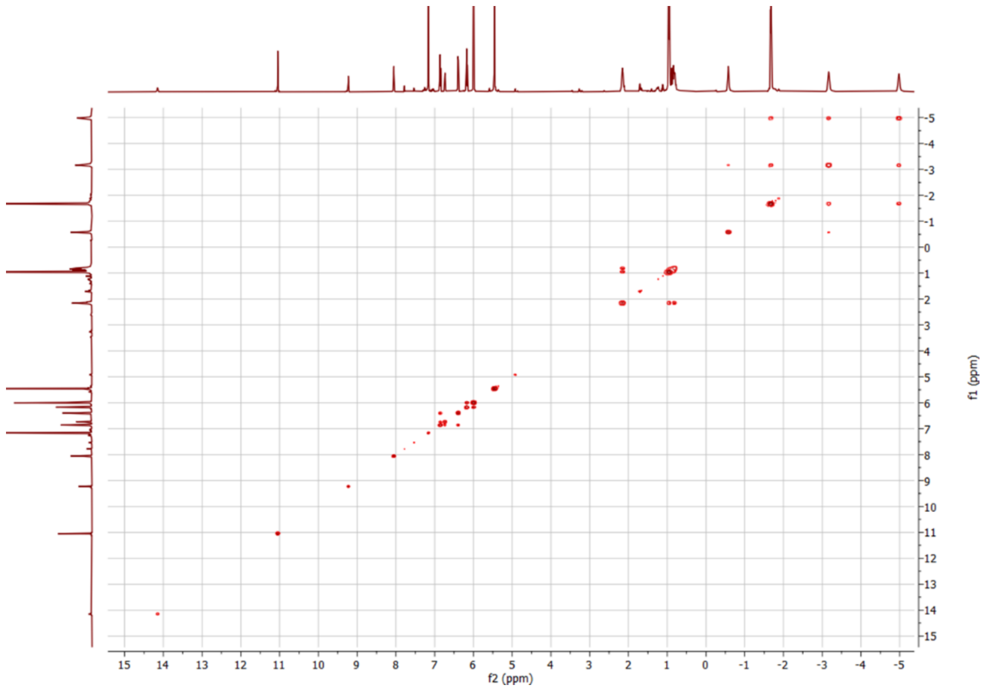

Figure S50. ^{1}H-^{1}H COSY NMR (600.13 MHz, 298 K, C_6D_6) of [(^{DIPeP}BDI)Sm]$_2$(η^6,η^6-1,3,5-triphenylbenzene) (**9**).

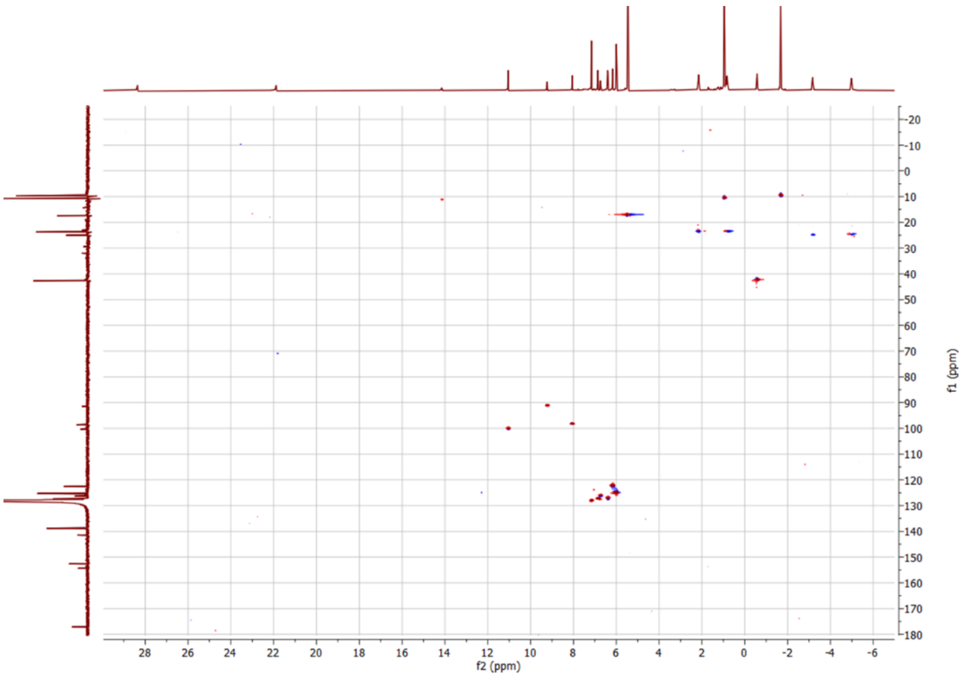

Figure S51. ^{1}H-^{13}C HSQC NMR (600.13/150.92 MHz, 298 K, C_6D_6) of [(^{DIPeP}BDI)Sm]$_2$(η^6,η^6-1,3,5-triphenylbenzene) (**9**).

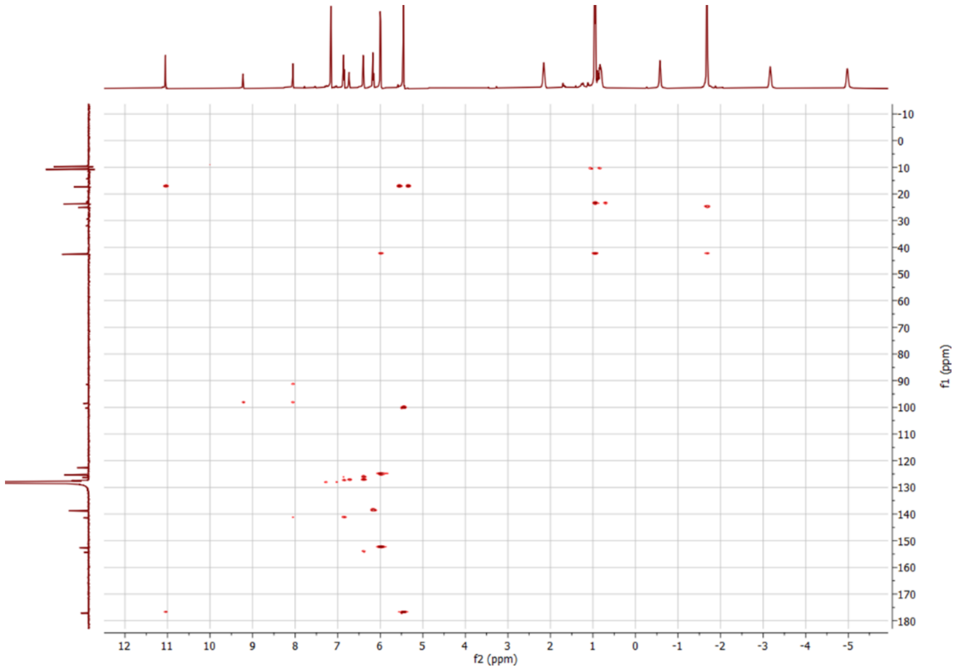

Figure S52. ^{1}H-^{13}C HMBC NMR (600.13/150.92 MHz, 298 K, C_6D_6) of [(^{DIPeP}BDI)Sm]$_2$(η^6,η^6-1,3,5-triphenylbenzene) (**9**).

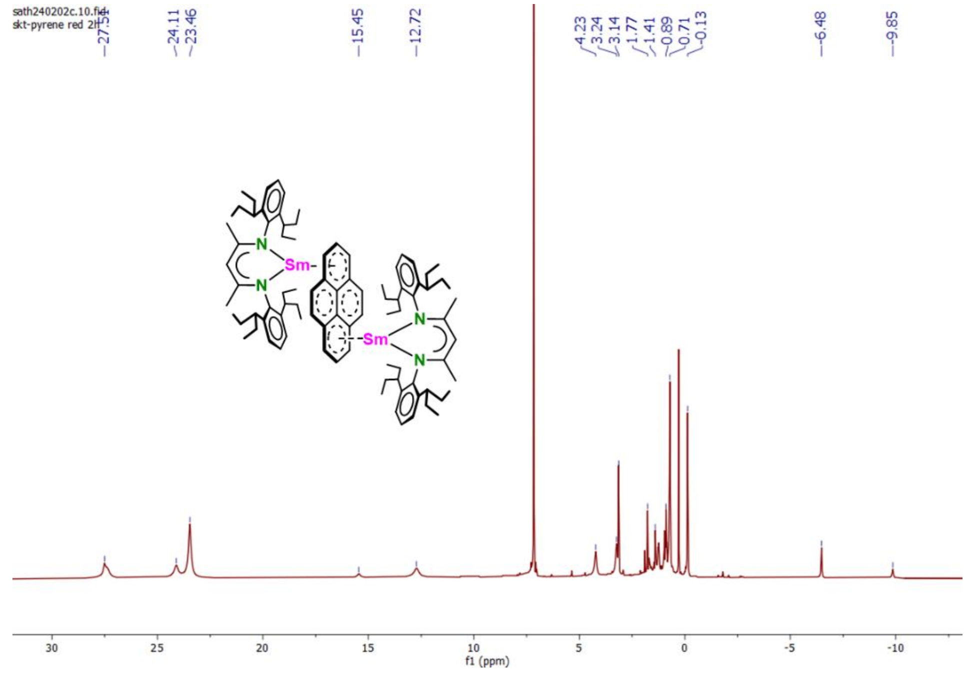

Figure S53. ^{1}H NMR (600.13 MHz, 298 K, C_6D_6) of solvent free [(^{DIPeP}BDI)Sm]$_2$(η^6,η^6-pyrene) (**10**).

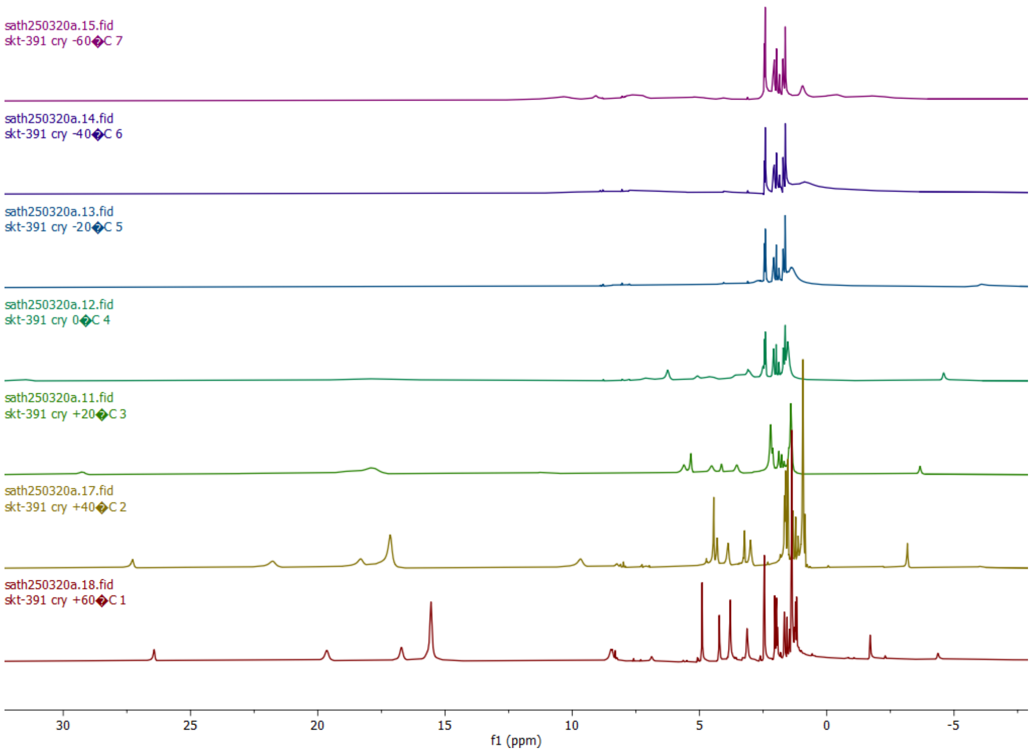

Figure S54. ^{1}H NMR (600.13 MHz, C_7D_8) of solvent free [(^{DIPeP}BDI)Sm]$_2$(η^6,η^6-pyrene)(**10**).

3b. IR data

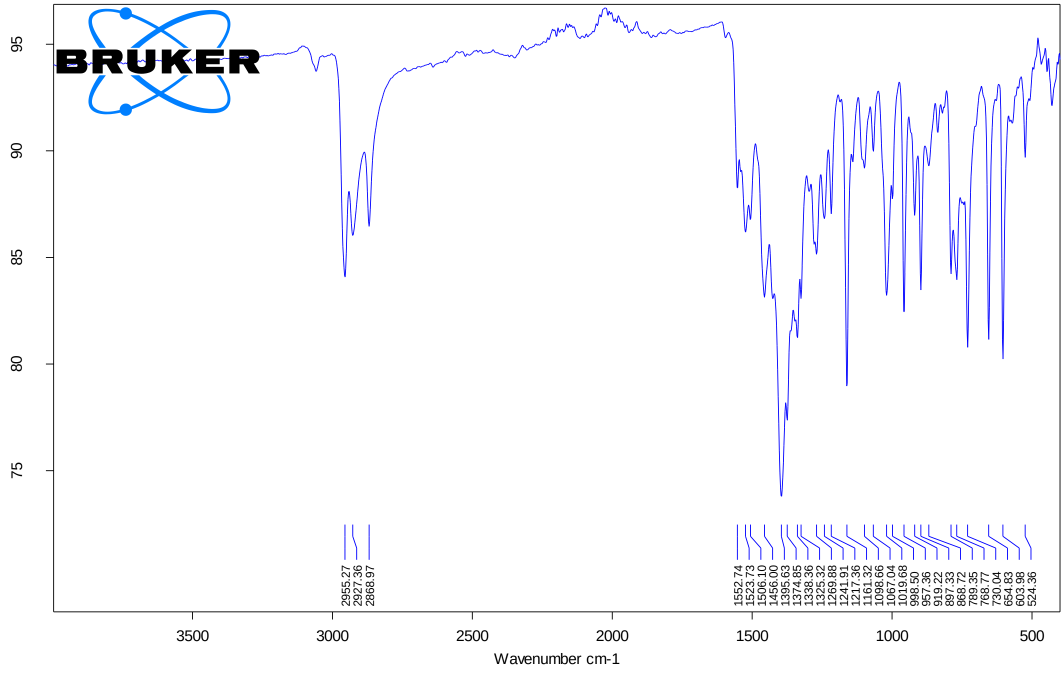

Figure S55. IR (ATR) spectrum of **1**

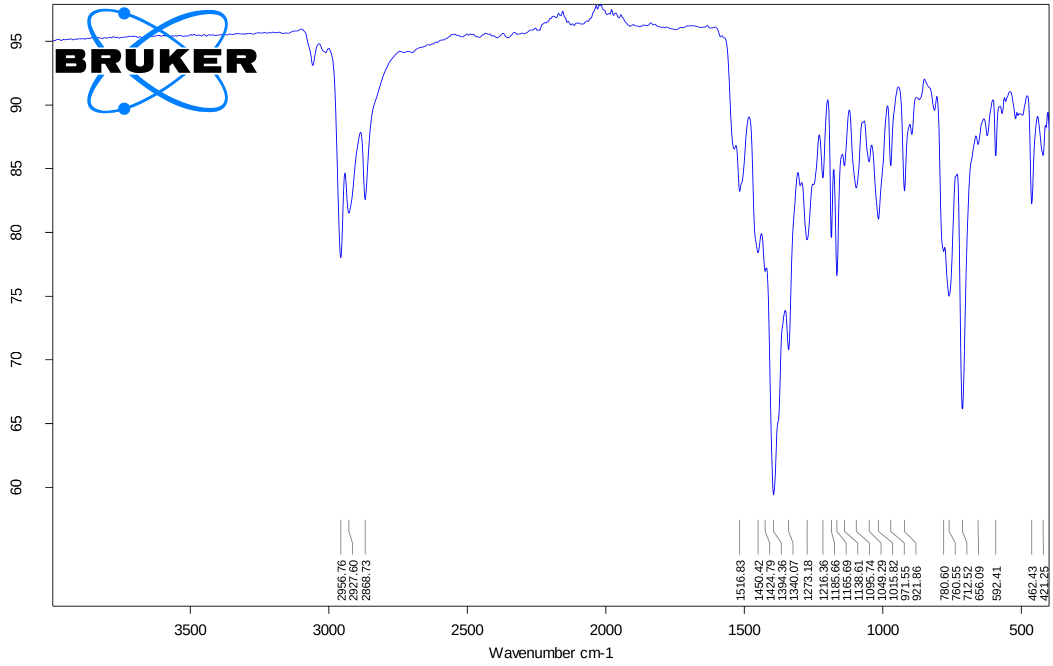

Figure S56. IR (ATR) spectrum of **2**

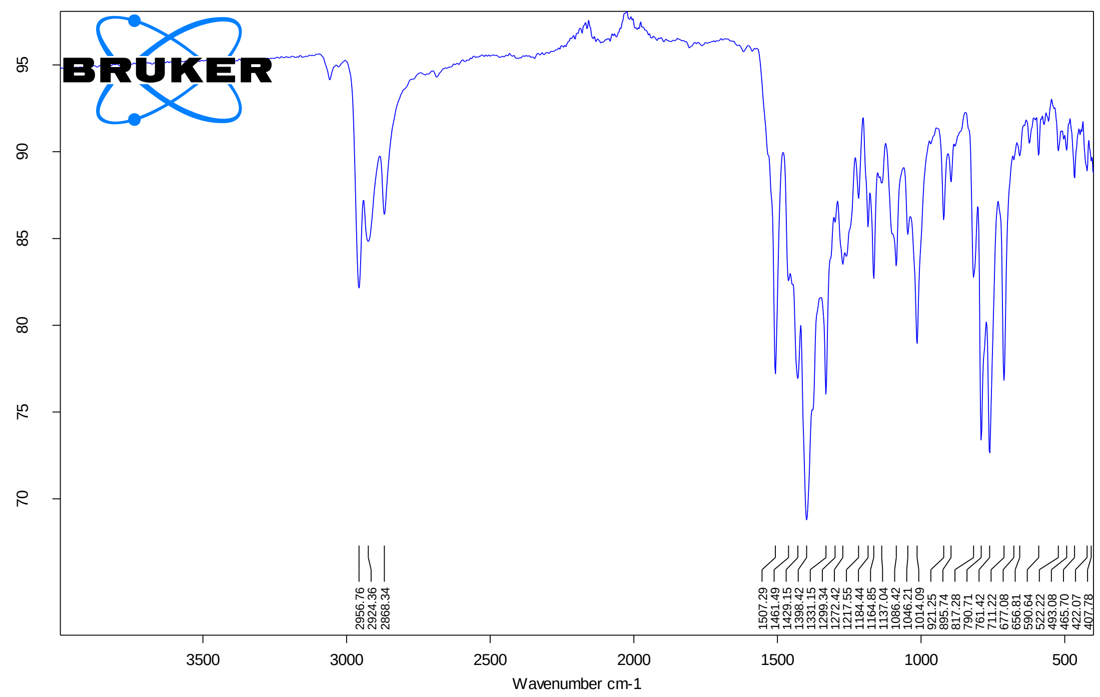

Figure S57. IR (ATR) spectrum of **3**

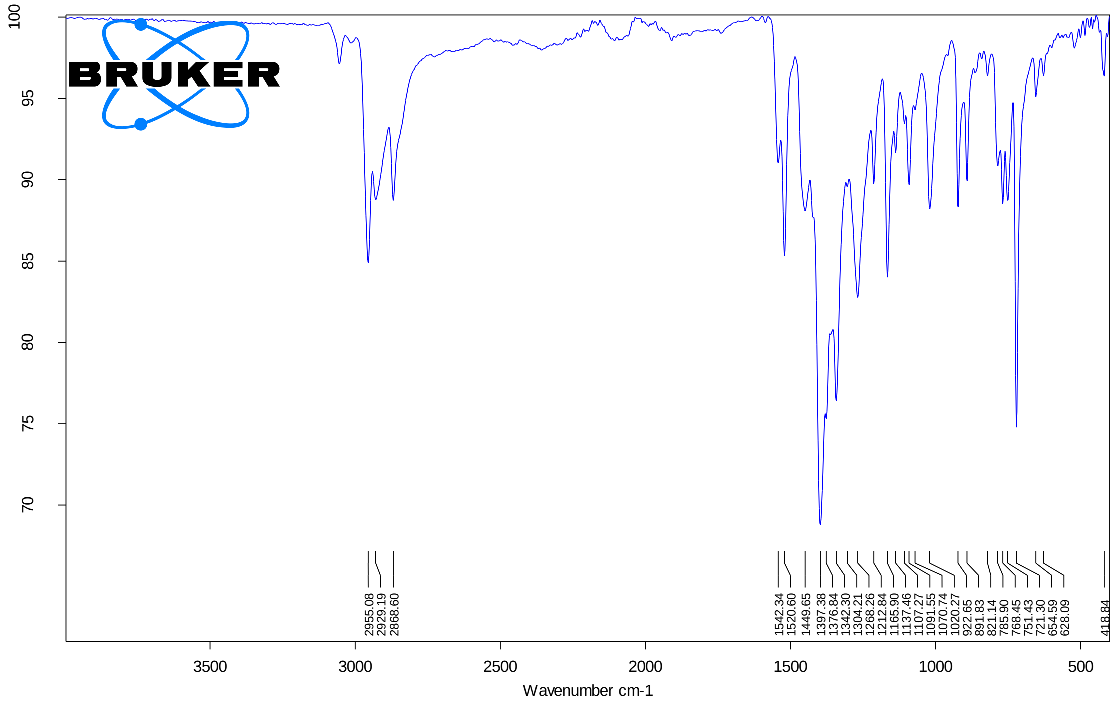

Figure S58. IR (ATR) spectrum of **4**

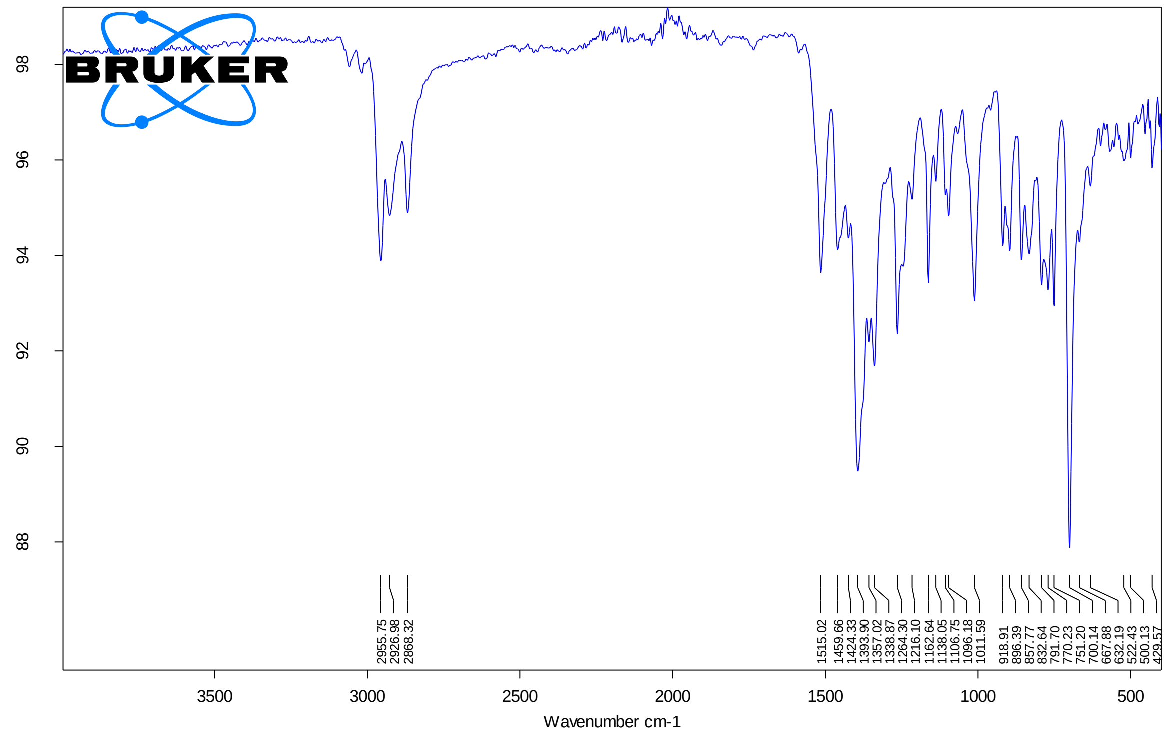

Figure S59. IR (ATR) spectrum of **5**

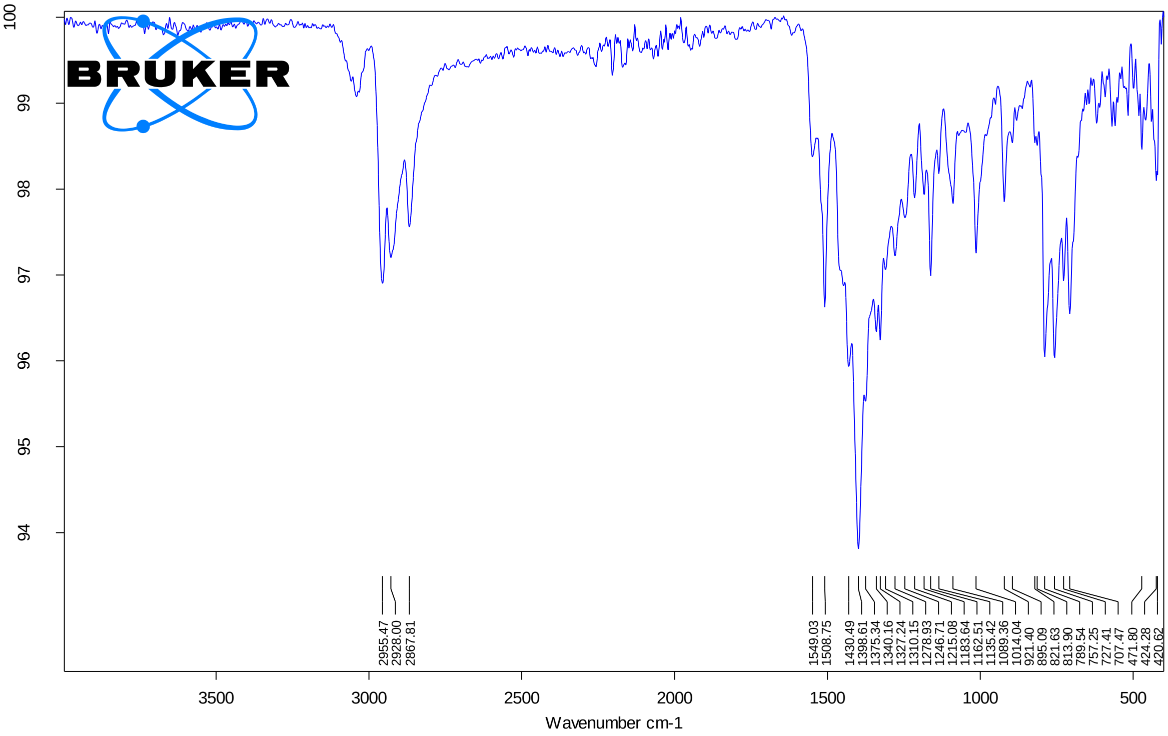

Figure S60. IR (ATR) spectrum of **6**

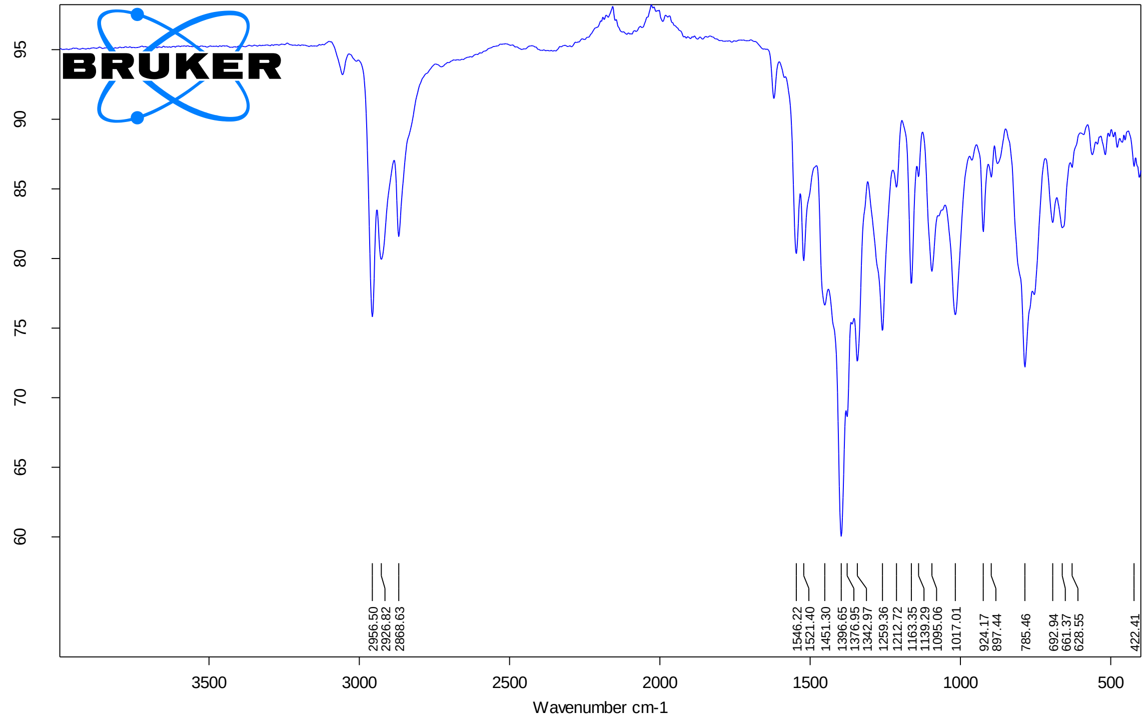

Figure S61. IR (ATR) spectrum of **7**

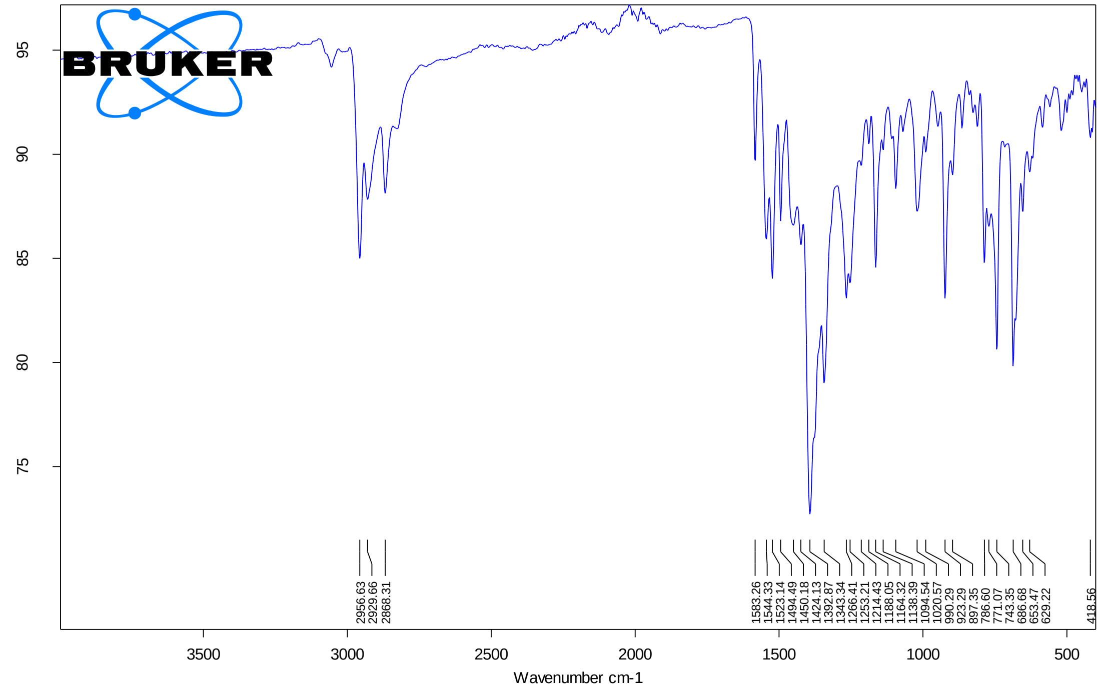

Figure S62. IR (ATR) spectrum of **8**

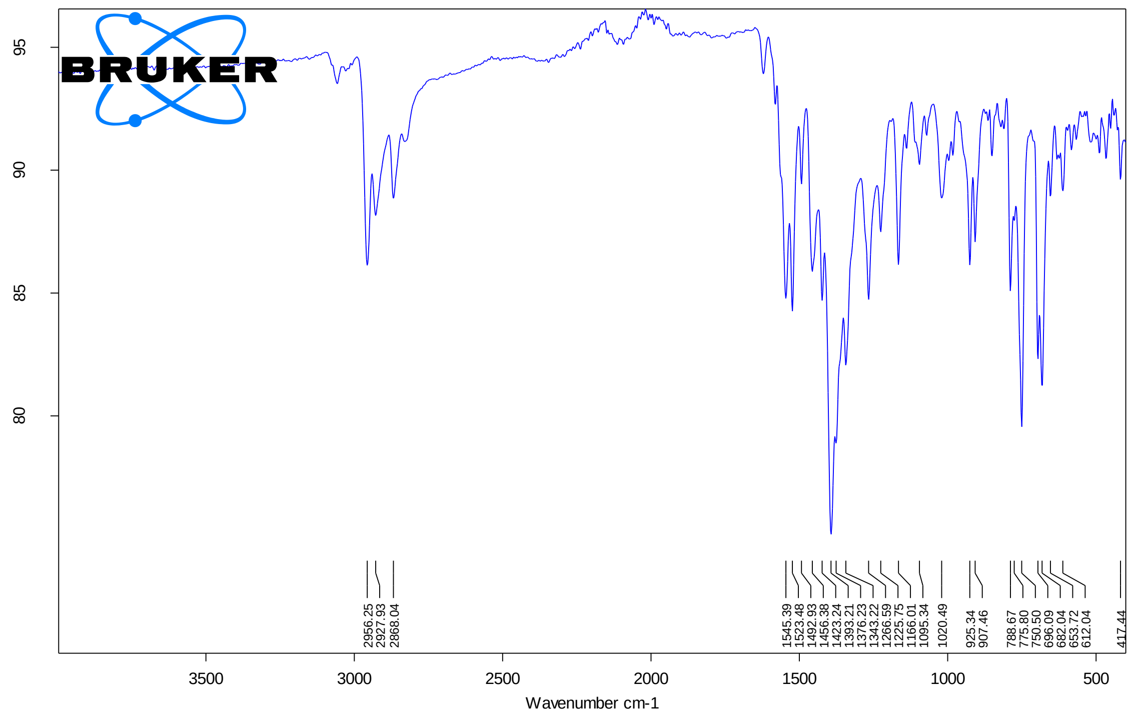

Figure S63. IR (ATR) spectrum of **9**

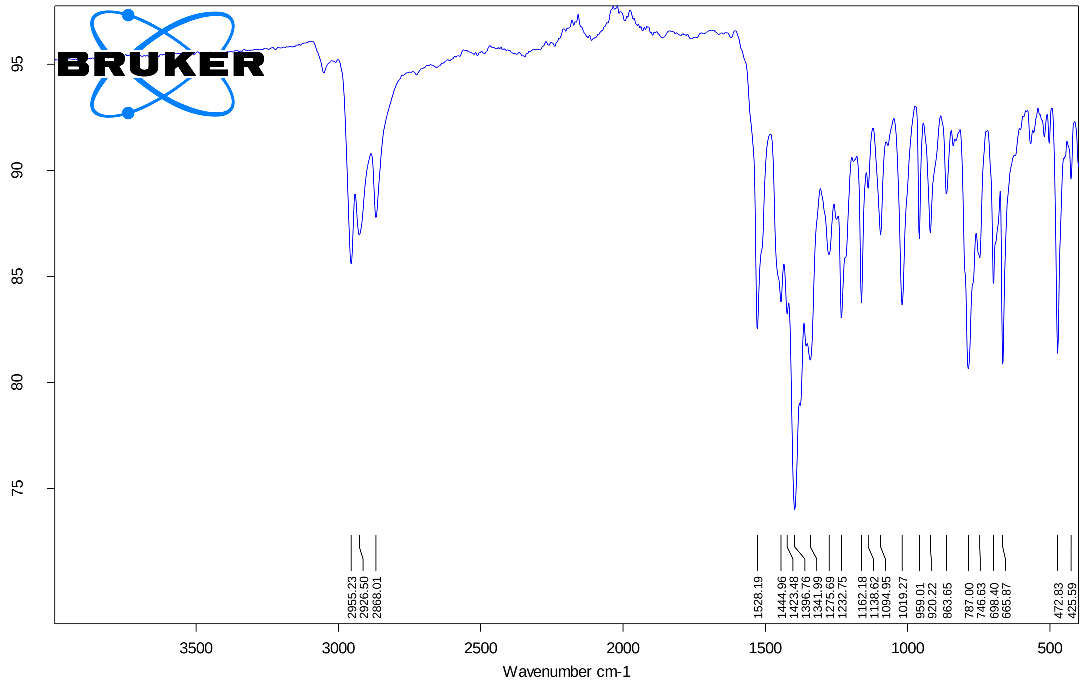

Figure S64. IR (ATR) spectrum of **10**

3c. UV-vis data

All complexes (**1-10**) are highly coloured and show strong absorptions in the UV/UV-vis regions. The broad intense band in the visible region is responsible for the extremely dark colour of their crystals and solutions; it may be attributed to ligand to metal charge transfer (LMCT) or an excitation of the ligand-based orbitals.

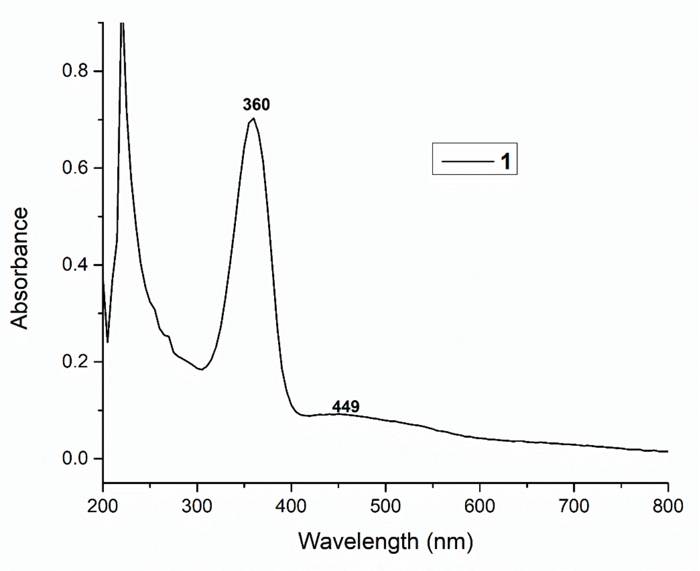

Figure S65. UV-vis data of complex **1** in cyclohexane at room temperature.

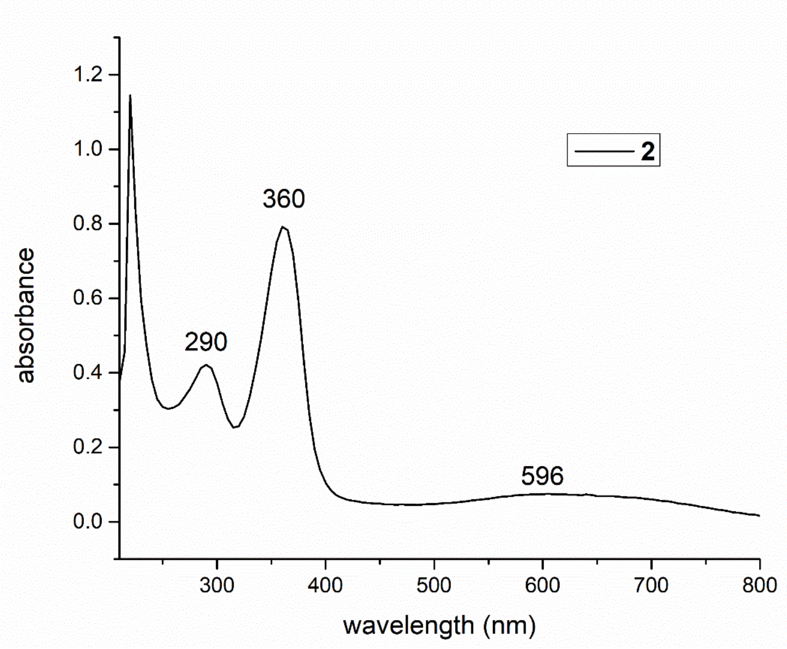

Figure S66. UV-vis data of complex **2** in cyclohexane at room temperature.

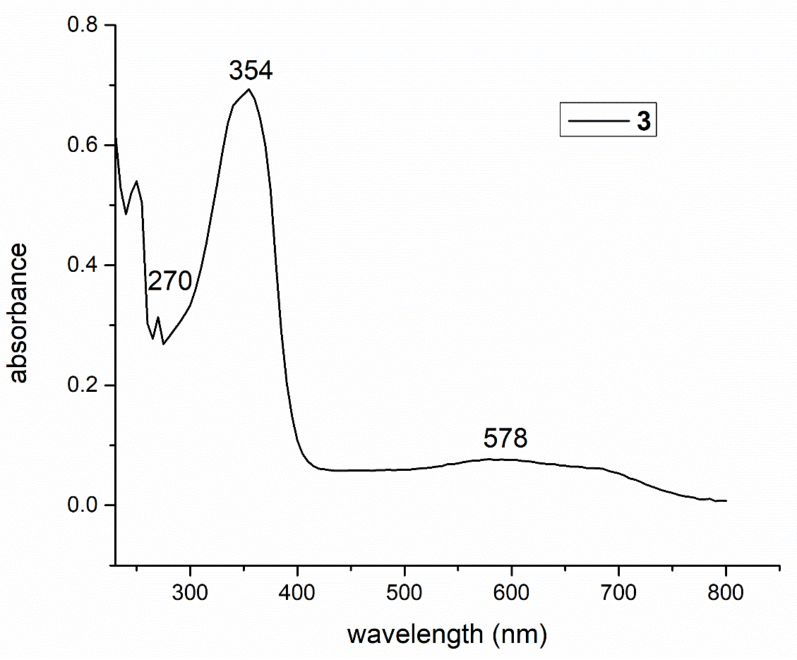

Figure S67. UV-vis data of complex **3** in cyclohexane at room temperature.

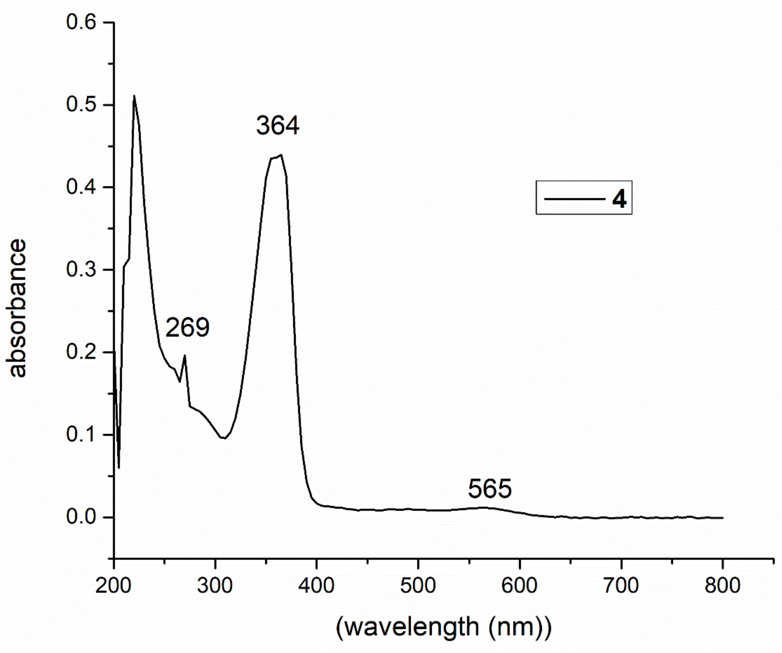

Figure S68. UV-vis data of complex **4** in cyclohexane at room temperature.

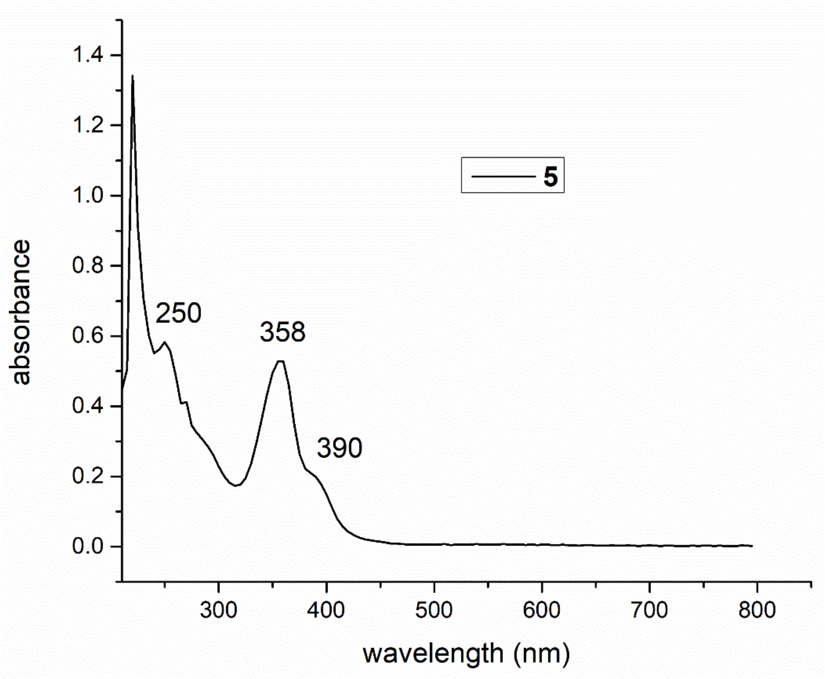

Figure S69. UV-vis data of complex **5** in cyclohexane at room temperature.

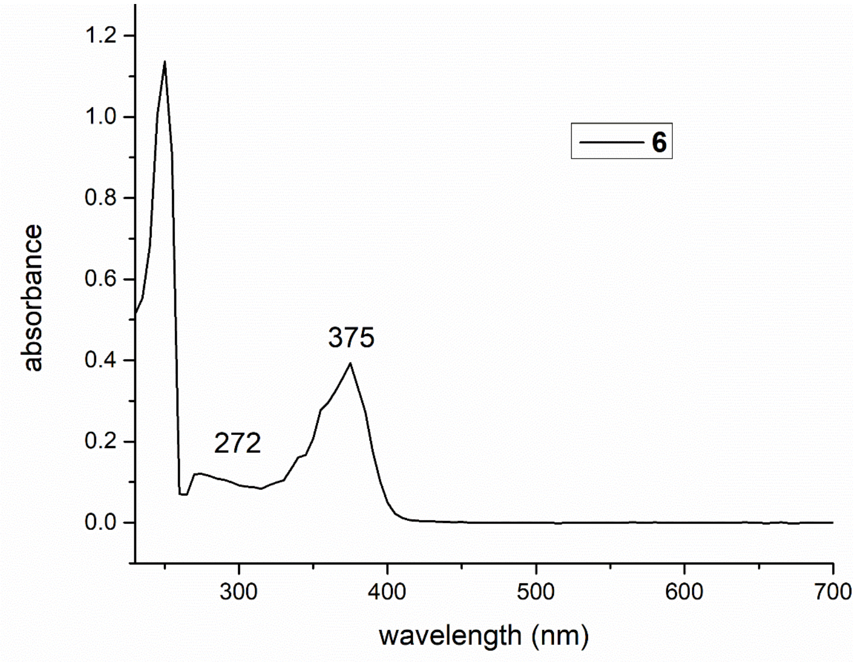

Figure S70. UV-vis data of complex **6** in cyclohexane at room temperature.

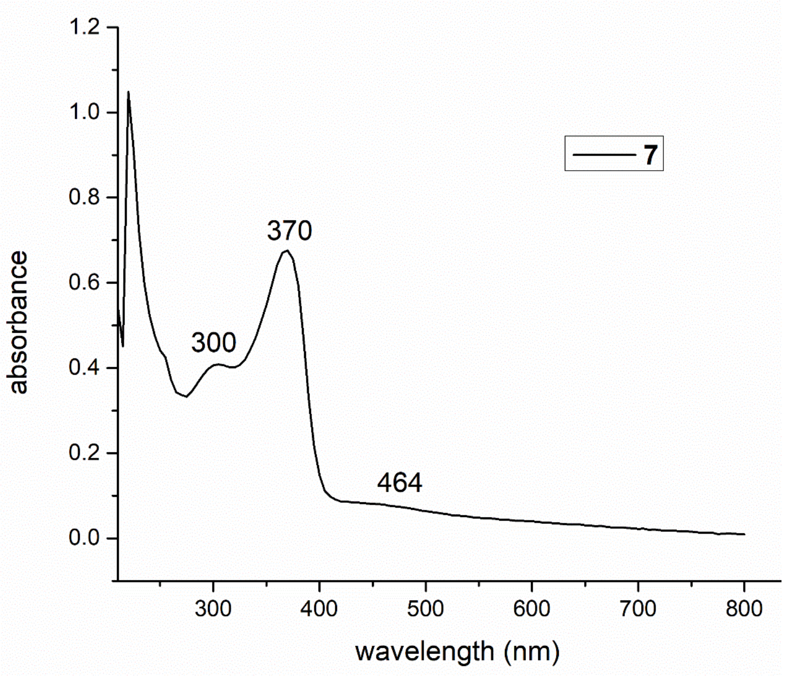

Figure S71. UV-vis data of complex **7** in cyclohexane at room temperature.

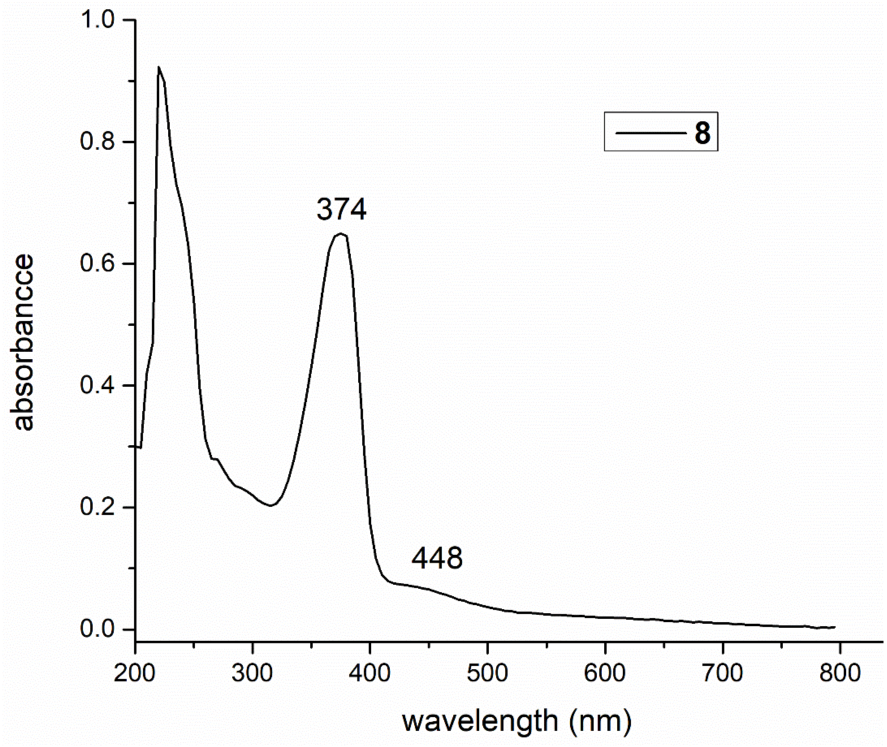

Figure S72. UV-vis data of complex **8** in cyclohexane at room temperature.

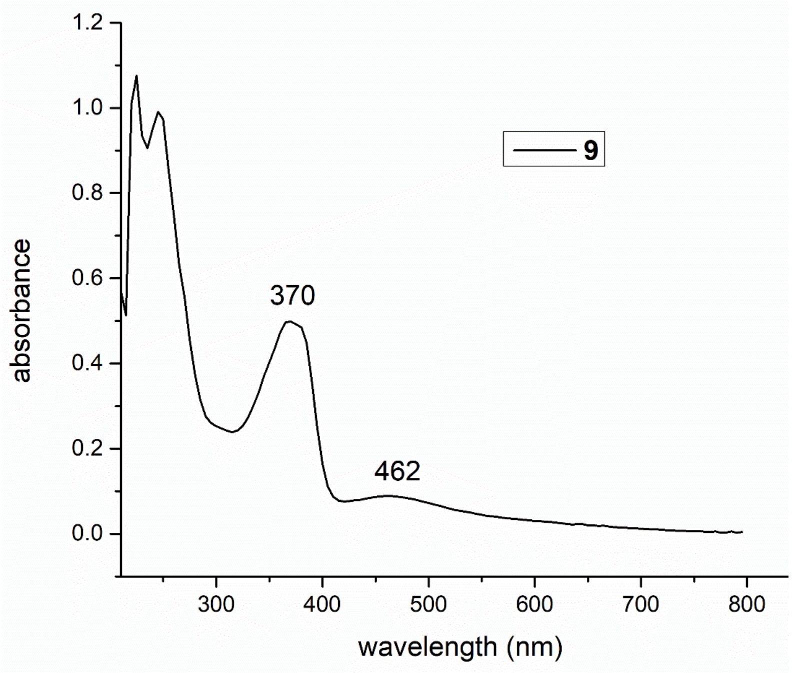

Figure S73. UV-vis data of complex **9** in cyclohexane at room temperature.

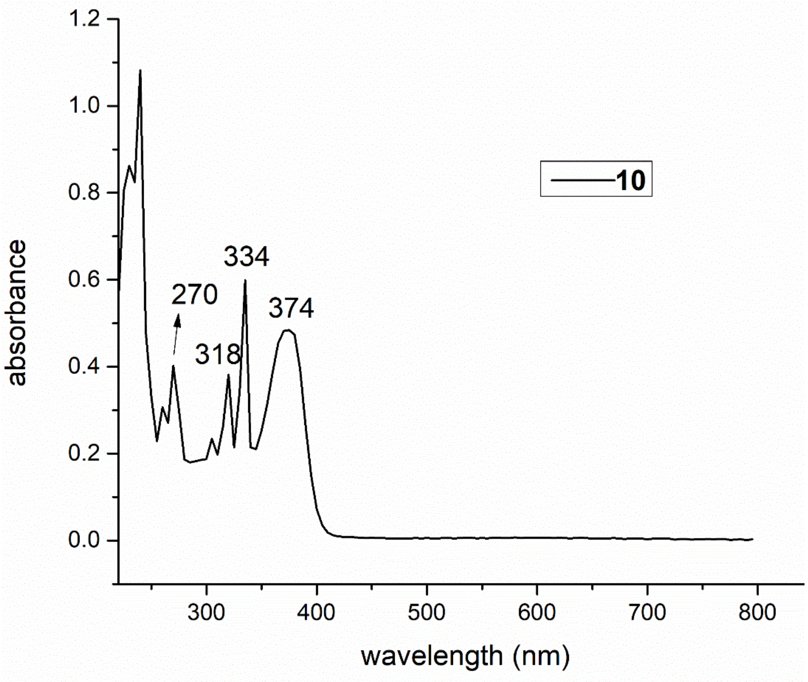

Figure S74. UV-vis data of complex **10** in cyclohexane at room temperature.

Table S2. UV-vis data of complexes **1-10**

Complex	Concentration (molar)	λ_{max} (nm)	ε ($Lmol^{-1}cm^{-1}$)
**1**	4.69×10^{-5}	360, 449	14929, 1971
**2**	0.99×10^{-5}	290, 360, 596	41773, 79348, 7403
**3**	1.01×10^{-5}	270, 354, 578	31220, 68511, 7635
**4**	1.05×10^{-5}	269, 364, 565	19106, 43291, 1201
**5**	1.94×10^{-5}	250, 358, 390	29538, 26767, 10299
**6**	9.09×10^{-6}	272, 375	12642, 41922
**7**	1.61×10^{-5}	300, 370, 464	25041, 41737, 4839
**8**	1.02×10^{-5}	374, 448	53730, 5252
**9**	1.03×10^{-5}	370, 462	49371, 9033
**10**	5.86×10^{-6}	270, 303, 318, 334, 374,	66133, 39410, 64497, 100249, 83131

4. Crystal structure determination

Suitable single crystals of compounds **1**-**10** were embedded in protective perfluoropolyalkylether oil (viscosity 1800 cSt; ABCR GmbH) on a microscope slide and a single specimen was selected and subsequently transferred to the cold nitrogen gas stream of the diffractometer.

The intensity data was collected at 100 K using using MoK_{α} radiation (λ = 0.71073 Å; compounds **2**, **8** and **9**) or CuK_{α} radiation (λ = 1.54184 Å, all other compounds) on an Agilent SuperNova dual radiation diffractometer with microfocus X-ray sources and mirror optics. The measured data were processed with the CrysAlisPro software package.[6] Data were corrected for Lorentz and polarization effects, and an empirical absorption correction using spherical harmonics was applied. In all cases except of compound **3** and **6**, an additional numerical absorption correction based on gaussian integration over a multifaceted crystal model was performed. For **6**, an additional analytical absorption correction[7] was performed instead. Using Olex2,[8] the structures were solved by dual-space methods (SHELXT)[9] and refined by full-matrix least-squares procedures on F^2 using SHELXL.[10] All non-hydrogen atoms were refined with anisotropic displacement parameters. Most H-atoms were placed in geometrically calculated positions and refined by using a riding model where each H-atom was assigned a fixed isotropic displacement parameter with a value equal to 1.2U_{eq} (CH or CH_2) or 1.5U_{eq} (CH_3) of its parent C-atom.

The data of compound **1** showed minor disorder of the THF ligand. The use of similarity restraints (SADI, SIMU) and rigid bond restraints (RIGU)[11] during refinement ensured reasonable geometries within the disordered part of the structure. Site occupancy factors of 0.878(14) and 0.122(14) were subsequently determined for the two alternative orientations of the THF ligand. The hydrogen atoms of the reduced biphenyl ligand were placed in the positions indicated by a difference electron density map and these positions were refined together with an isotropic displacement parameter.

The structure of compound **2** suffered from substantial disorder, which affected every atom except of ytterbium. This disorder was modeled with the help of similarity restraints (SADI, SIMU) and rigid bond restraints (RIGU).[11] The relative occupancies of the two alternative orientations of the disordered parts of the compound were refined to 0.549(7)/0.451(7) (naphthalene), 0.758(5)/0.242(5) (THP) and 0.694(3)/0.306(5) (BDI ligand), respectively.

In case of compound **3**, the hydrogen atoms of the reduced anthracene ligand were placed in the positions indicated by a difference electron density map and these positions were refined together with an isotropic displacement parameter. The hydrogen atom H3 in the ligand backbone deviated significantly from the position calculated *via* the riding model and its position was therefore refined freely as well, but the value of its isotropic displacement parameter was kept equal to $1.2U_{\mathrm{eq}}$ of its parent C-atom. Disorder of the ether ligand, of an ethyl group and of co-crystallized *n*-pentane was observed and modeled with similarity restraints (SADI, SIMU) and rigid bond restraints (RIGU).[11] Site occupancy factors of 0.840(6)/0.160(6) (diethyl ether) and 0.71(5)/0.29(5) (Et) were later on determined, while the site occupancy factor for *n*-pentane, which was disordered about an inversion center, was constrained to 0.5.

The crystal of compound **4** was twinned by pseudo-merohedry (twin law 1, 0, 1 / 0, -1, 0 / 0, 0, -1). The contributions of the two twin domains were later on refined to 0.5057(10) and 0.4943(10). The asymmetric unit contained two molecules of the compound, which were partially disordered. The disorder affected a complete BDI ligand and an ethyl group in molecule 1, and three dipep substituents in molecule 2. The use of geometry restraints (FLAT), similarity restraints (SADI, SIMU) and rigid bond restraints (RIGU)[11] was necessary to build a chemically reasonable model of the disorder. The relative occupancies of the two alternative orientations of the disordered moieties were refined to 0.575(5)/0.425(5) (BDI ligand), 0.630(12)/0.370(12) (Et), 0.503(6)/0.497(6) (dipep 1), 0.517(6)/0.483(6) (dipep 2) and 0.698(8)/0.302(8) (dipep 3), respectively.

The structure of compound **5** suffered from disorder as well. Here, the THF ligand, a N-dipep moiety, and two additional 3-pentyl groups were affected. The related site occupancy factors were 0.558(10)/0.442810) (THF), 0.570(4)/0.430(4) (N-dipep), 0.524(9)/0.476(9) (3-pentyl 1) and 0.618(6)/0.382(6), respectively. During the refinement, similarity restraints (SADI, SIMU) and rigid bond restraints (RIGU)[11] were applied.

In case of compound **6**, the hydrogen atoms of the reduced anthracene ligand were placed in the positions indicated by a difference electron density map and these positions were refined together with an isotropic displacement parameter. Disorder of the 2-Me-THF ligand and of an ethyl group was observed and modeled with similarity restraints (SADI, SIMU) and rigid bond restraints (RIGU).[11] Site occupancy factors of 0.818(9) and 0.182(9) were later on determined for the two altnative orientations of both moieties. The co-crystallized cyclohexane was severely disordered as well. Its contribution to the structure factors was secured by back-Fourier transformation using the solvent mask routine[12] of the program Olex2.[9] The solvent accessible voids treated this way had a size of 325.8 Å^3 (7.1% of the unit cell) and contained 91.0 electrons. This corresponded to 2 cyclohexane per unit cell (96 electrons).

The THF ligand in complex **7** was disordered as well. The relative occupancies of its two alternative orientations were refined to 0.627(18) and 0.373(18). The hydrogen atoms of the C_6H_5 moiety of the reduced toluene ligand were placed in the positions indicated by a difference electron density map and these positions were refined together with an isotropic displacement parameter.

Crystals of compound **8**, grown from a mixture of pentane and THF, contained one molecule of **8** and two disordered solvent molecules per asymmetric unit. While the first solvent molecule clearly was a disordered *n*-pentane, the second solvent site contained a mixture of *n*-pentane and minor amounts of THF. The related site occupancy factors were 0.759(7)/0.241(7) (*n*-pentane) and 0.815(5)/0.185(5) (*n*-pentane/THF). During the refinement, similarity restraints (SADI, SIMU) and geometry restraints (DANG, DFIX) were used.

Co-crystallized solvent was also present in crystals of **9**, obtained from hexanes (isomeric mixture). While the first solvent site was occupied by *n*-hexane, the second one contained a mixture of different hexane isomers, with 3-methylpentane as the major occupant. Since no decent disorder model was found, the contribution of this second solvent site to the structure factors was secured by back-Fourier transformation using the solvent mask routine[12] of the program Olex2.[9] The solvent accessible voids

treated this way had a size of 1169.8 Å^3 (12.0% of the unit cell) and contained 196.8 electrons. One of the dipep substituents of **9** was disordered. This disorder was modeled with the help of similarity restraints (SADI, SIMU) and rigid bond restraints (RIGU).[11] The relative occupancies of the two alternative orientations were refined to 0.603(8) and 0.397(8). The hydrogen atoms of the coordinated and reduced phenyl moiety were placed in the positions indicated by a difference electron density map and these positions were refined together with an isotropic displacement parameter.

The asymmetric unit of **10** contained two symmetry-independent half-molecules. While half-molecule 1 was perfectly ordered, the THF ligand of half-molecule 2 had two alternative orientations. During refinement of this disorder, similarity restraints (SADI, SIMU) and rigid bond restraints (RIGU)[11] were applied. The resulting site occupancy factors were 0.720(18) and 0.280(18). The hydrogen atoms of the reduced pyrene ligands were placed in the positions indicated by a difference electron density map and these positions were refined together with an isotropic displacement parameter. The corresponding C-H bond lengths were restrained to be similar (SADI) in both half-molecules.

Crystallographic and refinement data are summarized in Table S3-6.

Table S3. Crystal data and structure refinement for compounds **1**-**3**.

Compound	Compound **1**	Compound **2**	Compound **3**·*n*-pentane
Identification code	hasj250522a	hasj240626b	hasj240516a
Empirical formula	$C_{94}H_{140}N_4O_2Yb_2$	$C_{94}H_{142}N_4O_2Yb_2$	$C_{101}H_{156}N_4O_2Yb_2$
Formula weight	1704.17	1706.19	1804.37
Temperature/K	100.00(10)	100.0(2)	99.97(16)
Crystal system	triclinic	triclinic	monoclinic
Space group	*P*-1	*P*-1	$P2_1/n$
a/Å	12.9066(4)	12.6233(3)	18.0228(3)
b/Å	12.9725(4)	13.0415(3)	13.7648(2)
c/Å	14.4672(4)	15.1045(3)	19.3726(4)
α/°	79.367(2)	95.548(2)	90
β/°	83.551(2)	104.228(2)	108.7068(19)
γ/°	60.711(3)	115.185(2)	90
Volume/Å^3	2075.64(12)	2123.00(9)	4552.08(15)
Z	1	1	2
ρ_{calc}/g/cm^3	1.363	1.335	1.316
μ/mm^{-1}	4.425	2.238	4.063
F(000)	888.0	890.0	1892.0
Crystal size/mm^3	0.15 × 0.126 × 0.005	0.186 × 0.131 × 0.066	0.202 × 0.091 × 0.009
Radiation	Cu Kα (λ = 1.54184)	Mo Kα (λ = 0.71073)	Cu Kα (λ = 1.54184)
2θ range for data collection/°	7.856 to 143.648	4.502 to 58.916	8.03 to 144.806
Index ranges	-15 ≤ h ≤ 15, -15 ≤ k ≤ 13, -17 ≤ l ≤ 17	-17 ≤ h ≤ 17, -17 ≤ k ≤ 16, -20 ≤ l ≤ 18	-21 ≤ h ≤ 22, -16 ≤ k ≤ 12, -22 ≤ l ≤ 23
Reflections collected	26505	47519	25808
Independent reflections	7922 [R_{int} = 0.0475, R_{sigma} = 0.0437]	10609 [R_{int} = 0.0482, R_{sigma} = 0.0485]	8806 [R_{int} = 0.0401, R_{sigma} = 0.0409]
Data/restraints/parameters	7922/251/527	10609/6017/933	8806/251/610
Goodness-of-fit on F^2	1.028	1.062	1.037
Final R indexes [I>=2σ (I)]	R_1 = 0.0314, wR_2 = 0.0769	R_1 = 0.0350, wR_2 = 0.0617	R_1 = 0.0349, wR_2 = 0.0869
Final R indexes [all data]	R_1 = 0.0358, wR_2 = 0.0795	R_1 = 0.0491, wR_2 = 0.0659	R_1 = 0.0426, wR_2 = 0.0919
Largest diff. peak/hole / e Å^{-3}	1.81/-1.12	1.46/-1.06	1.76/-1.09
CCDC number	2454435	2454436	2454437

Table S4. Crystal data and structure refinement for compounds **4**-**6**.

Compound	Compound **4**	Compound **5**	Compound **6**·Cyclohexane
Identification code	hasj231023c	hasj240119b	hasj250801a
Empirical formula	$C_{82}H_{122}N_4Yb_2$	$C_{49}H_{73}N_2OSm$	$C_{104}H_{156}N_4O_2Sm_2$
Formula weight	1509.91	856.44	1795.02
Temperature/K	99.98(10)	100.0(3)	100.00(10)
Crystal system	monoclinic	monoclinic	monoclinic
Space group	$P2_1/c$	$P2_1/n$	$P2_1/n$
a/Å	29.0514(4)	13.5490(2)	18.1522(11)
b/Å	22.0120(2)	23.1836(3)	13.9234(8)
c/Å	26.0740(4)	14.6662(2)	19.2025(13)
α/°	90	90	90
β/°	116.661(2)	106.3980(10)	108.012(7)
γ/°	90	90	90
Volume/Å^3	14901.0(4)	4419.48(11)	4615.4(5)
Z	8	4	2
ρ_{calc}/g/cm^3	1.346	1.287	1.292
μ/mm^{-1}	4.840	10.237	9.826
F(000)	6256.0	1804.0	1896.0
Crystal size/mm^3	0.313 × 0.26 × 0.125	0.323 × 0.195 × 0.029	0.256 × 0.214 × 0.016
Radiation	Cu Kα (λ = 1.54184)	Cu Kα (λ = 1.54184)	Cu Kα (λ = 1.54184)
2θ range for data collection/°	6.78 to 145.032	7.35 to 145.278	7.986 to 143.684
Index ranges	-35 ≤ h ≤ 34, -25 ≤ k ≤ 26, -31 ≤ l ≤ 32	-16 ≤ h ≤ 16, -26 ≤ k ≤ 27, -18 ≤ l ≤ 18	-22 ≤ h ≤ 20, -16 ≤ k ≤ 11, -23 ≤ l ≤ 21
Reflections collected	115182	41683	18290
Independent reflections	29002 [R_{int} = 0.0438, R_{sigma} = 0.0348]	8650 [R_{int} = 0.0445, R_{sigma} = 0.0299]	8760 [R_{int} = 0.0610, R_{sigma} = 0.0779]
Data/restraints/parameters	29002/11748/2436	8650/1967/779	8760/425/574
Goodness-of-fit on F^2	1.025	1.017	1.046
Final R indexes [I>=2σ (I)]	R_1 = 0.0416, wR_2 = 0.1027	R_1 = 0.0318, wR_2 = 0.0765	R_1 = 0.0597, wR_2 = 0.1584
Final R indexes [all data]	R_1 = 0.0492, wR_2 = 0.1088	R_1 = 0.0392, wR_2 = 0.0812	R_1 = 0.0779, wR_2 = 0.1725
Largest diff. peak/hole / e Å^{-3}	2.24/-1.46	0.73/-0.74	1.10/-1.83
CCDC number	2454438	2454439	2481810

Table S5. Crystal data and structure refinement for compounds **7**-**9**.

Compound	Compound **7**	Compound **8**·1.815(*n*-pentane)·0.185(THF)	Compound **9**·*n*-hexane·hexane
Identification code	hasj240123b	hasj240205b	hasj231122a
Empirical formula	$C_{89}H_{138}N_4O_2Sm_2$	$C_{99.81}H_{155.26}N_4O_{1.19}Sm_2$	$C_{110}H_{160}N_4Sm_2$
Formula weight	1596.73	1730.97	1839.11
Temperature/K	99.99(10)	99.97(15)	99.98(10)
Crystal system	monoclinic	triclinic	monoclinic
Space group	$P2_1/c$	*P*-1	$P2_1/c$
a/Å	19.1538(2)	14.2111(3)	23.2368(4)
b/Å	19.2123(2)	17.8935(4)	21.0517(4)
c/Å	22.5678(2)	19.1462(4)	20.8273(4)
α/°	90	80.0854(18)	90
β/°	101.0450(10)	89.4096(16)	106.711(2)
γ/°	90	70.8174(19)	90
Volume/Å^3	8150.86(14)	4524.00(17)	9757.9(3)
Z	4	2	4
ρ_{calc}/g/cm^3	1.301	1.271	1.252
μ/mm^{-1}	11.061	1.333	1.240
F(000)	3360.0	1831.0	3888.0
Crystal size/mm^3	0.272 × 0.131 × 0.103	0.243 × 0.159 × 0.028	0.341 × 0.26 × 0.102
Radiation	Cu Kα (λ = 1.54184)	Mo Kα (λ = 0.71073)	Mo Kα (λ = 0.71073)
2θ range for data collection/°	7.212 to 145.226	4.324 to 56.562	3.87 to 58.964
Index ranges	-23 ≤ h ≤ 22, -23 ≤ k ≤ 22, -27 ≤ l ≤ 27	-18 ≤ h ≤ 18, -18 ≤ k ≤ 22, -24 ≤ l ≤ 25	-31 ≤ h ≤ 31, -27 ≤ k ≤ 28, -28 ≤ l ≤ 28
Reflections collected	61965	40541	110648
Independent reflections	15896 [R_{int} = 0.0464, R_{sigma} = 0.0368]	20526 [R_{int} = 0.0345, R_{sigma} = 0.0661]	24641 [R_{int} = 0.0360, R_{sigma} = 0.0359]
Data/restraints/parameters	15896/0/934	20526/574/1082	24641/1108/1182
Goodness-of-fit on F^2	1.018	1.045	1.074
Final R indexes [I>=2σ (I)]	R_1 = 0.0298, wR_2 = 0.0711	R_1 = 0.0424, wR_2 = 0.0800	R_1 = 0.0334, wR_2 = 0.0667
Final R indexes [all data]	R_1 = 0.0352, wR_2 = 0.0744	R_1 = 0.0691, wR_2 = 0.0902	R_1 = 0.0466, wR_2 = 0.0717
Largest diff. peak/hole / e Å^{-3}	0.58/-0.91	1.62/-0.84	1.14/-0.68
CCDC number	2454440	2454441	2454442

Table S6. Crystal data and structure refinement for compound **10**.

Compound	Compound **10**
Identification code	hasj240201b
Empirical formula	$C_{201}H_{292}N_8O_4Sm_4$
Formula weight	3485.81
Temperature/K	99.97(15)
Crystal system	triclinic
Space group	*P*-1
a/Å	14.2805(7)
b/Å	18.6260(12)
c/Å	19.4125(11)
α/°	61.779(6)
β/°	78.252(4)
γ/°	85.582(4)
Volume/Å^3	4453.3(5)
Z	1
ρ_{calc}/g/cm^3	1.300
μ/mm^{-1}	10.169
F(000)	1834.0
Crystal size/mm^3	0.146 × 0.096 × 0.026
Radiation	Cu Kα (λ = 1.54184)
2θ range for data collection/°	7.418 to 145.14
Index ranges	-15 ≤ h ≤ 17, -22 ≤ k ≤ 22, -22 ≤ l ≤ 23
Reflections collected	49378
Independent reflections	17154 [R_{int} = 0.0639, R_{sigma} = 0.0682]
Data/restraints/parameters	17154/89/1063
Goodness-of-fit on F^2	1.022
Final R indexes [I>=2σ (I)]	R_1 = 0.0474, wR_2 = 0.1172
Final R indexes [all data]	R_1 = 0.0598, wR_2 = 0.1267
Largest diff. peak/hole / e Å^{-3}	1.20/-1.71
CCDC number	2454443

5. References

[1] S. Meiries, G. Le Duc, A. Chartoire, A. Collado, K. Speck, K. S. A. Arachchige, A. M. Z. Slawin, S. P. Nolan, *Chem. Eur. J.* **2013**, *19*, 17358–17368.

[2] S. K. Thakur, N. Roig, R. Monreal-Corona, J. Langer, M. Alonso, S. Harder, Similarities and Differences in Benzene Reduction with Ca, Sr, Yb and Sm: Strong Evidence for Tetra-Anionic Benzene. *Angew. Chem. Int. Ed.* **2024**, *63* (25), e202405229.

[3] G. M. Richardson, I. Douair, S. A. Cameron, L. Maron, M. D. Anker, "Ytterbium (II) Hydride as a Powerful Multielectron Reductant" *Chem.– Eur. J.* **2021**, *27*, 13144–13148.

[4] K. Meerholz, J. Heinze, Multiple Reversible Electrochemical Reduction of Aromatic Hydrocarbons in Liquid Alkylamines. *J. Am. Chem. Soc.* **1989**, *111*, 2325-2326.

[5] A. Dahlén, Å. Nilsson, G. Hilmersson, Estimating the Limiting Reducing Power of SmI_2/H_2O/Amine and YbI_2/H_2O/Amine by Efficient Reduction of Unsaturated Hydrocarbons. *J. Org. Chem.* **2006**, *71*, 1576-1580.

[6] Rigaku Oxford Diffraction, **2022**, CrysAlisPro Software system, version 1.171.42.72a, Rigaku Corporation, Wroclaw, Poland (compounds **4** and **8**); (b) Rigaku Oxford Diffraction, **2024**, CrysAlisPro Software system, version 1.171.43.106a, Rigaku Corporation, Wroclaw, Poland (all other compounds).

[7] R. C. Clark, J. S. Reid, The analytical calculation of absorption in multifaceted crystals. *Acta Crystallogr., Sect. A: Found. Crystallogr.* **1995**, *51*, 887-897.

[8] O. V. Dolomanov, L. J. Bourhis, R.J. Gildea, J. A. K. Howard, H. Puschmann, OLEX2: a complete structure solution, refinement and analysis program. *J. Appl. Cryst.* **2009**, *42*, 339–341.

[9] G. M. Sheldrick, SHELXT–Integrated space-group and crystal-structure determination. *Acta Crystallogr., Sect. A: Found. Adv.* **2015**, *71*, 3–8.

[10] G. M. Sheldrick, Crystal structure refinement with SHELXL. *Acta Crystallogr., Sect. C: Struct. Chem.* **2015**, *71*, 3–8.

[11] A. Thorn, B. Dittrich, G. M. Sheldrick, Enhanced rigid-bond restraints. *Acta Crystallogr., Sect. A: Found. Crystallogr.* **2012**, *68*, 448–451.

[12] P. Van der Sluis, A. L. Spek, BYPASS: an Effective Method for the Refinement of Crystal Structures Containing Disordered Solvent Regions. *Acta Crystallogr., Sect. A: Found. Crystallogr.* **1990**, *46*, 194-201.
