# Supplementary material for: Syntheses and Reactivity of Yb and Sm Inverse Sandwich Arene Complexes
Source: Chemistry. 2025 Oct 8;31(62):e02710. doi: 10.1002/chem.202502710 (PMC12598387; doi:10.1002/chem.202502710)

checkCIF/PLATON report

Structure factors have been supplied for datablock(s) hasj240626b

THIS REPORT IS FOR GUIDANCE ONLY. IF USED AS PART OF A REVIEW PROCEDURE FOR PUBLICATION, IT SHOULD NOT REPLACE THE EXPERTISE OF AN EXPERIENCED CRYSTALLOGRAPHIC REFEREE.

No syntax errors found. CIF dictionary Interpreting this report

Datablock: hasj240626b

Bond precision:	= 0.0000 A		Wavelength=0.71073
Cell:	a=12.6233(3)	b=13.0415(3)	c=15.1045(3)
	alpha=95.548(2)	beta=104.228(2)	gamma=115.185(2)
Temperature:	100 K		

	Calculated	Reported
Volume	2123.01(10)	2123.00(9)
Space group	P -1	P -1
Hall group	-P 1	-P 1
Moiety formula	C94 H142 N4 O2 Yb2	C94 H142 N4 O2 Yb2
Sum formula	C94 H142 N4 O2 Yb2	C94 H142 N4 O2 Yb2
Mr	1706.19	1706.19
Dx,g cm-3	1.334	1.335
Z	1	1
Mu (mm-1)	2.238	2.238
F000	890.0	890.0
F000'	889.48	
h,k,lmax	17,18,20	17,17,20
Nref	11762	10609
Tmin,Tmax	0.710,0.863	0.692,1.000
Tmin'	0.653	

Correction method= # Reported T Limits: Tmin=0.692 Tmax=1.000
AbsCorr = GAUSSIAN

Data completeness= 0.902 Theta(max)= 29.458

R(reflections)= 0.0350(8900) wR2(reflections)= 0.0659(10609)

S = 1.062 Npar= 933

The following ALERTS were generated. Each ALERT has the format
test-name_ALERT_alert-type_alert-level.
Click on the hyperlinks for more details of the test.

Alert level C

```
PLAT220_ALERT_2_C NonSolvent   Resd 1  C   Ueq(max)/Ueq(min) Range        3.1 Ratio
PLAT910_ALERT_3_C Missing # of FCF Reflection(s) Below Theta(Min).          6 Note
                1  0  0,  -1  1  0,   0  1  0,   0 -1  1,  -1  0  1,   0  0  1,
PLAT973_ALERT_2_C Check Calcd Positive Resid. Density on       Yb1       1.12 eA-3
```

Alert level G

```
PLAT002_ALERT_2_G Number of Distance or Angle Restraints on AtSite        101 Note
PLAT003_ALERT_2_G Number of Uiso or U(i,j)  Restrained non-H-Atoms        100 Report
PLAT154_ALERT_1_G The s.u.'s on the Cell Angles are Equal ..(Note)      0.002 Degree
PLAT176_ALERT_4_G The CIF-Embedded .res File Contains SADI Records         21 Report
PLAT178_ALERT_4_G The CIF-Embedded .res File Contains SIMU Records          3 Report
PLAT187_ALERT_4_G The CIF-Embedded .res File Contains RIGU Records          2 Report
PLAT188_ALERT_3_G A Non-default SIMU Restraint Value has been used     0.0200 Report
PLAT188_ALERT_3_G A Non-default SIMU Restraint Value has been used     0.0300 Report
PLAT188_ALERT_3_G A Non-default SIMU Restraint Value has been used     0.0200 Report
PLAT230_ALERT_2_G Hirshfeld Test Diff for    C20      --C21      .        6.4 s.u.
PLAT301_ALERT_3_G Main Residue  Disorder ..............(Resd    1)        98% Note
PLAT720_ALERT_4_G Number of Unusual/Non-Standard Labels ..........          6 Note
              H1AA    H1AB    H1AC    H5AA    H5AB    H5AC
PLAT811_ALERT_5_G No ADDSYM Analysis: Too Many Excluded Atoms ....          ! Info
PLAT860_ALERT_3_G Number of Least-Squares Restraints .............       6017 Note
PLAT912_ALERT_4_G Missing # of FCF Reflections Above STh/L=  0.600       1141 Note
PLAT941_ALERT_3_G Average HKL Measurement Multiplicity ...........        4.5 Low
PLAT969_ALERT_5_G The 'Henn et al.' R-Factor-gap value ...........      1.954 Note
              Predicted wR2: Based on SigI**2  3.38 or SHELX Weight  6.21
```

0 **ALERT level A** = Most likely a serious problem - resolve or explain
0 **ALERT level B** = A potentially serious problem, consider carefully
3 **ALERT level C** = Check. Ensure it is not caused by an omission or oversight
17 **ALERT level G** = General information/check it is not something unexpected

1 ALERT type 1 CIF construction/syntax error, inconsistent or missing data
5 ALERT type 2 Indicator that the structure model may be wrong or deficient
7 ALERT type 3 Indicator that the structure quality may be low
5 ALERT type 4 Improvement, methodology, query or suggestion
2 ALERT type 5 Informative message, check

It is advisable to attempt to resolve as many as possible of the alerts in all categories. Often the minor alerts point to easily fixed oversights, errors and omissions in your CIF or refinement strategy, so attention to these fine details can be worthwhile. In order to resolve some of the more serious problems it may be necessary to carry out additional measurements or structure refinements. However, the purpose of your study may justify the reported deviations and the more serious of these should normally be commented upon in the discussion or experimental section of a paper or in the "special_details" fields of the CIF. checkCIF was carefully designed to identify outliers and unusual parameters, but every test has its limitations and alerts that are not important in a particular case may appear. Conversely, the absence of alerts does not guarantee there are no aspects of the results needing attention. It is up to the individual to critically assess their own results and, if necessary, seek expert advice.

Publication of your CIF in IUCr journals

A basic structural check has been run on your CIF. These basic checks will be run on all CIFs submitted for publication in IUCr journals (*Acta Crystallographica*, *Journal of Applied Crystallography*, *Journal of Synchrotron Radiation*); however, if you intend to submit to *Acta Crystallographica Section C* or *E* or *IUCrData*, you should make sure that full publication checks are run on the final version of your CIF prior to submission.

Publication of your CIF in other journals

Please refer to the *Notes for Authors* of the relevant journal for any special instructions relating to CIF submission.

PLATON version of 02/02/2025; check.def file version of 02/02/2025

Datablock hasj240626b - ellipsoid plot

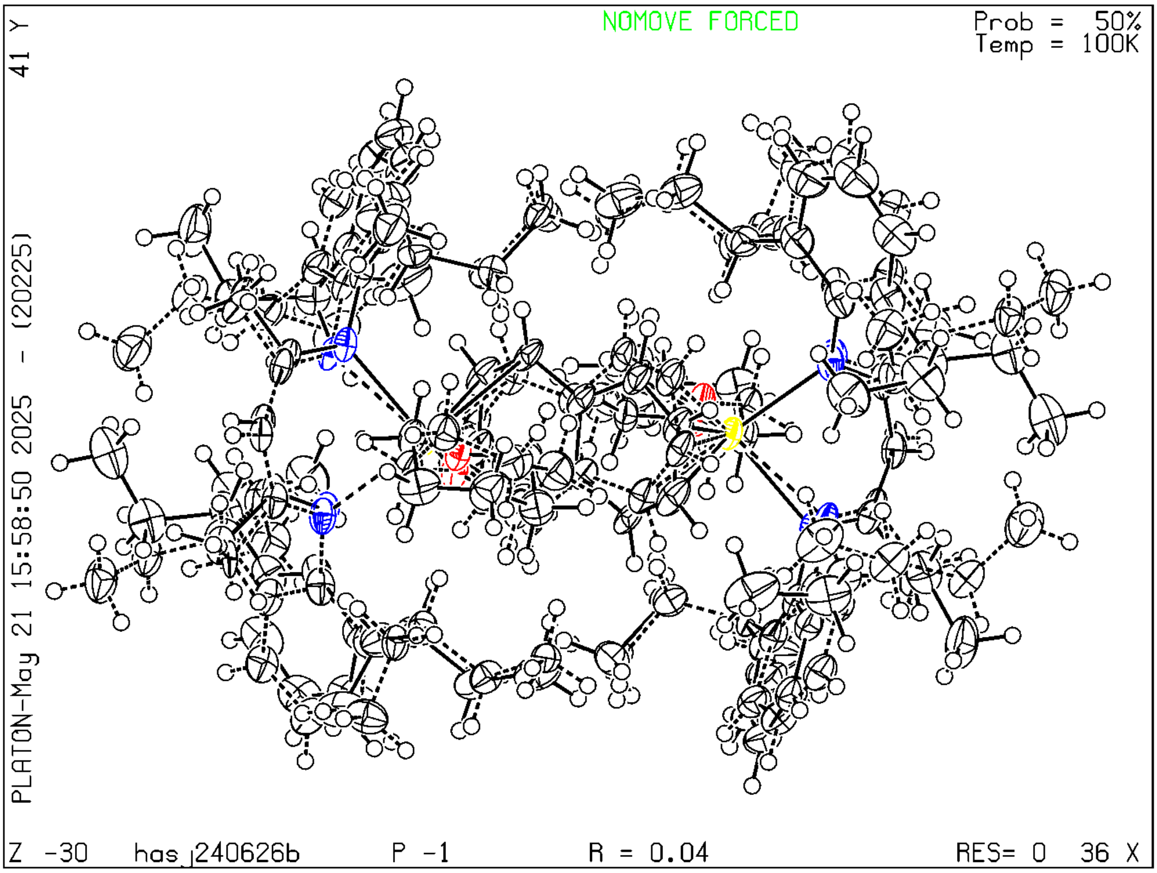

checkCIF/PLATON report

Structure factors have been supplied for datablock(s) hasj240516a

THIS REPORT IS FOR GUIDANCE ONLY. IF USED AS PART OF A REVIEW PROCEDURE FOR PUBLICATION, IT SHOULD NOT REPLACE THE EXPERTISE OF AN EXPERIENCED CRYSTALLOGRAPHIC REFEREE.

No syntax errors found.   CIF dictionary   Interpreting this report

Datablock: hasj240516a

Bond precision:	C-C = 0.0051 A		Wavelength=1.54184
Cell:	a=18.0228(3)	b=13.7648(2)	c=19.3726(4)
	alpha=90	beta=108.7068(19)	gamma=90
Temperature:	100 K		

	Calculated	Reported
Volume	4552.07(15)	4552.08(15)
Space group	P 21/n	P 1 21/n 1
Hall group	-P 2yn	-P 2yn
Moiety formula	C96 H144 N4 O2 Yb2, C5 H12	C96 H144 N4 O2 Yb2, C5 H12
Sum formula	C101 H156 N4 O2 Yb2	C101 H156 N4 O2 Yb2
Mr	1804.38	1804.37
Dx,g cm-3	1.316	1.316
Z	2	2
Mu (mm-1)	4.063	4.063
F000	1892.0	1892.0
F000'	1867.00	
h,k,lmax	22,17,23	22,16,23
Nref	9005	8806
Tmin,Tmax	0.674,0.964	0.562,1.000
Tmin'	0.419	

Correction method= # Reported T Limits: Tmin=0.562 Tmax=1.000
AbsCorr = MULTI-SCAN

Data completeness= 0.978   Theta(max)= 72.403

R(reflections)= 0.0349(7590)   wR2(reflections)= 0.0919(8806)

S = 1.037   Npar= 610

The following ALERTS were generated. Each ALERT has the format
test-name_ALERT_alert-type_alert-level.
Click on the hyperlinks for more details of the test.

Alert level C

```
PLAT220_ALERT_2_C NonSolvent   Resd 1  C   Ueq(max)/Ueq(min) Range        3.2 Ratio
PLAT911_ALERT_3_C Missing FCF Refl Between Thmin & STh/L=    0.600         20 Report
                0  8  0,   1  9  0,   2  9  0,  -4  8  1,  -1  9  1,   0  9  1,
                1  9  1,   2  9  1,   0  9  2,   1  9  2, -16 11  4,   1 16  5,
                5  9 16, -11 10 17, -10 10 17, -15  0 21,   0  0 22,  -9  0 23,
               -7  0 23,  -5  0 23,
PLAT971_ALERT_2_C Check Calcd Resid. Dens.  1.06Ang From Yb1            1.70 eA-3
PLAT971_ALERT_2_C Check Calcd Resid. Dens.  1.11Ang From Yb1            1.58 eA-3
```

Alert level G

```
PLAT002_ALERT_2_G Number of Distance or Angle Restraints on AtSite         19 Note
PLAT003_ALERT_2_G Number of Uiso or U(i,j)  Restrained non-H-Atoms         19 Report
PLAT164_ALERT_4_G Nr. of Refined C-H H-Atoms in Heavy-Atom Struct.          6 Note
PLAT176_ALERT_4_G The CIF-Embedded .res File Contains SADI Records          6 Report
PLAT178_ALERT_4_G The CIF-Embedded .res File Contains SIMU Records          3 Report
PLAT187_ALERT_4_G The CIF-Embedded .res File Contains RIGU Records          3 Report
PLAT191_ALERT_3_G A Non-default SADI Restraint Value has been used     0.0100 Report
PLAT191_ALERT_3_G A Non-default SADI Restraint Value has been used     0.0100 Report
PLAT299_ALERT_4_G Atom Site Occupancy Constrained at ............         0.5 Check
              C49     C50     C51     C52     C53     H49A    H49B    H49C
              H50A    H50B    H51A    H51B    H52A    H52B    H53A    H53B
              H53C
PLAT301_ALERT_3_G Main Residue  Disorder .............(Resd    1)         12% Note
PLAT302_ALERT_4_G Anion/Solvent/Minor-Residue Disorder (Resd    2)        100% Note
PLAT304_ALERT_4_G Non-Integer Number of Atoms in ..... (Resd    2)        8.50 Check
PLAT333_ALERT_2_G Large Aver C6-Ring C-C Dist C38      -C43      .        1.42 Ang.
PLAT333_ALERT_2_G Large Aver C6-Ring C-C Dist C38      -C44_a    .        1.43 Ang.
PLAT410_ALERT_2_G Short Intra H...H Contact  H10      ..H20C     .        2.14 Ang.
                                                     x,y,z  =      1_555 Check
PLAT722_ALERT_1_G Angle   Calc      108.00, Rep      106.90 Dev...        1.10 Degree
              C17     -C20A    -H20A       1_555  1_555  1_555     #  283 Check
PLAT779_ALERT_4_G Suspect or Irrelevant (Bond) Angle(s) in CIF ...       43.85 Deg.
              N2   -C4   -YB1     1_555   1_555   1_555 ......     #   83 Check
PLAT789_ALERT_4_G Atoms with Negative _atom_site_disorder_group  #         17 Check
PLAT822_ALERT_4_G CIF-embedded .res Contains Negative PART Numbers          1 Check
PLAT860_ALERT_3_G Number of Least-Squares Restraints ............         251 Note
PLAT910_ALERT_3_G Missing # of FCF Reflection(s) Below Theta(Min).          1 Note
               -1  0  1,
PLAT912_ALERT_4_G Missing # of FCF Reflections Above STh/L=  0.600        175 Note
PLAT941_ALERT_3_G Average HKL Measurement Multiplicity ..........         2.9 Low
PLAT969_ALERT_5_G The 'Henn et al.' R-Factor-gap value ..........       2.173 Note
              Predicted wR2: Based on SigI**2  4.23 or SHELX Weight  8.86
PLAT978_ALERT_2_G Number C-C Bonds with Positive Residual Density.          2 Info
```

---

0 **ALERT level A** = Most likely a serious problem - resolve or explain
0 **ALERT level B** = A potentially serious problem, consider carefully
4 **ALERT level C** = Check. Ensure it is not caused by an omission or oversight
25 **ALERT level G** = General information/check it is not something unexpected

```
 1 ALERT type 1 CIF construction/syntax error, inconsistent or missing data
 9 ALERT type 2 Indicator that the structure model may be wrong or deficient
 7 ALERT type 3 Indicator that the structure quality may be low
11 ALERT type 4 Improvement, methodology, query or suggestion
 1 ALERT type 5 Informative message, check
```

It is advisable to attempt to resolve as many as possible of the alerts in all categories. Often the minor alerts point to easily fixed oversights, errors and omissions in your CIF or refinement strategy, so attention to these fine details can be worthwhile. In order to resolve some of the more serious problems it may be necessary to carry out additional measurements or structure refinements. However, the purpose of your study may justify the reported deviations and the more serious of these should normally be commented upon in the discussion or experimental section of a paper or in the "special_details" fields of the CIF. checkCIF was carefully designed to identify outliers and unusual parameters, but every test has its limitations and alerts that are not important in a particular case may appear. Conversely, the absence of alerts does not guarantee there are no aspects of the results needing attention. It is up to the individual to critically assess their own results and, if necessary, seek expert advice.

Publication of your CIF in IUCr journals

A basic structural check has been run on your CIF. These basic checks will be run on all CIFs submitted for publication in IUCr journals (*Acta Crystallographica*, *Journal of Applied Crystallography*, *Journal of Synchrotron Radiation*); however, if you intend to submit to *Acta Crystallographica Section C* or *E* or *IUCrData*, you should make sure that full publication checks are run on the final version of your CIF prior to submission.

Publication of your CIF in other journals

Please refer to the *Notes for Authors* of the relevant journal for any special instructions relating to CIF submission.

PLATON version of 02/02/2025; check.def file version of 02/02/2025

Datablock hasj240516a - ellipsoid plot

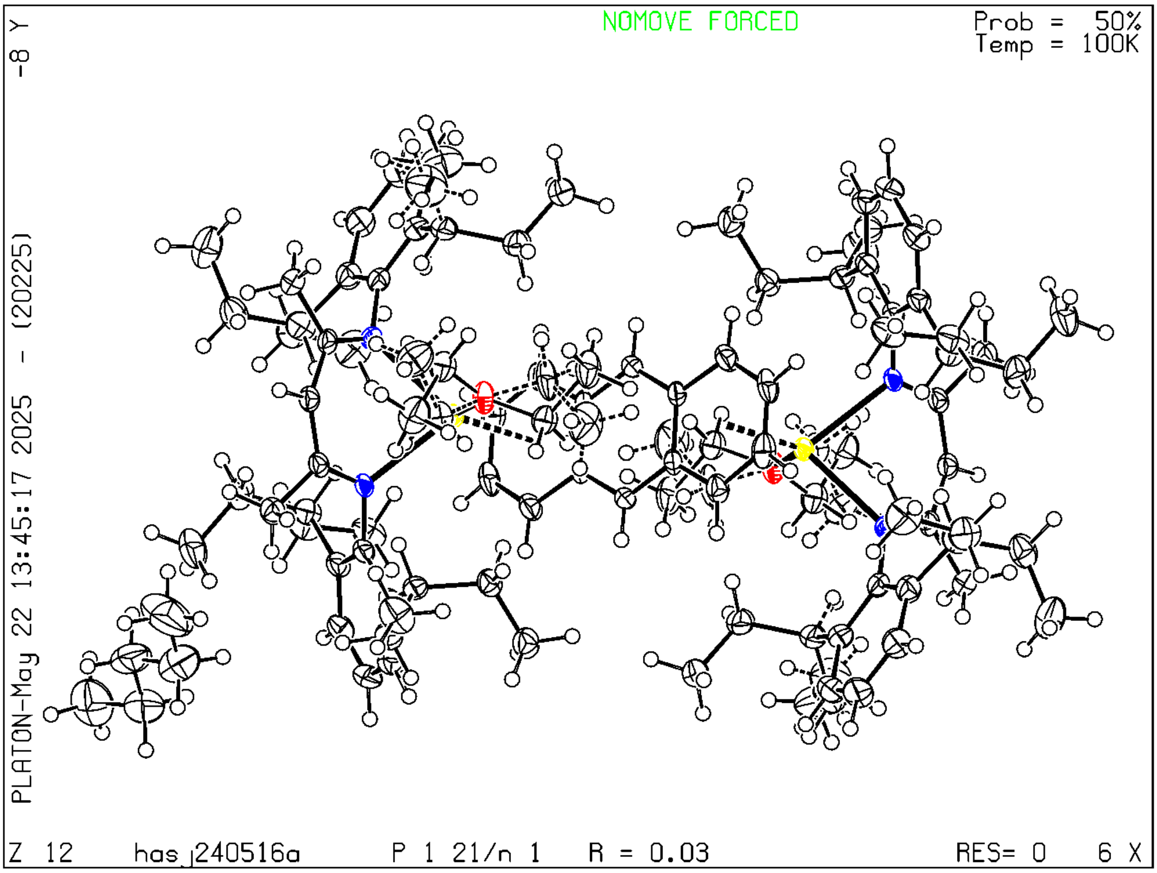

checkCIF/PLATON report

Structure factors have been supplied for datablock(s) hasj231023c

THIS REPORT IS FOR GUIDANCE ONLY. IF USED AS PART OF A REVIEW PROCEDURE FOR PUBLICATION, IT SHOULD NOT REPLACE THE EXPERTISE OF AN EXPERIENCED CRYSTALLOGRAPHIC REFEREE.

No syntax errors found. CIF dictionary Interpreting this report

Datablock: hasj231023c

Bond precision:	C-C = 0.0109 A		Wavelength=1.54184
Cell:	a=29.0514(4)	b=22.0120(2)	c=26.0740(4)
	alpha=90	beta=116.661(2)	gamma=90
Temperature:	100 K		

	Calculated	Reported
Volume	14901.0(4)	14901.0(4)
Space group	P 21/c	P 1 21/c 1
Hall group	-P 2ybc	-P 2ybc
Moiety formula	C82 H122 N4 Yb2	1(C82 H122 N4 Yb2)
Sum formula	C82 H122 N4 Yb2	C82 H122 N4 Yb2
Mr	1509.92	1509.91
Dx,g cm-3	1.346	1.346
Z	8	8
Mu (mm-1)	4.840	4.840
F000	6256.0	6256.0
F000'	6152.66	
h,k,lmax	35,27,32	35,26,32
Nref	29542	29002
Tmin,Tmax	0.276,0.546	0.430,1.000
Tmin'	0.176	

Correction method= # Reported T Limits: Tmin=0.430 Tmax=1.000
AbsCorr = GAUSSIAN

Data completeness= 0.982 Theta(max)= 72.516

R(reflections)= 0.0416(25057) wR2(reflections)= 0.1088(29002)

S = 1.025 Npar= 2436

The following ALERTS were generated. Each ALERT has the format
test-name_ALERT_alert-type_alert-level.
Click on the hyperlinks for more details of the test.

Alert level C

```
PLAT042_ALERT_1_C Calc. and Reported MoietyFormula Strings  Differ     Please Check
              Calc: C82 H122 N4 Yb2
              Rep.: 1(C82 H122 N4 Yb2)
PLAT220_ALERT_2_C NonSolvent   Resd 2  C   Ueq(max)/Ueq(min) Range        3.5 Ratio
PLAT222_ALERT_3_C NonSolvent Resd 2  H   Uiso(max)/Uiso(min) Range        4.3 Ratio
PLAT342_ALERT_3_C Low Bond Precision on  C-C Bonds ...............    0.01089 Ang.
PLAT911_ALERT_3_C Missing FCF Refl Between Thmin & STh/L=    0.600         33 Report
                2  0  0,  20 17  6,  19 17  7,  18 17  8,  25  1  9,  25  2  9,
              -21 19 10, -19 22 10,  23  0 10,  24  0 10,  24  1 10, -21 19 11,
              -20 20 11,  23  1 11, -21 19 12, -20 20 12, -19 20 12, -19 21 12,
              -18 22 12,  22  0 12, -20 20 13, -19 21 13, -18 21 13, -17 22 13,
              -19 21 14, -18 21 14, -17 22 14,  20  0 14, -19 21 15,  18  0 16,
              -34  0 18, -30  1 18, -30  0 20,
```

Alert level G

```
PLAT002_ALERT_2_G Number of Distance or Angle Restraints on AtSite        156 Note
PLAT003_ALERT_2_G Number of Uiso or U(i,j)  Restrained non-H-Atoms        177 Report
PLAT083_ALERT_2_G SHELXL Second Parameter in WGHT  Unusually Large      24.54 Why ?
PLAT142_ALERT_4_G s.u. on b - Axis Small or Missing .............    0.00020 Ang.
PLAT174_ALERT_4_G The CIF-Embedded .res File Contains FLAT Records          2 Report
PLAT176_ALERT_4_G The CIF-Embedded .res File Contains SADI Records         10 Report
PLAT178_ALERT_4_G The CIF-Embedded .res File Contains SIMU Records          7 Report
PLAT187_ALERT_4_G The CIF-Embedded .res File Contains RIGU Records          4 Report
PLAT188_ALERT_3_G A Non-default SIMU Restraint Value has been used     0.0200 Report
PLAT188_ALERT_3_G A Non-default SIMU Restraint Value has been used     0.0200 Report
PLAT188_ALERT_3_G A Non-default SIMU Restraint Value has been used     0.0200 Report
PLAT188_ALERT_3_G A Non-default SIMU Restraint Value has been used     0.0200 Report
PLAT188_ALERT_3_G A Non-default SIMU Restraint Value has been used     0.0200 Report
PLAT188_ALERT_3_G A Non-default SIMU Restraint Value has been used     0.0200 Report
PLAT188_ALERT_3_G A Non-default SIMU Restraint Value has been used     0.0200 Report
PLAT190_ALERT_3_G A Non-default RIGU Restraint Value for First Par     0.0020 Report
PLAT190_ALERT_3_G A Non-default RIGU Restraint Value for SecondPar     0.0020 Report
PLAT301_ALERT_3_G Main Residue  Disorder ..............(Resd    1)        52% Note
PLAT301_ALERT_3_G Main Residue  Disorder ..............(Resd    2)        47% Note
PLAT380_ALERT_4_G Incorrectly? Oriented X(sp2)-Methyl Moiety .....       C128 Check
PLAT410_ALERT_2_G Short Intra H...H Contact  H31B      ..H42       .       2.13 Ang.
                                                       x,y,z  =      1_555 Check
PLAT410_ALERT_2_G Short Intra H...H Contact  H31B      ..H74B      .       2.09 Ang.
                                                       x,y,z  =      1_555 Check
PLAT410_ALERT_2_G Short Intra H...H Contact  H71       ..H20H      .       1.83 Ang.
                                                       x,y,z  =      1_555 Check
PLAT412_ALERT_2_G Short Intra XH3 .. XHn     H11L      ..H87C      .       1.81 Ang.
                                                       x,y,z  =      1_555 Check
PLAT412_ALERT_2_G Short Intra XH3 .. XHn     H83A      ..H97A      .       2.00 Ang.
                                                       x,y,z  =      1_555 Check
PLAT412_ALERT_2_G Short Intra XH3 .. XHn     H83C      ..H21K      .       2.02 Ang.
                                                       x,y,z  =      1_555 Check
PLAT412_ALERT_2_G Short Intra XH3 .. XHn     H87A      ..H22B      .       1.98 Ang.
                                                       x,y,z  =      1_555 Check
```

```
PLAT774_ALERT_1_G Check   X-Y Bond in CIF: Yb3      --Yb4       ..       4.04 Ang.
PLAT779_ALERT_4_G Suspect or Irrelevant (Bond) Angle(s) in CIF ...      44.80 Deg.
              N2   -C22  -YB1     1_555   1_555   1_555 ......     #  482 Check
PLAT811_ALERT_5_G No ADDSYM Analysis: Too Many Excluded Atoms ....          ! Info
PLAT860_ALERT_3_G Number of Least-Squares Restraints ............      11748 Note
PLAT870_ALERT_4_G ALERTS Related to Twinning Effects Suppressed ..          ! Info
PLAT910_ALERT_3_G Missing # of FCF Reflection(s) Below Theta(Min).          4 Note
                1  0  0,   1  1  0,  -1  1  1,   0  1  1,
PLAT912_ALERT_4_G Missing # of FCF Reflections Above STh/L=  0.600        473 Note
PLAT931_ALERT_5_G CIFcalcFCF  Twin Law ( 1 0 0)         Est.d BASF       0.49 Check
PLAT933_ALERT_2_G Number of HKL-OMIT Records in Embedded .res File          3 Note
              -34  0 18, -30  0 20, -30  1 18,
PLAT941_ALERT_3_G Average HKL Measurement Multiplicity ..........        3.9 Low
PLAT969_ALERT_5_G The 'Henn et al.' R-Factor-gap value ..........      2.370 Note
              Predicted wR2: Based on SigI**2  4.59 or SHELX Weight 10.61
```

---

0 **ALERT level A** = Most likely a serious problem - resolve or explain
0 **ALERT level B** = A potentially serious problem, consider carefully
5 **ALERT level C** = Check. Ensure it is not caused by an omission or oversight
38 **ALERT level G** = General information/check it is not something unexpected

2 ALERT type 1 CIF construction/syntax error, inconsistent or missing data
12 ALERT type 2 Indicator that the structure model may be wrong or deficient
17 ALERT type 3 Indicator that the structure quality may be low
9 ALERT type 4 Improvement, methodology, query or suggestion
3 ALERT type 5 Informative message, check

It is advisable to attempt to resolve as many as possible of the alerts in all categories. Often the minor alerts point to easily fixed oversights, errors and omissions in your CIF or refinement strategy, so attention to these fine details can be worthwhile. In order to resolve some of the more serious problems it may be necessary to carry out additional measurements or structure refinements. However, the purpose of your study may justify the reported deviations and the more serious of these should normally be commented upon in the discussion or experimental section of a paper or in the "special_details" fields of the CIF. checkCIF was carefully designed to identify outliers and unusual parameters, but every test has its limitations and alerts that are not important in a particular case may appear. Conversely, the absence of alerts does not guarantee there are no aspects of the results needing attention. It is up to the individual to critically assess their own results and, if necessary, seek expert advice.

Publication of your CIF in IUCr journals

A basic structural check has been run on your CIF. These basic checks will be run on all CIFs submitted for publication in IUCr journals (*Acta Crystallographica*, *Journal of Applied Crystallography*, *Journal of Synchrotron Radiation*); however, if you intend to submit to *Acta Crystallographica Section C* or *E* or *IUCrData*, you should make sure that full publication checks are run on the final version of your CIF prior to submission.

Publication of your CIF in other journals

Please refer to the *Notes for Authors* of the relevant journal for any special instructions relating to CIF submission.

PLATON version of 02/02/2025; check.def file version of 02/02/2025

Datablock hasj231023c - ellipsoid plot

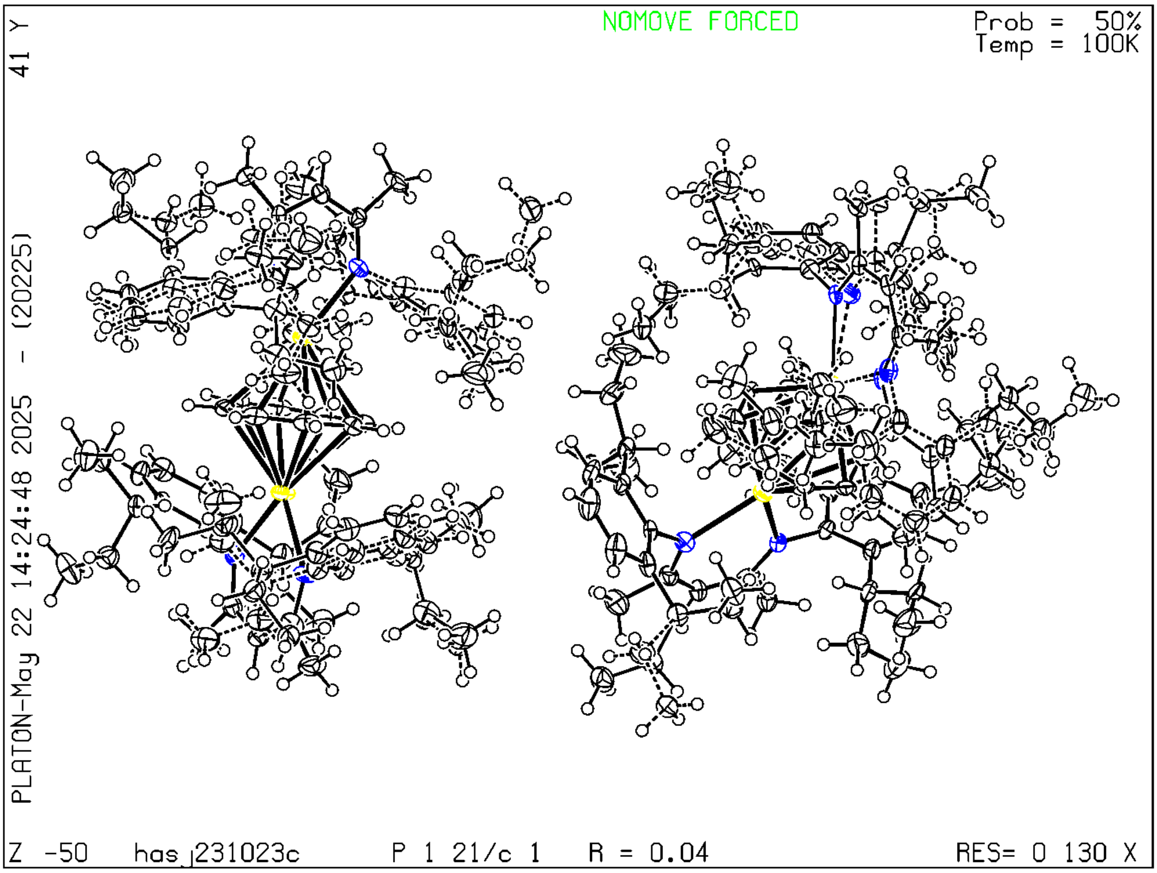

checkCIF/PLATON report

Structure factors have been supplied for datablock(s) hasj240119b

THIS REPORT IS FOR GUIDANCE ONLY. IF USED AS PART OF A REVIEW PROCEDURE FOR PUBLICATION, IT SHOULD NOT REPLACE THE EXPERTISE OF AN EXPERIENCED CRYSTALLOGRAPHIC REFEREE.

No syntax errors found. CIF dictionary Interpreting this report

Datablock: hasj240119b

Bond precision: C-C = 0.0054 A Wavelength=1.54184

Cell:	a=13.5490(2)	b=23.1836(3)	c=14.6662(2)
	alpha=90	beta=106.398(1)	gamma=90
Temperature:	100 K		

	Calculated	Reported
Volume	4419.48(11)	4419.48(11)
Space group	P 21/n	P 1 21/n 1
Hall group	-P 2yn	-P 2yn
Moiety formula	C49 H73 N2 O Sm	C49 H73 N2 O Sm
Sum formula	C49 H73 N2 O Sm	C49 H73 N2 O Sm
Mr	856.45	856.44
Dx,g cm-3	1.287	1.287
Z	4	4
Mu (mm-1)	10.237	10.237
F000	1804.0	1804.0
F000'	1787.11	
h,k,lmax	16,28,18	16,27,18
Nref	8781	8650
Tmin,Tmax	0.137,0.743	0.139,1.000
Tmin'	0.024	

Correction method= # Reported T Limits: Tmin=0.139 Tmax=1.000
AbsCorr = GAUSSIAN

Data completeness= 0.985 Theta(max)= 72.639

R(reflections)= 0.0318(7593) wR2(reflections)= 0.0812(8650)

S = 1.017 Npar= 779

The following ALERTS were generated. Each ALERT has the format
test-name_ALERT_alert-type_alert-level.
Click on the hyperlinks for more details of the test.

Alert level C

```
PLAT911_ALERT_3_C Missing FCF Refl Between Thmin & STh/L=    0.600          6 Report
                2 27  1,   3 27  1,  14  0  4,  14  1  4,  13  1  6,  11  0  9,
```

Alert level G

```
PLAT002_ALERT_2_G Number of Distance or Angle Restraints on AtSite          66 Note
PLAT003_ALERT_2_G Number of Uiso or U(i,j)  Restrained non-H-Atoms          54 Report
PLAT176_ALERT_4_G The CIF-Embedded .res File Contains SADI Records          12 Report
PLAT178_ALERT_4_G The CIF-Embedded .res File Contains SIMU Records           3 Report
PLAT187_ALERT_4_G The CIF-Embedded .res File Contains RIGU Records           2 Report
PLAT188_ALERT_3_G A Non-default SIMU Restraint Value has been used      0.0300 Report
PLAT188_ALERT_3_G A Non-default SIMU Restraint Value has been used      0.0300 Report
PLAT188_ALERT_3_G A Non-default SIMU Restraint Value has been used      0.0200 Report
PLAT230_ALERT_2_G Hirshfeld Test Diff for    C11      --C17A     .         7.7 s.u.
PLAT301_ALERT_3_G Main Residue  Disorder .............(Resd    1)          58% Note
PLAT410_ALERT_2_G Short Intra H...H Contact  H10      ..H18C     .        2.13 Ang.
                                                   x,y,z  =      1_555 Check
PLAT412_ALERT_2_G Short Intra XH3 .. XHn     H19B     ..H42      .        2.03 Ang.
                                                   x,y,z  =      1_555 Check
PLAT413_ALERT_2_G Short Inter XH3 .. XHn     H5A      ..H19D     .        2.05 Ang.
                                      -1/2+x,3/2-y,1/2+z  =      4_576 Check
PLAT811_ALERT_5_G No ADDSYM Analysis: Too Many Excluded Atoms ....           ! Info
PLAT860_ALERT_3_G Number of Least-Squares Restraints ............         1967 Note
PLAT912_ALERT_4_G Missing # of FCF Reflections Above STh/L=  0.600         103 Note
PLAT941_ALERT_3_G Average HKL Measurement Multiplicity ..........          4.8 Low
PLAT969_ALERT_5_G The 'Henn et al.' R-Factor-gap value ..........        2.653 Note
              Predicted wR2: Based on SigI**2  3.06 or SHELX Weight  7.99
PLAT978_ALERT_2_G Number C-C Bonds with Positive Residual Density.           5 Info
```

0 **ALERT level A** = Most likely a serious problem - resolve or explain
0 **ALERT level B** = A potentially serious problem, consider carefully
1 **ALERT level C** = Check. Ensure it is not caused by an omission or oversight
19 **ALERT level G** = General information/check it is not something unexpected

0 ALERT type 1 CIF construction/syntax error, inconsistent or missing data
7 ALERT type 2 Indicator that the structure model may be wrong or deficient
7 ALERT type 3 Indicator that the structure quality may be low
4 ALERT type 4 Improvement, methodology, query or suggestion
2 ALERT type 5 Informative message, check

It is advisable to attempt to resolve as many as possible of the alerts in all categories. Often the minor alerts point to easily fixed oversights, errors and omissions in your CIF or refinement strategy, so attention to these fine details can be worthwhile. In order to resolve some of the more serious problems it may be necessary to carry out additional measurements or structure refinements. However, the purpose of your study may justify the reported deviations and the more serious of these should normally be commented upon in the discussion or experimental section of a paper or in the "special_details" fields of the CIF. checkCIF was carefully designed to identify outliers and unusual parameters, but every test has its limitations and alerts that are not important in a particular case may appear. Conversely, the absence of alerts does not guarantee there are no aspects of the results needing attention. It is up to the individual to critically assess their own results and, if necessary, seek expert advice.

Publication of your CIF in IUCr journals

A basic structural check has been run on your CIF. These basic checks will be run on all CIFs submitted for publication in IUCr journals (*Acta Crystallographica*, *Journal of Applied Crystallography*, *Journal of Synchrotron Radiation*); however, if you intend to submit to *Acta Crystallographica Section C* or *E* or *IUCrData*, you should make sure that full publication checks are run on the final version of your CIF prior to submission.

Publication of your CIF in other journals

Please refer to the *Notes for Authors* of the relevant journal for any special instructions relating to CIF submission.

PLATON version of 02/02/2025; check.def file version of 02/02/2025

Datablock hasj240119b - ellipsoid plot

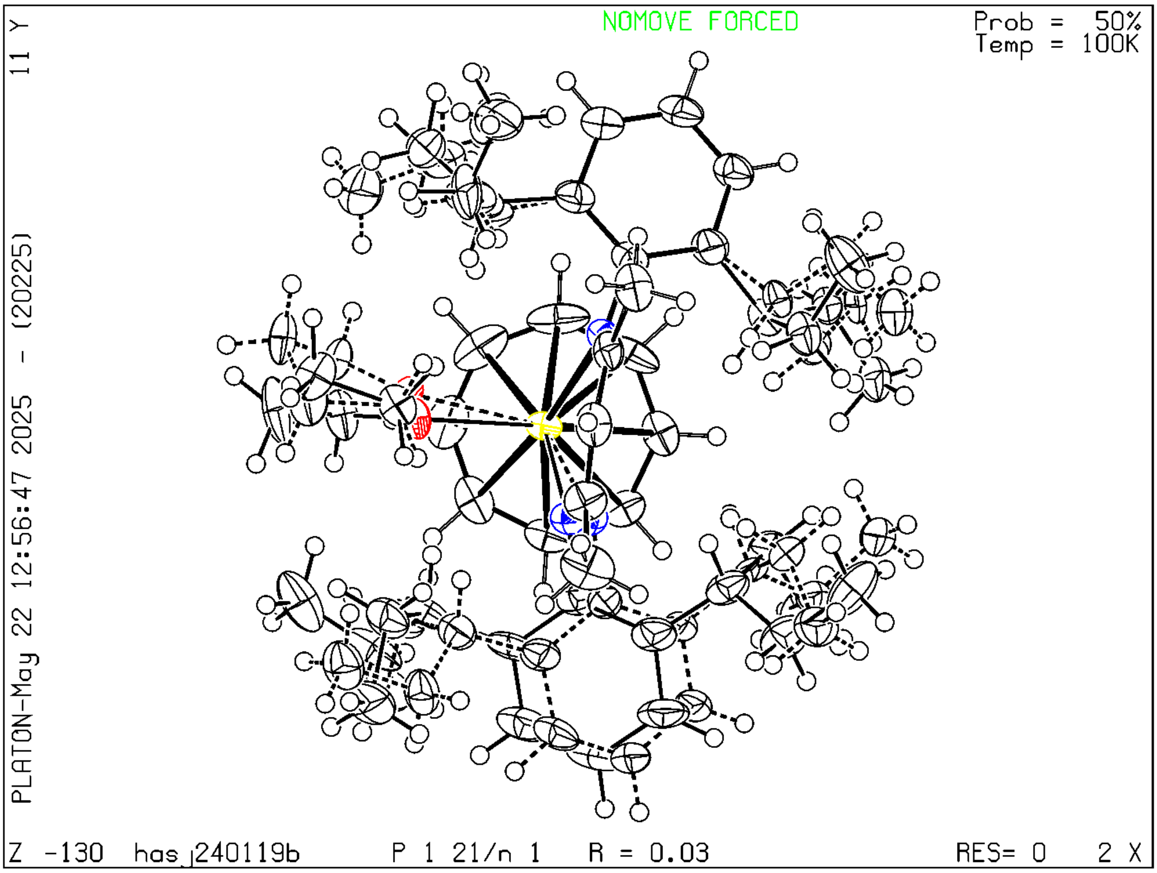

checkCIF/PLATON report

Structure factors have been supplied for datablock(s) hasj250801a

THIS REPORT IS FOR GUIDANCE ONLY. IF USED AS PART OF A REVIEW PROCEDURE FOR PUBLICATION, IT SHOULD NOT REPLACE THE EXPERTISE OF AN EXPERIENCED CRYSTALLOGRAPHIC REFEREE.

No syntax errors found.   CIF dictionary   Interpreting this report

Datablock: hasj250801a

Bond precision: C-C = 0.0106 A   Wavelength=1.54184

Cell:	a=18.1522(11)	b=13.9234(8)	c=19.2025(13)
	alpha=90	beta=108.012(7)	gamma=90
Temperature:	100 K		

	Calculated	Reported
Volume	4615.4(5)	4615.4(5)
Space group	P 21/n	P 1 21/n 1
Hall group	-P 2yn	-P 2yn
Moiety formula	C98 H144 N4 O2 Sm2 [+ solvent]	C98 H144 N4 O2 Sm2, 1[C6H12]
Sum formula	C98 H144 N4 O2 Sm2 [+ solvent]	C104 H156 N4 O2 Sm2
Mr	1710.89	1795.02
Dx,g cm-3	1.231	1.292
Z	2	2
Mu (mm-1)	9.802	9.826
F000	1800.0	1896.0
F000’	1783.11	
h,k,lmax	22,17,23	22,16,23
Nref	9046	8760
Tmin,Tmax	0.121,0.855	0.204,0.852
Tmin’	0.054	

Correction method= # Reported T Limits: Tmin=0.204 Tmax=0.852
AbsCorr = ANALYTICAL

Data completeness= 0.968   Theta(max)= 71.842

R(reflections)= 0.0597(6653)   wR2(reflections)= 0.1725(8760)

S = 1.046   Npar= 574

The following ALERTS were generated. Each ALERT has the format
test-name_ALERT_alert-type_alert-level.
Click on the hyperlinks for more details of the test.

Alert level C

```
PLAT220_ALERT_2_C NonSolvent   Resd 1  C   Ueq(max)/Ueq(min) Range        3.1 Ratio
PLAT222_ALERT_3_C NonSolvent Resd 1  H   Uiso(max)/Uiso(min) Range        4.5 Ratio
PLAT342_ALERT_3_C Low Bond Precision on  C-C Bonds ...............    0.01057 Ang.
PLAT911_ALERT_3_C Missing FCF Refl Between Thmin & STh/L=    0.600         24 Report
              -18  9  5, -18  9  6, -18  9  7, -18  9  8, -12  9  8, -18  9  9,
               13  5 12,  -9 13 13,  12  5 13,  -7 13 14,  11  5 14,   9  6 15,
               10  5 15,   8  6 16,   6  6 17,   5  6 18,   6  5 18, -15  2 19,
                3  6 19,   4  5 19,   1  6 20,   2  5 20,  -2  6 21,  -1  5 21,
PLAT972_ALERT_2_C Check Calcd Resid. Dens.  1.00Ang From Sm1           -2.06 eA-3
PLAT972_ALERT_2_C Check Calcd Resid. Dens.  0.78Ang From Sm1           -1.81 eA-3
PLAT972_ALERT_2_C Check Calcd Resid. Dens.  0.79Ang From Sm1           -1.58 eA-3
```

Alert level G

```
FORMU01_ALERT_2_G  There is a discrepancy between the atom counts in the
            _chemical_formula_sum and the formula from the _atom_site* data.
            Atom count from _chemical_formula_sum:C104 H156 N4 O2 Sm2
            Atom count from the _atom_site data:  C98 H144 N4 O2 Sm2
CELLZ01_ALERT_1_G Difference between formula and atom_site contents detected.
CELLZ01_ALERT_1_G ALERT: Large difference may be due to a
            symmetry error - see SYMMG tests
           From the CIF: _cell_formula_units_Z    2
           From the CIF: _chemical_formula_sum  C104 H156 N4 O2 Sm2
           TEST: Compare cell contents of formula and atom_site data
```

atom	Z*formula	cif sites	diff
C	208.00	196.00	12.00
H	312.00	288.00	24.00
N	8.00	8.00	0.00
O	4.00	4.00	0.00
Sm	4.00	4.00	0.00

```
PLAT002_ALERT_2_G Number of Distance or Angle Restraints on AtSite         18 Note
PLAT003_ALERT_2_G Number of Uiso or U(i,j)  Restrained non-H-Atoms         16 Report
PLAT041_ALERT_1_G Calc. and Reported SumFormula    Strings  Differ     Please Check
              Calc: C98 H144 N4 O2 Sm2
              Rep.: C104 H156 N4 O2 Sm2
PLAT042_ALERT_1_G Calc. and Reported MoietyFormula Strings  Differ     Please Check
              Calc: C98 H144 N4 O2 Sm2
              Rep.: C98 H144 N4 O2 Sm2, 1[C6H12]
PLAT164_ALERT_4_G Nr. of Refined C-H H-Atoms in Heavy-Atom Struct.          5 Note
PLAT176_ALERT_4_G The CIF-Embedded .res File Contains SADI Records         12 Report
PLAT178_ALERT_4_G The CIF-Embedded .res File Contains SIMU Records          2 Report
PLAT187_ALERT_4_G The CIF-Embedded .res File Contains RIGU Records          1 Report
PLAT188_ALERT_3_G A Non-default SIMU Restraint Value has been used     0.0200 Report
PLAT188_ALERT_3_G A Non-default SIMU Restraint Value has been used     0.0300 Report
```

```
PLAT301_ALERT_3_G Main Residue  Disorder ..............(Resd    1)        13% Note
PLAT333_ALERT_2_G Large Aver C6-Ring C-C Dist C39      -C41_a    .       1.43 Ang.
PLAT343_ALERT_2_G Unusual          Angle Range in Main Residue for        C44 Check
PLAT410_ALERT_2_G Short Intra H...H Contact  H8       ..H15B     .       1.80 Ang.
                                                     x,y,z  =     1_555 Check
PLAT412_ALERT_2_G Short Intra XH3 .. XHn     H1A      ..H16A     .       2.09 Ang.
                                                     x,y,z  =     1_555 Check
PLAT412_ALERT_2_G Short Intra XH3 .. XHn     H14C     ..H15A     .       1.88 Ang.
                                                     x,y,z  =     1_555 Check
PLAT413_ALERT_2_G Short Inter XH3 .. XHn     H30A     ..H49F     .       1.76 Ang.
                                    -1/2+x,1/2-y,-1/2+z  =     4_565 Check
PLAT605_ALERT_4_G Largest Solvent Accessible VOID in the Structure        146 A**3
PLAT722_ALERT_1_G Angle   Calc      113.00, Rep      111.60 Dev...       1.40 Degree
              C48A    -C47A    -H47D       1_555  1_555  1_555     #  317 Check
PLAT793_ALERT_4_G Model has Chirality at C45         (Centro SpGr)          S Verify
PLAT860_ALERT_3_G Number of Least-Squares Restraints .............        425 Note
PLAT868_ALERT_4_G ALERTS Due to the Use of _smtbx_masks Suppressed          ! Info
PLAT910_ALERT_3_G Missing FCF Reflection(s) Below Theta(Min)[Deg]=       3.99 Note
               -1  0  1,
PLAT912_ALERT_4_G Missing # of FCF Reflections Above STh/L=  0.600        247 Note
PLAT941_ALERT_3_G Average HKL Measurement Multiplicity ...........        2.1 Low
PLAT969_ALERT_5_G The 'Henn et al.' R-Factor-gap value ...........      2.229 Note
              Predicted wR2: Based on SigI**2  7.74 or SHELX Weight 16.50
PLAT978_ALERT_2_G Number C-C Bonds with Positive Residual Density.          1 Info
```

---

0 **ALERT level A** = Most likely a serious problem - resolve or explain
0 **ALERT level B** = A potentially serious problem, consider carefully
7 **ALERT level C** = Check. Ensure it is not caused by an omission or oversight
30 **ALERT level G** = General information/check it is not something unexpected

5 ALERT type 1 CIF construction/syntax error, inconsistent or missing data
14 ALERT type 2 Indicator that the structure model may be wrong or deficient
9 ALERT type 3 Indicator that the structure quality may be low
8 ALERT type 4 Improvement, methodology, query or suggestion
1 ALERT type 5 Informative message, check

It is advisable to attempt to resolve as many as possible of the alerts in all categories. Often the minor alerts point to easily fixed oversights, errors and omissions in your CIF or refinement strategy, so attention to these fine details can be worthwhile. In order to resolve some of the more serious problems it may be necessary to carry out additional measurements or structure refinements. However, the purpose of your study may justify the reported deviations and the more serious of these should normally be commented upon in the discussion or experimental section of a paper or in the "special_details" fields of the CIF. checkCIF was carefully designed to identify outliers and unusual parameters, but every test has its limitations and alerts that are not important in a particular case may appear. Conversely, the absence of alerts does not guarantee there are no aspects of the results needing attention. It is up to the individual to critically assess their own results and, if necessary, seek expert advice.

Publication of your CIF in IUCr journals

A basic structural check has been run on your CIF. These basic checks will be run on all CIFs submitted for publication in IUCr journals (*Acta Crystallographica*, *Journal of Applied Crystallography*, *Journal of Synchrotron Radiation*); however, if you intend to submit to *Acta Crystallographica Section C* or *E* or *IUCrData*, you should make sure that full publication checks are run on the final version of your CIF prior to submission.

Publication of your CIF in other journals

Please refer to the *Notes for Authors* of the relevant journal for any special instructions relating to CIF submission.

PLATON version of 04/06/2025; check.def file version of 30/05/2025

Datablock hasj250801a - ellipsoid plot

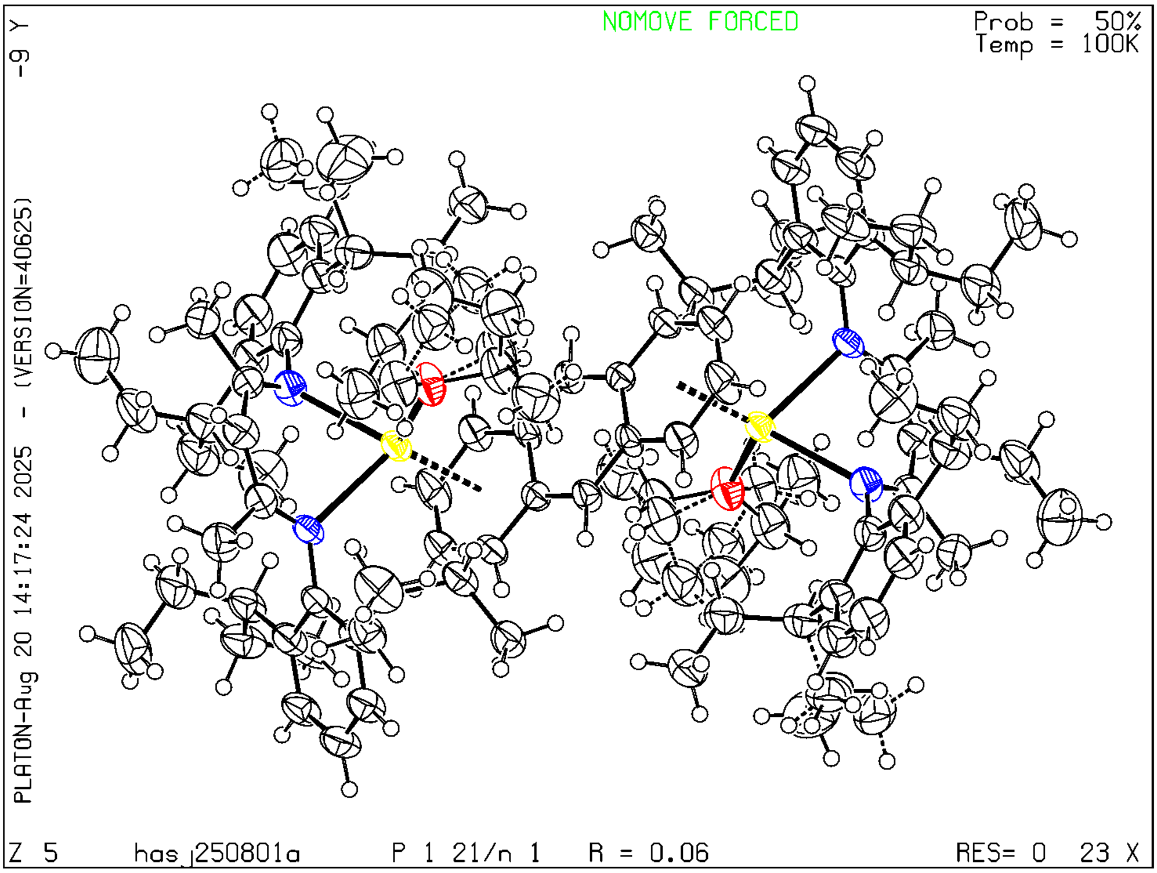

checkCIF/PLATON report

Structure factors have been supplied for datablock(s) hasj240205b

THIS REPORT IS FOR GUIDANCE ONLY. IF USED AS PART OF A REVIEW PROCEDURE FOR PUBLICATION, IT SHOULD NOT REPLACE THE EXPERTISE OF AN EXPERIENCED CRYSTALLOGRAPHIC REFEREE.

No syntax errors found. CIF dictionary Interpreting this report

Datablock: hasj240205b

Bond precision: C-C = 0.0058 A Wavelength=0.71073

Cell:	a=14.2111(3)	b=17.8935(4)	c=19.1462(4)
	alpha=80.0854(18)	beta=89.4096(16)	gamma=70.8174(19)
Temperature:	100 K		

	Calculated	Reported
Volume	4524.01(18)	4524.00(17)
Space group	P -1	P -1
Hall group	-P 1	-P 1
Moiety formula	C90 H132 N4 O Sm2, 0.186(C4 H8 O), 1.814(C5 H12)	C90 H132 N4 O Sm2, 0.186(C4 H8 O), 1.814(C5 H12)
Sum formula	C99.81 H155.26 N4 O1.19 Sm2	C99.82 H155.26 N4 O1.18 Sm2
Mr	1731.01	1730.97
Dx,g cm-3	1.271	1.271
Z	2	2
Mu (mm-1)	1.333	1.333
F000	1831.3	1831.0
F000'	1831.07	
h,k,lmax	18,23,25	18,22,25
Nref	22432	20526
Tmin,Tmax	0.775,0.963	0.572,1.000
Tmin'	0.723	

Correction method= # Reported T Limits: Tmin=0.572 Tmax=1.000
AbsCorr = GAUSSIAN

Data completeness= 0.915 Theta(max)= 28.281

R(reflections)= 0.0424(15303) wR2(reflections)= 0.0902(20526)

S = 1.044 Npar= 1102

The following ALERTS were generated. Each ALERT has the format
test-name_ALERT_alert-type_alert-level.
Click on the hyperlinks for more details of the test.

[IMAGE] **Alert level C**

```
PLAT041_ALERT_1_C Calc. and Reported SumFormula    Strings  Differ     Please Check
              Calc: C99.81 H155.26 N4 O1.19 Sm2
              Rep.: C99.82 H155.26 N4 O1.18 Sm2
PLAT077_ALERT_4_C Unit Cell Contains Non-integer Number of Atoms .     Please Check
PLAT220_ALERT_2_C NonSolvent   Resd 1  C   Ueq(max)/Ueq(min) Range        4.2 Ratio
PLAT222_ALERT_3_C NonSolvent Resd 1  H   Uiso(max)/Uiso(min) Range       10.0 Ratio
PLAT245_ALERT_2_C U(iso) H77       Smaller than U(eq) C77       by      0.014 Ang**2
PLAT350_ALERT_3_C Short  C-H (X0.96,N1.08A)  C77      - H77      .       0.83 Ang.
PLAT910_ALERT_3_C Missing # of FCF Reflection(s) Below Theta(Min).         10 Note
                1  0  0,   0  1  0,   1  1  0,  -1 -1  1,   0 -1  1,  -1  0  1,
                0  0  1,   1  0  1,   0  1  1,   1  1  1,
PLAT911_ALERT_3_C Missing FCF Refl Between Thmin & STh/L=    0.600          7 Report
                1  2  0,  -2  1  1,  -1  1  2,  -4  3  3,  -2  4  3,  -2  3  4,
               -1  9 16,
```

[IMAGE] **Alert level G**

```
PLAT002_ALERT_2_G Number of Distance or Angle Restraints on AtSite         20 Note
PLAT003_ALERT_2_G Number of Uiso or U(i,j)  Restrained non-H-Atoms         20 Report
PLAT083_ALERT_2_G SHELXL Second Parameter in WGHT  Unusually Large       5.76 Why ?
PLAT164_ALERT_4_G Nr. of Refined C-H H-Atoms in Heavy-Atom Struct.          5 Note
PLAT172_ALERT_4_G The CIF-Embedded .res File Contains DFIX Records          3 Report
PLAT173_ALERT_4_G The CIF-Embedded .res File Contains DANG Records          1 Report
PLAT176_ALERT_4_G The CIF-Embedded .res File Contains SADI Records          7 Report
PLAT178_ALERT_4_G The CIF-Embedded .res File Contains SIMU Records          2 Report
PLAT188_ALERT_3_G A Non-default SIMU Restraint Value has been used     0.0200 Report
PLAT188_ALERT_3_G A Non-default SIMU Restraint Value has been used     0.0200 Report
PLAT191_ALERT_3_G A Non-default SADI Restraint Value has been used     0.0400 Report
PLAT191_ALERT_3_G A Non-default SADI Restraint Value has been used     0.0400 Report
PLAT302_ALERT_4_G Anion/Solvent/Minor-Residue Disorder (Resd    2)       100% Note
PLAT302_ALERT_4_G Anion/Solvent/Minor-Residue Disorder (Resd    3)       100% Note
PLAT302_ALERT_4_G Anion/Solvent/Minor-Residue Disorder (Resd    4)       100% Note
PLAT302_ALERT_4_G Anion/Solvent/Minor-Residue Disorder (Resd    5)       100% Note
PLAT304_ALERT_4_G Non-Integer Number of Atoms in ..... (Resd    2)       2.42 Check
PLAT304_ALERT_4_G Non-Integer Number of Atoms in ..... (Resd    3)      12.90 Check
PLAT304_ALERT_4_G Non-Integer Number of Atoms in ..... (Resd    4)      13.84 Check
PLAT304_ALERT_4_G Non-Integer Number of Atoms in ..... (Resd    5)       4.10 Check
PLAT380_ALERT_4_G Incorrectly? Oriented X(sp2)-Methyl Moiety .....        C38 Check
PLAT398_ALERT_2_G Deviating  C-O-C   Angle From 120 for O2       .       96.5 Degree
PLAT722_ALERT_1_G Angle   Calc      110.00, Rep      108.90 Dev...       1.10 Degree
              C94A    -C93A    -H93B       1_555  1_555  1_555     #  608 Check
PLAT860_ALERT_3_G Number of Least-Squares Restraints ............         574 Note
PLAT912_ALERT_4_G Missing # of FCF Reflections Above STh/L=  0.600       1791 Note
PLAT933_ALERT_2_G Number of HKL-OMIT Records in Embedded .res File          2 Note
               -4  3  3,  -2  1  1,
PLAT941_ALERT_3_G Average HKL Measurement Multiplicity ..........         2.0 Low
PLAT969_ALERT_5_G The 'Henn et al.' R-Factor-gap value ..........       2.141 Note
              Predicted wR2: Based on SigI**2  4.21 or SHELX Weight  8.64
PLAT978_ALERT_2_G Number C-C Bonds with Positive Residual Density.          0 Info
```

```
 0 ALERT level A = Most likely a serious problem - resolve or explain
 0 ALERT level B = A potentially serious problem, consider carefully
 8 ALERT level C = Check. Ensure it is not caused by an omission or oversight
29 ALERT level G = General information/check it is not something unexpected

 2 ALERT type 1 CIF construction/syntax error, inconsistent or missing data
 8 ALERT type 2 Indicator that the structure model may be wrong or deficient
10 ALERT type 3 Indicator that the structure quality may be low
16 ALERT type 4 Improvement, methodology, query or suggestion
 1 ALERT type 5 Informative message, check
```

It is advisable to attempt to resolve as many as possible of the alerts in all categories. Often the minor alerts point to easily fixed oversights, errors and omissions in your CIF or refinement strategy, so attention to these fine details can be worthwhile. In order to resolve some of the more serious problems it may be necessary to carry out additional measurements or structure refinements. However, the purpose of your study may justify the reported deviations and the more serious of these should normally be commented upon in the discussion or experimental section of a paper or in the "special_details" fields of the CIF. checkCIF was carefully designed to identify outliers and unusual parameters, but every test has its limitations and alerts that are not important in a particular case may appear. Conversely, the absence of alerts does not guarantee there are no aspects of the results needing attention. It is up to the individual to critically assess their own results and, if necessary, seek expert advice.

Publication of your CIF in IUCr journals

A basic structural check has been run on your CIF. These basic checks will be run on all CIFs submitted for publication in IUCr journals (*Acta Crystallographica*, *Journal of Applied Crystallography*, *Journal of Synchrotron Radiation*); however, if you intend to submit to *Acta Crystallographica Section C* or *E* or *IUCrData*, you should make sure that full publication checks are run on the final version of your CIF prior to submission.

Publication of your CIF in other journals

Please refer to the *Notes for Authors* of the relevant journal for any special instructions relating to CIF submission.

PLATON version of 02/02/2025; check.def file version of 02/02/2025

Datablock hasj240205b - ellipsoid plot

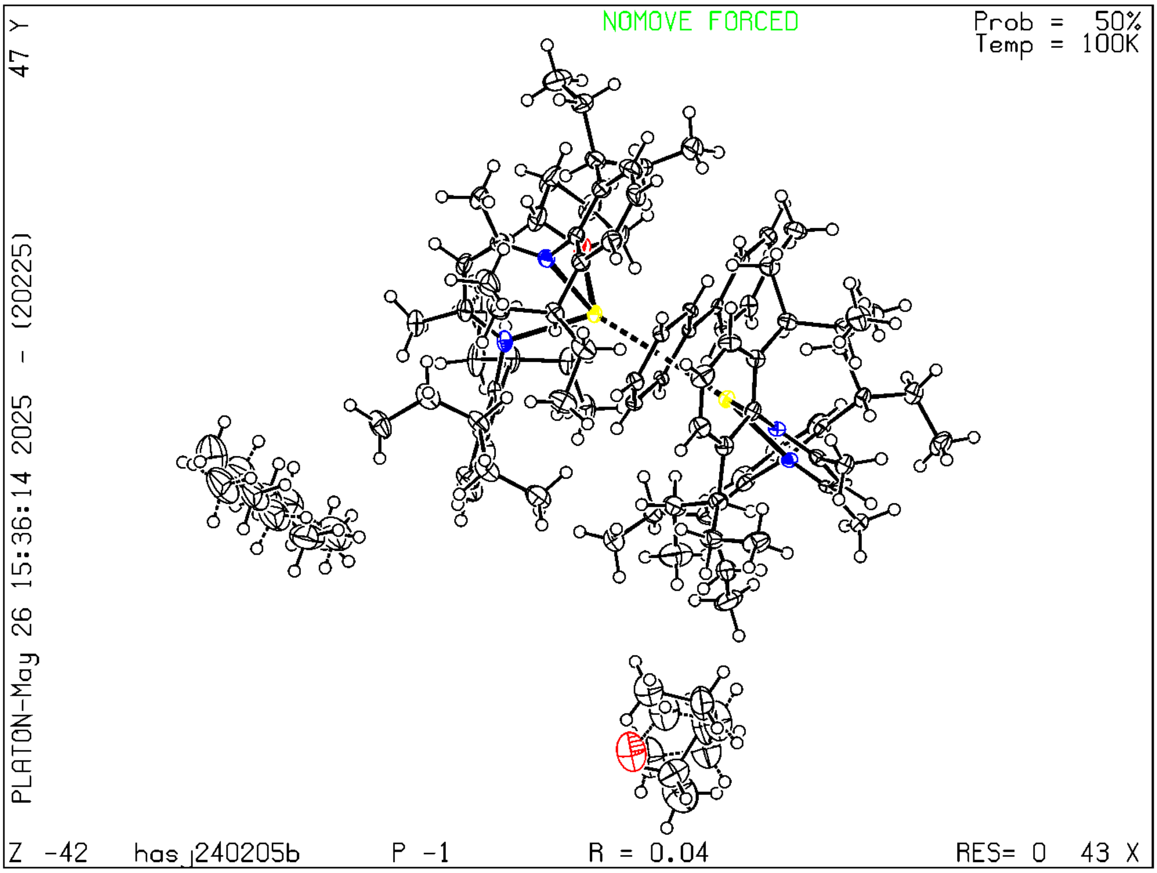

checkCIF/PLATON report

Structure factors have been supplied for datablock(s) hasj231122a

THIS REPORT IS FOR GUIDANCE ONLY. IF USED AS PART OF A REVIEW PROCEDURE FOR PUBLICATION, IT SHOULD NOT REPLACE THE EXPERTISE OF AN EXPERIENCED CRYSTALLOGRAPHIC REFEREE.

No syntax errors found. CIF dictionary Interpreting this report

Datablock: hasj231122a

Bond precision: C-C = 0.0040 A Wavelength=0.71073

Cell:	a=23.2368(4)	b=21.0517(4)	c=20.8273(4)
	alpha=90	beta=106.711(2)	gamma=90
Temperature:	100 K		

	Calculated	Reported
Volume	9757.9(3)	9757.9(3)
Space group	P 21/c	P 1 21/c 1
Hall group	-P 2ybc	-P 2ybc
Moiety formula	C98 H132 N4 Sm2, C6 H14 [+ solvent]	C98 H132 N4 Sm2, C6 H14, 1[C6H14]
Sum formula	C104 H146 N4 Sm2 [+ solvent]	C110 H160 N4 Sm2
Mr	1752.97	1839.11
Dx,g cm-3	1.193	1.252
Z	4	4
Mu (mm-1)	1.237	1.240
F000	3688.0	3888.0
F000'	3687.62	
h,k,lmax	32,29,28	31,28,28
Nref	27130	24641
Tmin,Tmax	0.686,0.881	0.581,1.000
Tmin'	0.649	

Correction method= # Reported T Limits: Tmin=0.581 Tmax=1.000
AbsCorr = GAUSSIAN

Data completeness= 0.908 Theta(max)= 29.482

R(reflections)= 0.0334(20463)

wR2(reflections)= 0.0718(24641)

S = 1.075 Npar= 1182

The following ALERTS were generated. Each ALERT has the format
test-name_ALERT_alert-type_alert-level.
Click on the hyperlinks for more details of the test.

[IMAGE] **Alert level C**

```
PLAT220_ALERT_2_C NonSolvent   Resd 1  C   Ueq(max)/Ueq(min) Range        4.1 Ratio
PLAT222_ALERT_3_C NonSolvent Resd 1  H   Uiso(max)/Uiso(min) Range        7.8 Ratio
PLAT250_ALERT_2_C Large U3/U1 Ratio for <U(i,j)> Tensor(Resd    2)        2.2 Note
PLAT910_ALERT_3_C Missing # of FCF Reflection(s) Below Theta(Min).          6 Note
               1  0  0,   1  1  0,   2  0  0,  -1  1  1,   0  1  1,   1  1  1,
PLAT911_ALERT_3_C Missing FCF Refl Between Thmin & STh/L=    0.600          3 Report
              -2  1  1,  -1  0  2,   1  0  2,
PLAT977_ALERT_2_C Check Negative Difference Density on H10A      .      -0.39 eA-3
```

[IMAGE] **Alert level G**

```
FORMU01_ALERT_2_G  There is a discrepancy between the atom counts in the
            _chemical_formula_sum and the formula from the _atom_site* data.
            Atom count from _chemical_formula_sum:C110 H160 N4 Sm2
            Atom count from the _atom_site data:  C104 H146 N4 Sm2
CELLZ01_ALERT_1_G Difference between formula and atom_site contents detected.
CELLZ01_ALERT_1_G ALERT: Large difference may be due to a
            symmetry error - see SYMMG tests
           From the CIF: _cell_formula_units_Z    4
           From the CIF: _chemical_formula_sum  C110 H160 N4 Sm2
           TEST: Compare cell contents of formula and atom_site data

           atom    Z*formula  cif sites diff
           C        440.00    416.00   24.00
           H        640.00    584.00   56.00
           N         16.00     16.00    0.00
           Sm         8.00      8.00    0.00
PLAT002_ALERT_2_G Number of Distance or Angle Restraints on AtSite          3 Note
PLAT003_ALERT_2_G Number of Uiso or U(i,j)  Restrained non-H-Atoms         23 Report
PLAT041_ALERT_1_G Calc. and Reported SumFormula    Strings  Differ     Please Check
             Calc: C104 H146 N4 Sm2
             Rep.: C110 H160 N4 Sm2
PLAT042_ALERT_1_G Calc. and Reported MoietyFormula Strings  Differ     Please Check
             Calc: C98 H132 N4 Sm2, C6 H14
             Rep.: C98 H132 N4 Sm2, C6 H14, 1[C6H14]
PLAT083_ALERT_2_G SHELXL Second Parameter in WGHT  Unusually Large      14.93 Why ?
PLAT164_ALERT_4_G Nr. of Refined C-H H-Atoms in Heavy-Atom Struct.          5 Note
PLAT176_ALERT_4_G The CIF-Embedded .res File Contains SADI Records          1 Report
PLAT178_ALERT_4_G The CIF-Embedded .res File Contains SIMU Records          1 Report
PLAT187_ALERT_4_G The CIF-Embedded .res File Contains RIGU Records          2 Report
PLAT188_ALERT_3_G A Non-default SIMU Restraint Value has been used     0.0200 Report
PLAT301_ALERT_3_G Main Residue  Disorder .............(Resd    1)        15% Note
PLAT410_ALERT_2_G Short Intra H...H Contact  H20B     ..H76B     .       2.00 Ang.
                                                     x,y,z  =      1_555 Check
PLAT412_ALERT_2_G Short Intra XH3 .. XHn     H62C     ..H74C     .       2.00 Ang.
```

```
                                                   x,y,z  =      1_555 Check
PLAT413_ALERT_2_G Short Inter XH3 .. XHn     H10A     ..H82D     .       2.03 Ang.
                                                   x,y,z  =      1_555 Check
PLAT606_ALERT_4_G Solvent Accessible VOID(S) in Structure ........          ! Info
PLAT860_ALERT_3_G Number of Least-Squares Restraints ............        1108 Note
PLAT868_ALERT_4_G ALERTS Due to the Use of _smtbx_masks Suppressed          ! Info
PLAT912_ALERT_4_G Missing # of FCF Reflections Above STh/L=  0.600       2454 Note
PLAT913_ALERT_3_G Missing # of Very Strong Reflections in FCF ....          2 Note
                2  0  0,  -1  0  2,
PLAT941_ALERT_3_G Average HKL Measurement Multiplicity ..........         4.5 Low
PLAT969_ALERT_5_G The 'Henn et al.' R-Factor-gap value ..........       2.665 Note
              Predicted wR2: Based on SigI**2  2.70 or SHELX Weight  6.68
PLAT978_ALERT_2_G Number C-C Bonds with Positive Residual Density.          4 Info
```

```
   0 ALERT level A = Most likely a serious problem - resolve or explain
   0 ALERT level B = A potentially serious problem, consider carefully
   6 ALERT level C = Check. Ensure it is not caused by an omission or oversight
  25 ALERT level G = General information/check it is not something unexpected

   4 ALERT type 1 CIF construction/syntax error, inconsistent or missing data
  11 ALERT type 2 Indicator that the structure model may be wrong or deficient
   8 ALERT type 3 Indicator that the structure quality may be low
   7 ALERT type 4 Improvement, methodology, query or suggestion
   1 ALERT type 5 Informative message, check
```

It is advisable to attempt to resolve as many as possible of the alerts in all categories. Often the minor alerts point to easily fixed oversights, errors and omissions in your CIF or refinement strategy, so attention to these fine details can be worthwhile. In order to resolve some of the more serious problems it may be necessary to carry out additional measurements or structure refinements. However, the purpose of your study may justify the reported deviations and the more serious of these should normally be commented upon in the discussion or experimental section of a paper or in the "special_details" fields of the CIF. checkCIF was carefully designed to identify outliers and unusual parameters, but every test has its limitations and alerts that are not important in a particular case may appear. Conversely, the absence of alerts does not guarantee there are no aspects of the results needing attention. It is up to the individual to critically assess their own results and, if necessary, seek expert advice.

Publication of your CIF in IUCr journals

A basic structural check has been run on your CIF. These basic checks will be run on all CIFs submitted for publication in IUCr journals (*Acta Crystallographica*, *Journal of Applied Crystallography*, *Journal of Synchrotron Radiation*); however, if you intend to submit to *Acta Crystallographica Section C* or *E* or *IUCrData*, you should make sure that full publication checks are run on the final version of your CIF prior to submission.

Publication of your CIF in other journals

Please refer to the *Notes for Authors* of the relevant journal for any special instructions relating to CIF submission.

PLATON version of 02/02/2025; check.def file version of 02/02/2025

Datablock hasj231122a - ellipsoid plot

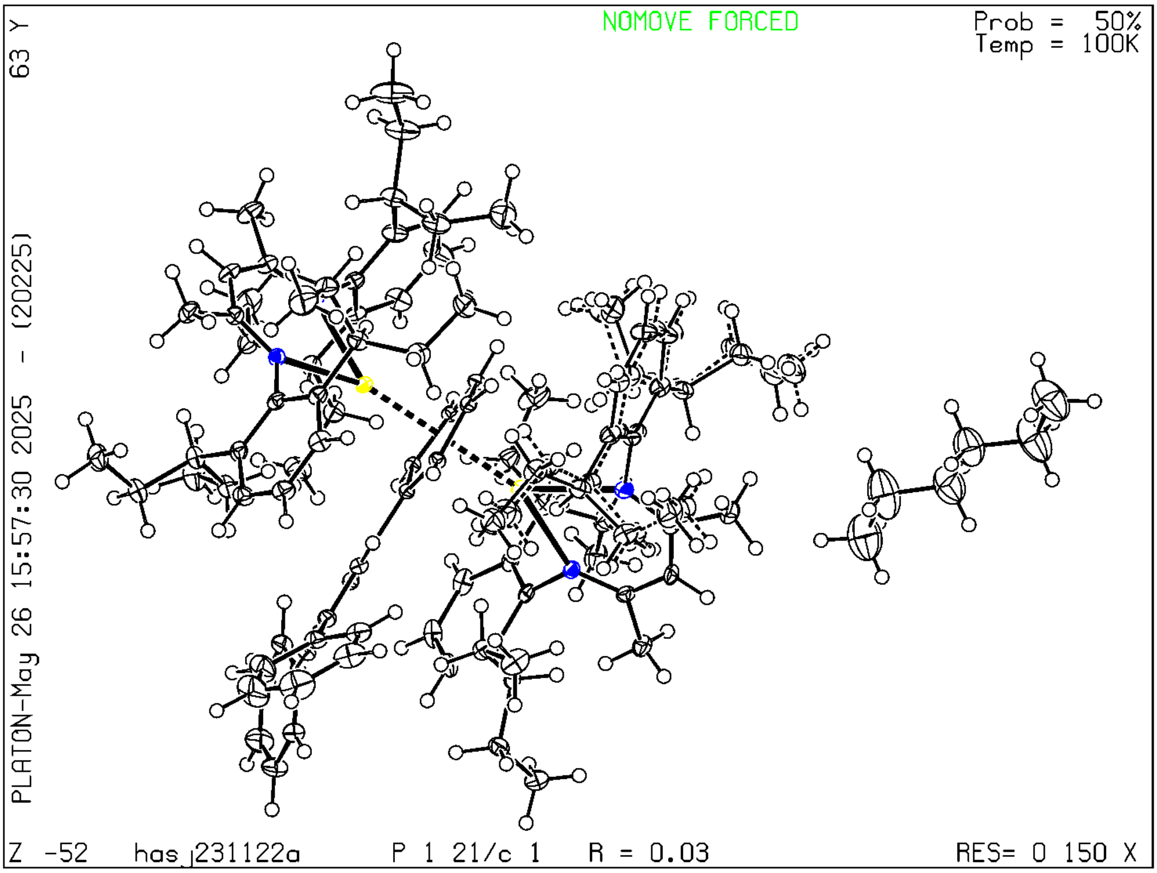

checkCIF/PLATON report

Structure factors have been supplied for datablock(s) hasj240201b

THIS REPORT IS FOR GUIDANCE ONLY. IF USED AS PART OF A REVIEW PROCEDURE FOR PUBLICATION, IT SHOULD NOT REPLACE THE EXPERTISE OF AN EXPERIENCED CRYSTALLOGRAPHIC REFEREE.

No syntax errors found. CIF dictionary Interpreting this report

Datablock: hasj240201b

Bond precision: C-C = 0.0084 A Wavelength=1.54184

Cell:	a=14.2805(7)	b=18.6260(12)	c=19.4125(11)
	alpha=61.779(6)	beta=78.252(4)	gamma=85.582(4)
Temperature:	100 K		

	Calculated	Reported
Volume	4453.3(5)	4453.3(5)
Space group	P -1	P -1
Hall group	-P 1	-P 1
Moiety formula	2(C98 H140 N4 O2 Sm2), C5 H12	2(C98 H140 N4 O2 Sm2), C5 H12
Sum formula	C201 H292 N8 O4 Sm4	C201 H292 N8 O4 Sm4
Mr	3485.87	3485.81
Dx,g cm-3	1.300	1.300
Z	1	1
Mu (mm-1)	10.169	10.169
F000	1834.0	1834.0
F000'	1817.18	
h,k,lmax	17,23,24	17,22,23
Nref	17667	17154
Tmin,Tmax	0.356,0.768	0.369,0.892
Tmin'	0.197	

Correction method= # Reported T Limits: Tmin=0.369 Tmax=0.892
AbsCorr = GAUSSIAN

Data completeness= 0.971 Theta(max)= 72.570

R(reflections)= 0.0474(14123) wR2(reflections)= 0.1267(17154)

S = 1.022 Npar= 1063

The following ALERTS were generated. Each ALERT has the format
test-name_ALERT_alert-type_alert-level.
Click on the hyperlinks for more details of the test.

[IMAGE] **Alert level C**

```
PLAT220_ALERT_2_C NonSolvent   Resd 1  C   Ueq(max)/Ueq(min) Range        3.1 Ratio
PLAT243_ALERT_4_C High   'Solvent' Ueq as Compared to Neighbors of       C100 Check
PLAT342_ALERT_3_C Low Bond Precision on  C-C Bonds ..............    0.00837 Ang.
PLAT911_ALERT_3_C Missing FCF Refl Between Thmin & STh/L=    0.600         56 Report
              -16  6  0,  16 -6  1,  16 -6  2,  13 13  2,   3-18  3, -15  9  3,
               14 12  3,  13 13  3,   4-17  4,  13 14  4,   4-16  5,   5-16  5,
                6-16  5,   6-15  5,   7-15  5,   8-15  5, -14 11  5,  12 15  5,
                3-16  6,   4-16  6,   6-15  6,   7-15  6,  13 15  6,   8 -1  7,
              -12 14  7,  15 -5  8, -14  0  8,  14 -6  9,  14 -5 10,  14 -4 10,
               14 -4 11,  14 -3 12,  16  7 12,  15  7 13,  13 -3 14,  15  7 14,
               15  8 14,  15  9 14,  15  7 15,  15  8 15,  14 10 16,  14 11 16,
               13 13 16,  13 14 16,  12 14 17,  12 15 17,  12 14 18,  -1 14 22,
                0 14 22,   1 14 22,   0 15 22,   6 10 23,   3 12 23,   4 12 23,
                2 13 23,   3 13 23,
PLAT972_ALERT_2_C Check Calcd Resid. Dens.  0.84Ang From Sm1            -1.67 eA-3
PLAT972_ALERT_2_C Check Calcd Resid. Dens.  0.79Ang From Sm2            -1.63 eA-3
PLAT972_ALERT_2_C Check Calcd Resid. Dens.  0.82Ang From Sm2            -1.62 eA-3
PLAT976_ALERT_2_C Check Calcd Resid. Dens.  1.01Ang From N4      .      -0.73 eA-3
```

[IMAGE] **Alert level G**

```
PLAT002_ALERT_2_G Number of Distance or Angle Restraints on AtSite         29 Note
PLAT003_ALERT_2_G Number of Uiso or U(i,j)  Restrained non-H-Atoms          6 Report
PLAT164_ALERT_4_G Nr. of Refined C-H H-Atoms in Heavy-Atom Struct.         10 Note
PLAT172_ALERT_4_G The CIF-Embedded .res File Contains DFIX Records          1 Report
PLAT176_ALERT_4_G The CIF-Embedded .res File Contains SADI Records          7 Report
PLAT178_ALERT_4_G The CIF-Embedded .res File Contains SIMU Records          1 Report
PLAT187_ALERT_4_G The CIF-Embedded .res File Contains RIGU Records          1 Report
PLAT299_ALERT_4_G Atom Site Occupancy Constrained at ............         0.5 Check
              C101    H10E    H10F    H10G    H10H    H10I    H10J    H10K
              H10L
PLAT301_ALERT_3_G Main Residue  Disorder .............(Resd    1)          4% Note
PLAT302_ALERT_4_G Anion/Solvent/Minor-Residue Disorder (Resd    3)        20% Note
PLAT333_ALERT_2_G Large Aver C6-Ring C-C Dist C87      -C93_b    .       1.43 Ang.
PLAT333_ALERT_2_G Large Aver C6-Ring C-C Dist C38      -C44_a    .       1.44 Ang.
PLAT412_ALERT_2_G Short Intra XH3 .. XHn     H10J     ..H99A     .       2.13 Ang.
                                               2-x,-y,1-z  =      2_756 Check
PLAT860_ALERT_3_G Number of Least-Squares Restraints ............          89 Note
PLAT910_ALERT_3_G Missing # of FCF Reflection(s) Below Theta(Min).          4 Note
                1  0  0,   0  1  0,   0  0  1,   0  1  1,
PLAT912_ALERT_4_G Missing # of FCF Reflections Above STh/L=  0.600        445 Note
PLAT941_ALERT_3_G Average HKL Measurement Multiplicity ..........         2.9 Low
PLAT969_ALERT_5_G The 'Henn et al.' R-Factor-gap value ..........       1.876 Note
              Predicted wR2: Based on SigI**2  6.76 or SHELX Weight 12.39
PLAT978_ALERT_2_G Number C-C Bonds with Positive Residual Density.          0 Info
```

---

0 **ALERT level A** = Most likely a serious problem - resolve or explain
0 **ALERT level B** = A potentially serious problem, consider carefully
8 **ALERT level C** = Check. Ensure it is not caused by an omission or oversight

```
19 ALERT level G = General information/check it is not something unexpected

 0 ALERT type 1 CIF construction/syntax error, inconsistent or missing data
11 ALERT type 2 Indicator that the structure model may be wrong or deficient
 6 ALERT type 3 Indicator that the structure quality may be low
 9 ALERT type 4 Improvement, methodology, query or suggestion
 1 ALERT type 5 Informative message, check
```

It is advisable to attempt to resolve as many as possible of the alerts in all categories. Often the minor alerts point to easily fixed oversights, errors and omissions in your CIF or refinement strategy, so attention to these fine details can be worthwhile. In order to resolve some of the more serious problems it may be necessary to carry out additional measurements or structure refinements. However, the purpose of your study may justify the reported deviations and the more serious of these should normally be commented upon in the discussion or experimental section of a paper or in the "special_details" fields of the CIF. checkCIF was carefully designed to identify outliers and unusual parameters, but every test has its limitations and alerts that are not important in a particular case may appear. Conversely, the absence of alerts does not guarantee there are no aspects of the results needing attention. It is up to the individual to critically assess their own results and, if necessary, seek expert advice.

Publication of your CIF in IUCr journals

A basic structural check has been run on your CIF. These basic checks will be run on all CIFs submitted for publication in IUCr journals (*Acta Crystallographica*, *Journal of Applied Crystallography*, *Journal of Synchrotron Radiation*); however, if you intend to submit to *Acta Crystallographica Section C* or *E* or *IUCrData*, you should make sure that full publication checks are run on the final version of your CIF prior to submission.

Publication of your CIF in other journals

Please refer to the *Notes for Authors* of the relevant journal for any special instructions relating to CIF submission.

PLATON version of 02/02/2025; check.def file version of 02/02/2025

Datablock hasj240201b - ellipsoid plot

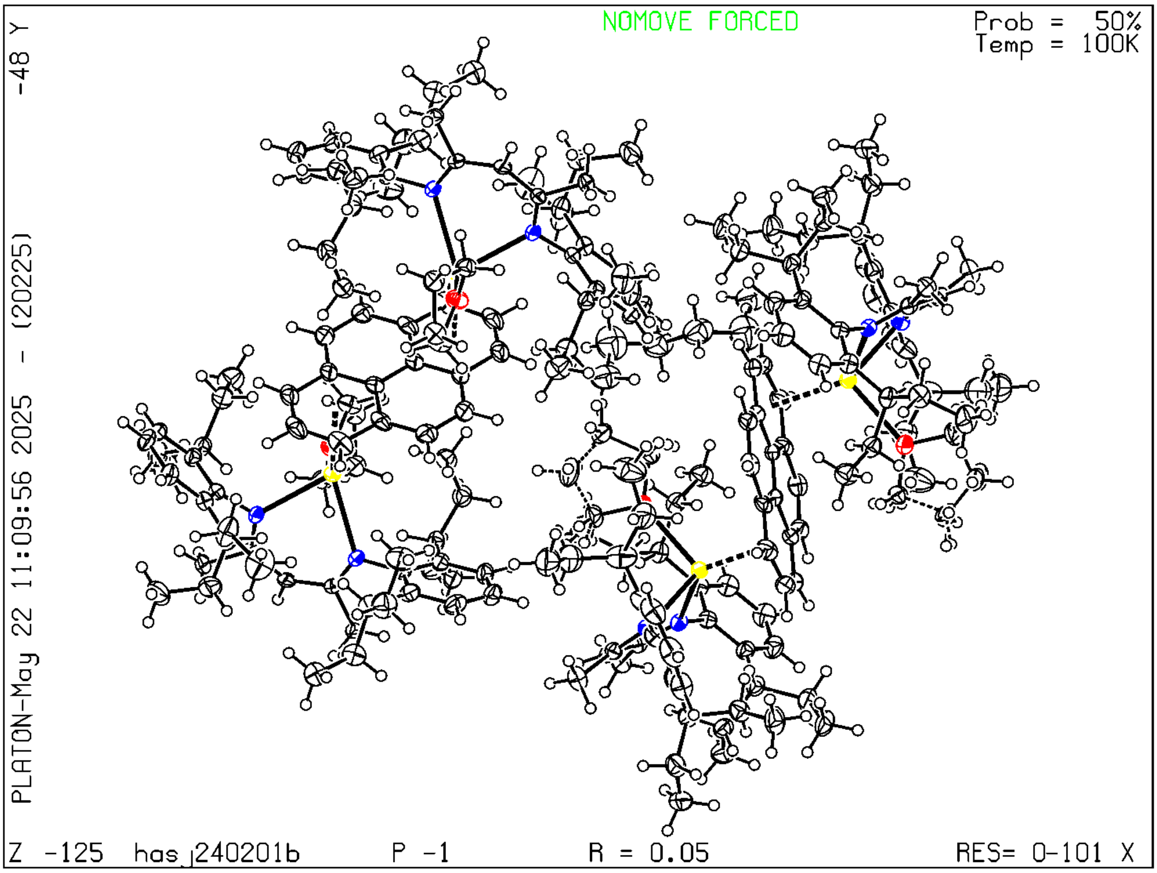

checkCIF/PLATON report

Structure factors have been supplied for datablock(s) hasj240123b

THIS REPORT IS FOR GUIDANCE ONLY. IF USED AS PART OF A REVIEW PROCEDURE FOR PUBLICATION, IT SHOULD NOT REPLACE THE EXPERTISE OF AN EXPERIENCED CRYSTALLOGRAPHIC REFEREE.

No syntax errors found.   CIF dictionary   Interpreting this report

Datablock: hasj240123b

Bond precision: C-C = 0.0042 A   Wavelength=1.54184

Cell:	a=19.1538(2)	b=19.2123(2)	c=22.5678(2)
	alpha=90	beta=101.045(1)	gamma=90
Temperature:	100 K		

	Calculated	Reported
Volume	8150.86(14)	8150.86(14)
Space group	P 21/c	P 1 21/c 1
Hall group	-P 2ybc	-P 2ybc
Moiety formula	C89 H138 N4 O2 Sm2	C89 H138 N4 O2 Sm2
Sum formula	C89 H138 N4 O2 Sm2	C89 H138 N4 O2 Sm2
Mr	1596.76	1596.73
Dx,g cm-3	1.301	1.301
Z	4	4
Mu (mm-1)	11.061	11.061
F000	3360.0	3360.0
F000'	3325.70	
h,k,lmax	23,23,27	23,23,27
Nref	16182	15896
Tmin,Tmax	0.220,0.320	0.129,0.761
Tmin'	0.039	

Correction method= # Reported T Limits: Tmin=0.129 Tmax=0.761
AbsCorr = GAUSSIAN

Data completeness= 0.982 Theta(max)= 72.613

R(reflections)= 0.0298(14222) wR2(reflections)= 0.0744(15896)

S = 1.019 Npar= 934

The following ALERTS were generated. Each ALERT has the format
test-name_ALERT_alert-type_alert-level.
Click on the hyperlinks for more details of the test.

Alert level A

```
PLAT330_ALERT_2_A Large Aver Phenyl C-C Dist C38      --C43      .       1.45 Ang.
```

Author Response: This test is not applicable here, since it is meant for normal aryl rings, but not for reduced aryl groups. It is well-known that aromatic rings expand when they are reduced.

Alert level C

```
PLAT220_ALERT_2_C NonSolvent   Resd 1  C   Ueq(max)/Ueq(min) Range        3.3 Ratio
PLAT222_ALERT_3_C NonSolvent Resd 1  H   Uiso(max)/Uiso(min) Range        4.2 Ratio
PLAT911_ALERT_3_C Missing FCF Refl Between Thmin & STh/L=    0.600         11 Report
               20  9  0,  20 10  0, -20  9  1,  20  9  1, -20 10  1,  20 10  1,
              -21  8  2, -21  9  2, -21  9  3,   9  0 22, -14  0 24,
```

Alert level G

```
PLAT143_ALERT_4_G s.u. on c - Axis Small or Missing .............    0.00020 Ang.
PLAT164_ALERT_4_G Nr. of Refined C-H H-Atoms in Heavy-Atom Struct.         5 Note
PLAT232_ALERT_2_G Hirshfeld Test Diff (M-X)  Sm2      --C38      .       5.8 s.u.
PLAT301_ALERT_3_G Main Residue  Disorder .............(Resd    1)         2% Note
PLAT910_ALERT_3_G Missing FCF Reflection(s) Below Theta(Min)[Deg]=      3.61 Note
                1  0  0,   1  1  0,   0  1  1,
PLAT912_ALERT_4_G Missing # of FCF Reflections Above STh/L=  0.600       272 Note
PLAT941_ALERT_3_G Average HKL Measurement Multiplicity ..........        3.9 Low
PLAT969_ALERT_5_G The 'Henn et al.' R-Factor-gap value ..........      1.766 Note
              Predicted wR2: Based on SigI**2  4.21 or SHELX Weight  7.30
PLAT978_ALERT_2_G Number C-C Bonds with Positive Residual Density.         0 Info
```

---

1 **ALERT level A** = Most likely a serious problem - resolve or explain
0 **ALERT level B** = A potentially serious problem, consider carefully
3 **ALERT level C** = Check. Ensure it is not caused by an omission or oversight
9 **ALERT level G** = General information/check it is not something unexpected

0 ALERT type 1 CIF construction/syntax error, inconsistent or missing data
4 ALERT type 2 Indicator that the structure model may be wrong or deficient
5 ALERT type 3 Indicator that the structure quality may be low
3 ALERT type 4 Improvement, methodology, query or suggestion
1 ALERT type 5 Informative message, check

It is advisable to attempt to resolve as many as possible of the alerts in all categories. Often the minor alerts point to easily fixed oversights, errors and omissions in your CIF or refinement strategy, so attention to these fine details can be worthwhile. It is up to the individual to critically assess their own results and, if necessary, seek expert advice.

PLATON version of 04/06/2025; check.def file version of 30/05/2025

duplicate check

No duplication found

Datablock hasj240123b - ellipsoid plot

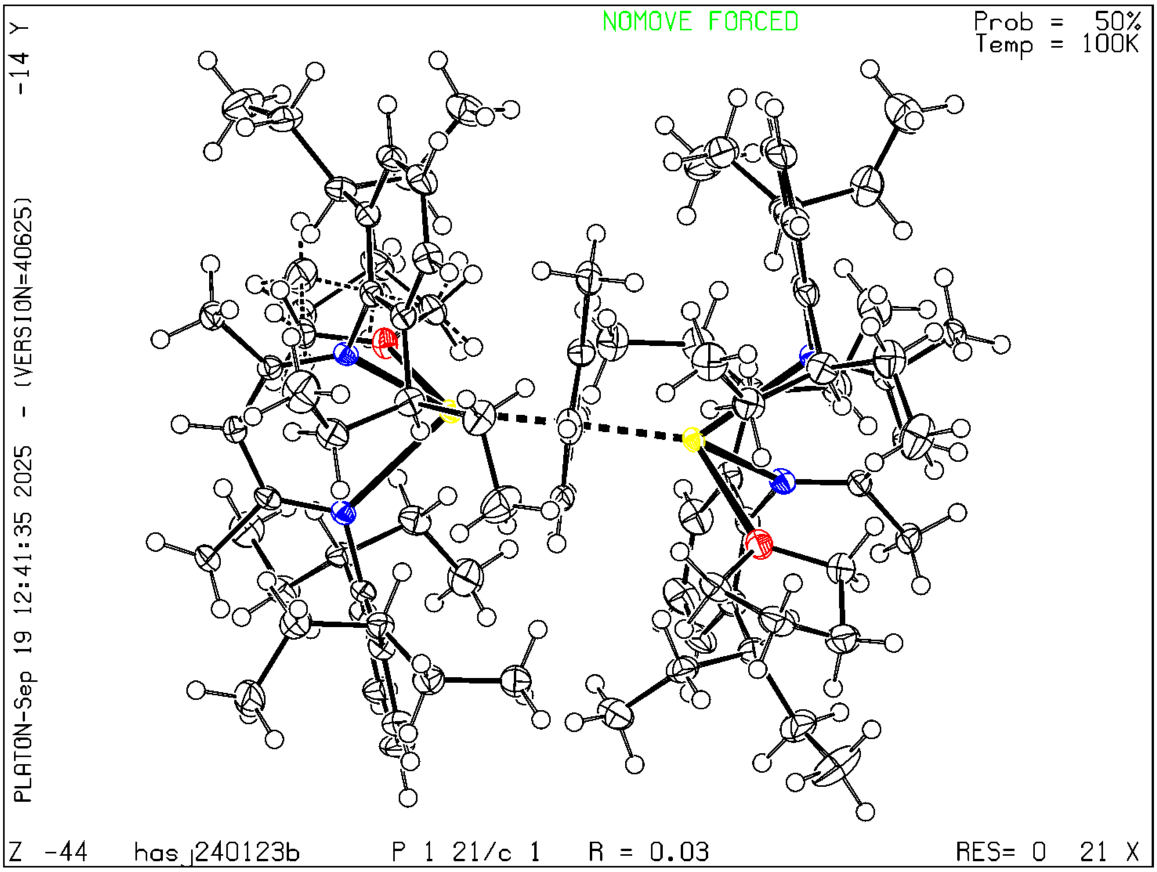

checkCIF/PLATON report

Structure factors have been supplied for datablock(s) hasj250522a

THIS REPORT IS FOR GUIDANCE ONLY. IF USED AS PART OF A REVIEW PROCEDURE FOR PUBLICATION, IT SHOULD NOT REPLACE THE EXPERTISE OF AN EXPERIENCED CRYSTALLOGRAPHIC REFEREE.

No syntax errors found. CIF dictionary Interpreting this report

Datablock: hasj250522a

```
Bond precision:   C-C = 0.0054 A          Wavelength=1.54184

Cell:            a=12.9066(4)       b=12.9725(4)      c=14.4672(4)
                 alpha=79.367(2)    beta=83.551(2)    gamma=60.711(3)
Temperature:     100 K
```

	Calculated	Reported
Volume	2075.64(12)	2075.64(12)
Space group	P -1	P -1
Hall group	-P 1	-P 1
Moiety formula	C94 H140 N4 O2 Yb2	C94 H140 N4 O2 Yb2
Sum formula	C94 H140 N4 O2 Yb2	C94 H140 N4 O2 Yb2
Mr	1704.18	1704.17
Dx,g cm-3	1.363	1.363
Z	1	1
Mu (mm-1)	4.425	4.425
F000	888.0	888.0
F000'	875.38	
h,k,lmax	15,15,17	15,15,17
Nref	8119	7922
Tmin,Tmax	0.541,0.978	0.538,1.000
Tmin'	0.490	

Correction method= # Reported T Limits: Tmin=0.538 Tmax=1.000
AbsCorr = GAUSSIAN

Data completeness= 0.976 Theta(max)= 71.824

R(reflections)= 0.0314(7211) wR2(reflections)= 0.0795(7922)

S = 1.028 Npar= 527

The following ALERTS were generated. Each ALERT has the format
test-name_ALERT_alert-type_alert-level.
Click on the hyperlinks for more details of the test.

Alert level B

```
PLAT330_ALERT_2_B Large Aver Phenyl C-C Dist C38      --C43      .       1.42 Ang.
```

Author Response: This test is not applicable here, since it is meant for normal aryl rings, but not for reduced aryl groups. It is well-known that aromatic rings expand when they are reduced.

Alert level C

```
PLAT911_ALERT_3_C Missing FCF Refl Between Thmin & STh/L=    0.600         17 Report
               15  5  0,  -8-15  1,  -7-15  1,  -6-15  1,  -1 13  1,  -1 13  2,
                1 14  2,  10 -5  3,  -1 13  3,   0 13  7, -11 -9  9,   1 13  9,
                2 -8 11,   6 -2 13,   7  0 14,   8  0 14,   8  2 15,
PLAT971_ALERT_2_C Check Calcd Resid. Dens.  0.90Ang From Yb1            1.77 eA-3
```

Alert level G

```
PLAT002_ALERT_2_G Number of Distance or Angle Restraints on AtSite          9 Note
PLAT003_ALERT_2_G Number of Uiso or U(i,j)  Restrained non-H-Atoms          9 Report
PLAT164_ALERT_4_G Nr. of Refined C-H H-Atoms in Heavy-Atom Struct.          5 Note
PLAT176_ALERT_4_G The CIF-Embedded .res File Contains SADI Records          5 Report
PLAT178_ALERT_4_G The CIF-Embedded .res File Contains SIMU Records          1 Report
PLAT187_ALERT_4_G The CIF-Embedded .res File Contains RIGU Records          1 Report
PLAT188_ALERT_3_G A Non-default SIMU Restraint Value has been used     0.0300 Report
PLAT301_ALERT_3_G Main Residue  Disorder .............(Resd    1)         8% Note
PLAT860_ALERT_3_G Number of Least-Squares Restraints .............        251 Note
PLAT910_ALERT_3_G Missing FCF Reflection(s) Below Theta(Min)[Deg]=       3.93 Note
                0  0  1,
PLAT912_ALERT_4_G Missing # of FCF Reflections Above STh/L=  0.600        178 Note
PLAT941_ALERT_3_G Average HKL Measurement Multiplicity ..........         3.3 Low
PLAT969_ALERT_5_G The 'Henn et al.' R-Factor-gap value ..........       1.933 Note
              Predicted wR2: Based on SigI**2  4.11 or SHELX Weight  7.73
PLAT978_ALERT_2_G Number C-C Bonds with Positive Residual Density.          0 Info
```

---

0 **ALERT level A** = Most likely a serious problem - resolve or explain
1 **ALERT level B** = A potentially serious problem, consider carefully
2 **ALERT level C** = Check. Ensure it is not caused by an omission or oversight
14 **ALERT level G** = General information/check it is not something unexpected

```
0 ALERT type 1 CIF construction/syntax error, inconsistent or missing data
5 ALERT type 2 Indicator that the structure model may be wrong or deficient
6 ALERT type 3 Indicator that the structure quality may be low
5 ALERT type 4 Improvement, methodology, query or suggestion
1 ALERT type 5 Informative message, check
```

It is advisable to attempt to resolve as many as possible of the alerts in all categories. Often the minor alerts point to easily fixed oversights, errors and omissions in your CIF or refinement strategy, so attention to these fine details can be worthwhile. It is up to the individual to critically assess their own results and, if necessary, seek expert advice.

PLATON version of 04/06/2025; check.def file version of 30/05/2025

---

duplicate check

No duplication found

Datablock hasj250522a - ellipsoid plot

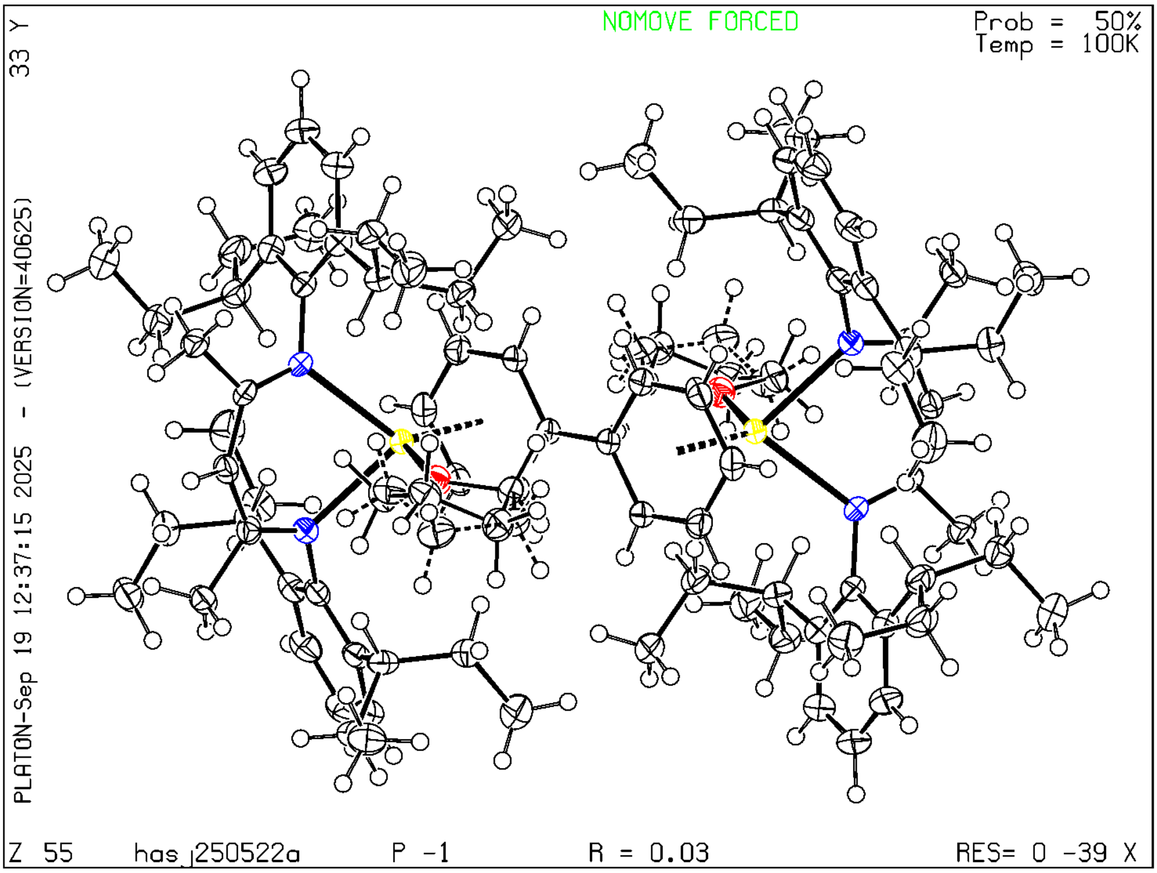

Supplement: Supplementary file 2 — Supporting Information [file CHEM-31-e02710-s001.zip › Combined checkcif report.pdf]
